# Multiomics analysis unveils an inosine-sensitive DNA damage response in neurogenic bladder after spinal cord injury

**DOI:** 10.1172/jci.insight.180275

**Published:** 2025-05-08

**Authors:** Ali Hashemi Gheinani, Bryan S. Sack, Alexander Bigger-Allen, Hatim Thaker, Hussein Atta, George Lambrinos, Kyle Costa, Claire Doyle, Mehrnaz Gharaee-Kermani, Susan Patalano, Mary Piper, Justin F. Cotellessa, Dijana Vitko, Haiying Li, Manubhai Kadayil Prabhakaran, Vivian Cristofaro, John Froehlich, Richard S. Lee, Wei Yang, Maryrose P. Sullivan, Jill A. Macoska, Rosalyn M. Adam

**Affiliations:** 1 Urological Diseases Research Center, Boston Children’s Hospital, Boston, Massachusetts, USA.; 2 Functional Urology Research Group, Department for BioMedical Research DBMR, University of Bern, Switzerland.; 3 Department of Urology, Inselspital University Hospital, Bern, Switzerland.; 4 Department of Surgery, Harvard Medical School, Boston, Massachusetts, USA.; 5 Broad Institute of MIT and Harvard, Cambridge, Massachusetts, USA.; 6 Center for Personalized Cancer Therapy, University of Massachusetts, Boston, Massachusetts, USA.; 7 Harvard Chan Bioinformatics Core, Harvard T.H. Chan School of Public Health, Boston, Massachusetts, USA.; 8 Division of Urology, VA Boston Healthcare System, Boston, Massachusetts, USA.; 9 Department of Pathology, Stony Brook Medicine, Stony Brook University, Stony Brook, New York, USA.

**Keywords:** Cell biology, Muscle biology, Bioinformatics, DNA repair, Urology

## Abstract

Spinal cord injury (SCI) evokes profound dysfunction in hollow organs such as the urinary bladder and gut. Current treatments are limited by a lack of molecular data to inform novel therapeutic avenues. Previously, we showed that systemic treatment with the neuroprotective agent inosine improved bladder function following SCI in rats. Here, we applied integrated multi-omics analysis to explore molecular alterations in the bladder over time and their sensitivity to inosine following SCI. Canonical signaling pathways regulated by SCI included those associated with protein synthesis, neuroplasticity, wound healing, and neurotransmitter degradation. Upstream regulator and causal network analysis predicted multiple effectors of DNA damage response signaling following injury, including poly-ADP ribose phosphorylase-1 (PARP1). Markers of DNA damage (γH2AX, ATM/ATR substrates) and PARP activity were increased in bladder tissue following SCI and attenuated with inosine treatment. Inosine treatment also attenuated oxidative DNA damage in rat bladder cells in vitro. Proteomics analysis suggested that SCI induced changes in protein synthesis–, neuroplasticity-, and oxidative stress–associated pathways, a subset of which were shown in transcriptomics data to be inosine sensitive. These findings provide insights into the molecular landscape of the bladder following SCI and identify key inosine-sensitive pathways associated with injury.

## Abstract 2

Multi-omics analysis reveals oxidative stress and DNA damage as inosine-sensitive responses to spinal cord injury in the bladder.

## Graphical abstract

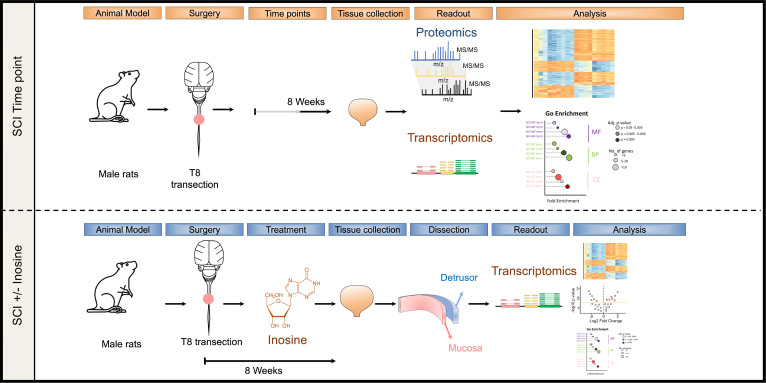

## Introduction

Damage to the spinal cord has a profound effect on motor, sensory, and autonomic innervation. Depending on the level and extent, injury can result in limb paralysis, bladder/bowel dysfunction, and development of neuropathic pain (reviewed in ref. 1). Interruption of neural control to the lower urinary tract frequently leads to reflex bladder contractions termed neurogenic detrusor overactivity (NDO) and detrusor-sphincter-dyssynergia (DSD) that reflects disruption of the normal coordination between bladder and urethral sphincter required for efficient voiding. NDO and DSD are associated with the emergence of lower urinary tract symptoms including urinary incontinence, incomplete bladder emptying resulting in urinary retention, and an increased risk of urinary tract infection and renal damage (reviewed in ref. 2). Clinical management of neurogenic lower urinary tract dysfunction (LUTD) focuses primarily on catheterization to promote efficient bladder emptying and pharmacological intervention to diminish NDO. Agents approved for treatment of LUTD include onabotulinumtoxin A to inhibit neurotransmitter release, antimuscarinic agents to block muscarinic receptor-mediated detrusor contraction, and b-adrenergic receptor agonists to promote detrusor relaxation (3). These interventions decrease bladder storage pressure, enhance bladder capacity, and diminish incontinence.

The functional obstruction that results from DSD also provokes marked changes in bladder morphology, consistent with those observed following anatomic obstruction — e.g., in response to prostatic enlargement in men. Characteristic changes include bladder wall thickening as a result of cellular hypertrophy and hyperplasia (4), as well as enhanced deposition and turnover of extracellular matrix (ECM) leading to fibrosis (5, 6). With the advent of genome-wide expression profiling, several groups have begun to characterize the molecular landscape that underlies the response of the bladder to spinal cord damage in animal models (7–10) and in smooth muscle cells isolated from human bladder biopsies (11, 12). In addition to verification of altered expression of ECM proteins and regulators, microarray-based expression profiling of bladder tissue from spinal cord–injured rats also provided important insights into additional drivers of neurogenic bladder pathophysiology including the profibrotic regulator TGF-β1 and proinflammatory mediators such as IL-1β and S100A8/A9 (7, 8). In those studies, pathway analysis identified the enrichment for processes associated with remodeling such as proliferation and connective tissue deposition, as well as a progressive increase in inflammatory processes following injury. In spite of its deleterious effect on bladder compliance and function, however, no pharmacological agents that target fibroproliferative remodeling have been approved to treat LUTD. While these studies have provided valuable insights into molecular alterations associated with spinal cord injury (SCI), the lack of high-resolution, multidimensional, and network-level information has hindered the development of rationally designed therapeutic interventions targeting bladder wall remodeling.

Previously, we demonstrated a marked improvement in bladder function in rats with functional bladder outlet obstruction secondary to SCI treated with inosine (13), a purine nucleoside with neuroprotective, neurotrophic, antiinflammatory, and antioxidant properties (reviewed in ref. 14). In that study, rats with SCI receiving inosine daily for 6 weeks either immediately after injury or following a 2-month delay displayed a decrease in NDO. Consistent with the neuroprotective activity of inosine reported previously, we observed preservation of the neuronal markers synaptophysin and NF200 within the bladders of inosine-treated rats, as well as decreased staining for TRPV1, a marker of C-fibers implicated in the development of NDO following injury (reviewed in ref. 15).

To explore temporal changes in the bladder following SCI and determine the molecular basis for the beneficial effect of inosine on bladder function observed previously, we conducted a multi-omics analysis to systematically evaluate the genes, pathways and signaling networks that are differentially regulated in the bladder in response to SCI and their modulation with inosine treatment. This multi-omics analysis has provided insights into the biological processes perturbed in the bladder in response to SCI, implicating DNA damage in pathologic changes resulting from injury and attenuation of such damage with inosine treatment.

## Results

### SCI evokes time-dependent changes in the bladder transcriptome.

**Figure 1:**
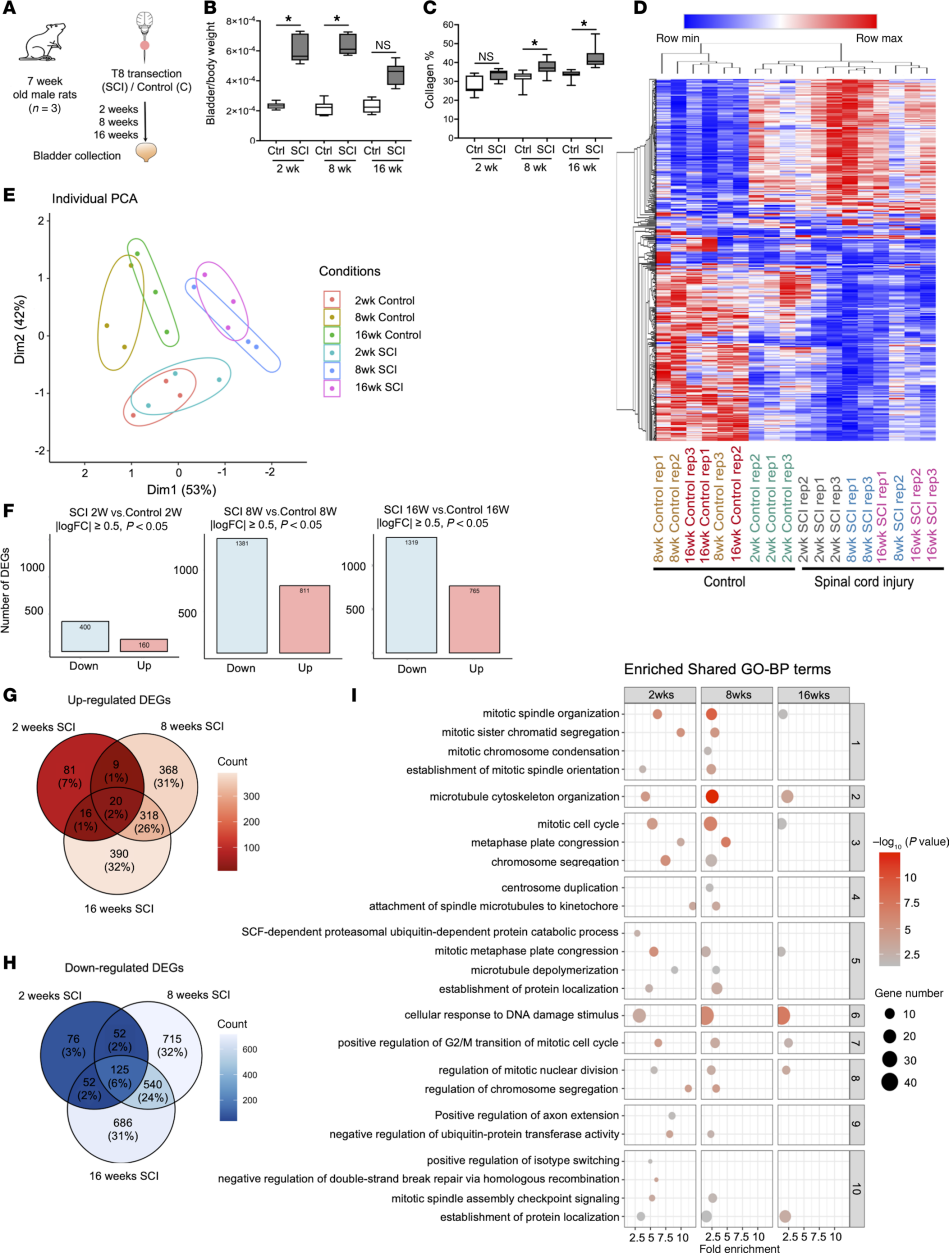
Effect of spinal cord injury over time on the transcriptome of the bladder. (**A**) Experimental design. (**B** and **C**) Bladder/body weight measurement (**B**) and measurement of collagen deposition from MTS-stained sections (**C**). Significance was determined by 1-way ANOVA followed by Tukey’s multiple comparisons test. *P < 0.05, *n* = 6–7 replicates. (**D**) Heatmap and hierarchical clustering using top 500 DEGs in different comparisons groups; log_2_ greater than +0.05 and less than –0.05 (> ±0.05); *P* < 0.05; read counts > 1,000. (**E**) PCA based on count matrix of all genes. (**F**) Bar charts indicating up- and downregulated DEGs at each time point. (**G** and **H**) Venn diagrams of upregulated and downregulated DEGs at each time point following SCI compared with their respective controls. (**I**) Enrichment analysis of DEGs from each time point of SCI using gene ontology (GO) terms for biological process (BP).

To understand global, temporal changes in the molecular landscape of the bladder following SCI in rats, we first analyzed the transcriptome at 2, 8 and 16 weeks following midthoracic spinal cord transection, compared with noninjured controls (Figure 1A). The earliest time point chosen (2 weeks) corresponds to the end of the spinal shock phase, whereas the later time points represent established (8-week) and chronic (16-week) phases of the response to SCI. The bladder/body weight ratios as well as collagen deposition were significantly higher at each time point in SCI rats compared with controls (Figure 1, B and C), consistent with the fibroproliferative remodeling that is known to occur following obstruction (4, 16, 17). RNA was isolated from full-thickness bladder tissues and subjected to RNA-Seq to identify differentially expressed genes (DEGs). The heatmap and hierarchical clustering based on the top most variable genes in the whole dataset (18 samples) indicated 2 main clusters comprising control and spinal cord–injured samples. Distinct temporal differences were observed in which the 2-week controls were separated from the 8- and 16-week controls. This separation was also observed between the 2-week SCI subgroup from the 8- and 16-week SCI subgroup. (Figure 1D). The separation of spinal cord–injured and control groups based on DEGs was also confirmed by principal component analysis (PCA) (Figure 1E). DEG analysis identified 560, 2,192, and 2,084 DEGs in the bladder at 2, 8 and 16 weeks after SCI, respectively (Figure 1F). Comparing DEGs across time points revealed 20 upregulated and 125 downregulated genes shared across all 3 time points of SCI compared with their respective controls (Figure 1, G and H). Expression of a subset of DEGs was validated using NanoString analysis of RNA from control and SCI rat bladders at each of the 3 time points. Linear regression analysis showed strong correlation between RNA-Seq and NanoString data, as indicated by Pearson correlation coefficients and *P* values (Supplemental Figure 1, A–C; supplemental material available online with this article; https://doi.org/10.1172/jci.insight.180275DS1). This high concordance supports the robustness of the transcriptomic findings and supports the reproducibility of DEG identification over time after injury. Volcano plots (Supplemental Figure 1 D–F) and scatterplots (Supplemental Figure 1, G–I) identified DEGs at each time point that were both robustly expressed and regulated, suggesting they are biologically meaningful in the context of SCI.

To address transcriptomic similarities beyond direct comparisons at the gene level, we leveraged enrichment analyses using Gene Ontology (GO) terms for Biological Process (BP) and hierarchical clustering. These analyses identified “cellular response to DNA damage stimulus” and double-strand break repair, which increased in significance in a time-dependent manner (Figure 1I). Both terms were clustered in the top 10 clusters after hierarchical clustering analysis. Similarly, enrichment analysis using GO terms for molecular function and KEGG identified time-dependent changes in the fold enrichment of DNA modification terms such as “histone deacetylase binding”, “chromatin binding”, and “DNA transcription factor binding (Supplemental Figure 2A) as well as “Cell Cycle”, “p53 signaling”, and “Cellular Senescence” (Supplemental Figure 2, A and B). Enrichment analysis using GO terms for cellular compartment (CC) suggest these DNA-related changes are broadly affecting structures necessary for cell cycle maintenance (Supplemental Figure 2C). At 2 weeks after SCI, minimal enrichment was observed, with the majority of GO terms being in the CC category. By 8 and 16 weeks following SCI, an increase in the number of enriched DNA damage–related GO terms was identified, predominantly within the BP category, indicating a progressive involvement of DNA damage–related processes in the bladder over time following injury (Supplemental Figure 2D). Consistent with the neurogenic injury and known morphological changes in the bladder that arise following functional obstruction, Ingenuity Pathway Analysis (IPA) identified pathways related to wound healing (GP6 signaling, wound healing) and innervation (axonal guidance, neurotrophin/TRK signaling) among the significantly regulated pathways common to all 3 time points (Supplemental Figure 3, A–C).

### Inosine prevents SCI-induced transcriptome changes in detrusor and mucosa.

**Figure 2:**
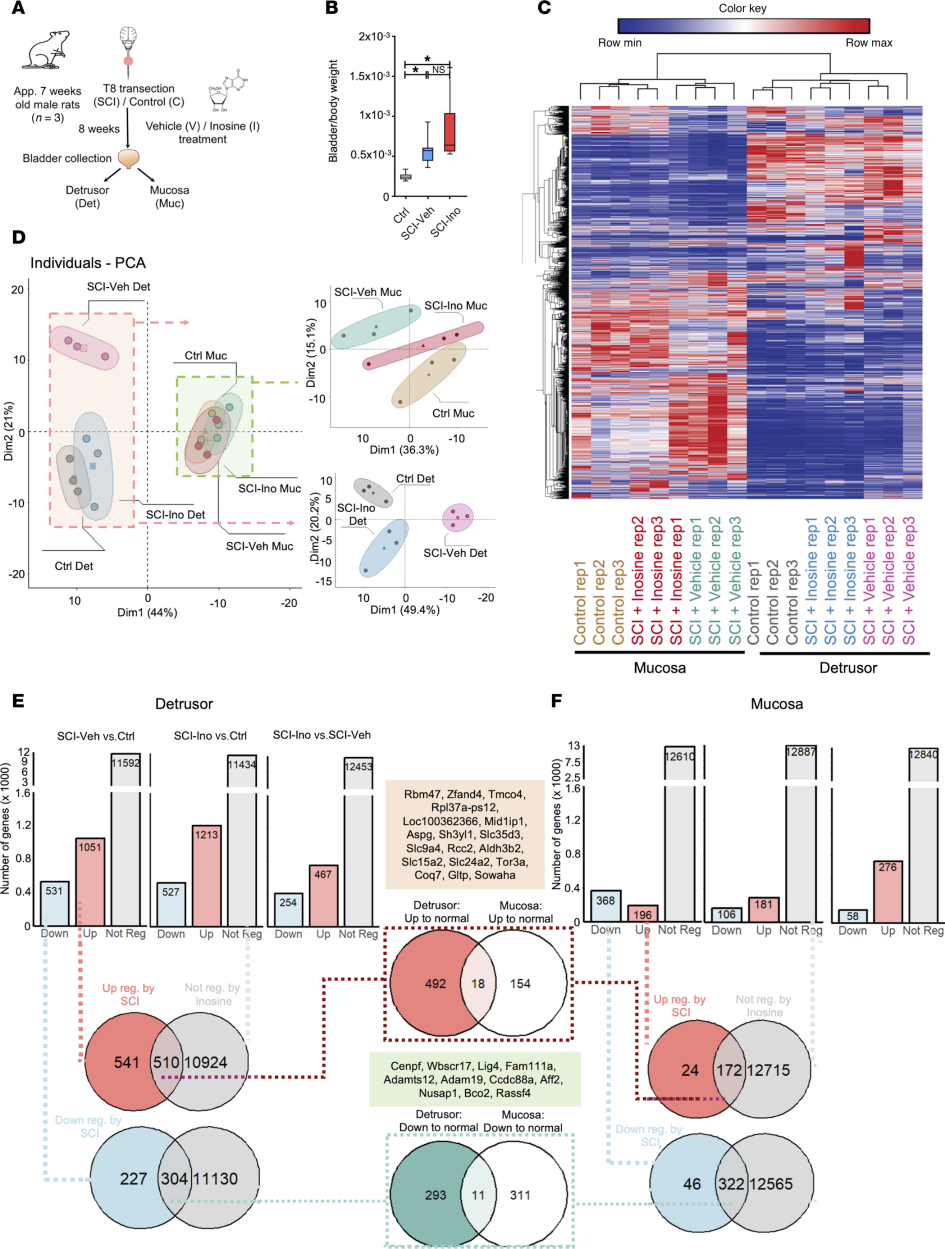
Effect of spinal cord injury and inosine treatment on transcriptome of bladder. (**A**) Experimental design. (**B**) Bladder/body weight measurement. Significance was determined by 1-way ANOVA followed by Tukey’s multiple comparisons test. *P < 0.05, *n* = 10–13 replicates. (**C**) Heatmap and hierarchical clustering using DEGs in different comparisons groups; log_2_ fold change> ± 0.5; *P* < 0.05; read counts cpm > 1. (**D**) PCA based on top 200 variable genes. (**E** and **F**) Bar charts indicating up-, down-, and nonregulated DEGs in detrusor (**E**) or mucosa (**F**) across 3 comparisons: SCI-vehicle versus Control; SCI-inosine versus Control; SCI-inosine versus SCI-vehicle. Red circle (upper Venn diagram): genes upregulated in detrusor in SCI-vehicle versus Control (541 + 510 = 1,051 [red bar in graph]). Blue circle (lower Venn diagram): genes downregulated in detrusor in SCI-vehicle versus Control (227 + 304 = 531 [blue bar in graph]). Gray circles: genes not differentially regulated in SCI-inosine versus Control (510 + 10,924 = 11,434, upper Venn diagram; 304 + 11,130 = 11,434, lower Venn diagram [gray bar in graph]). (**F**) Red circle (upper Venn diagram): genes upregulated in mucosa in SCI-vehicle versus Control (24 + 172 =[196 (red bar in graph]). Blue circle (lower Venn diagram): genes down-regulated in mucosa in SCI-vehicle versus Control (46 + 322 = 368 [blue bar in graph]). Gray circles: genes not differentially regulated in SCI-inosine versus Control (172 + 12,715 = 12,887, upper Venn Diagram and 322 + 12,565 = 12,887, lower Venn diagram). Genes shared between the red/blue circles and gray circles in the Venn diagrams were considered inosine-sensitive — i.e., dysregulated — in SCI-vehicle versus Control and returned to control expression levels in SCI-inosine versus Control. This revealed 510 upregulated and 304 downregulated genes in detrusor and 172 upregulated and 322 downregulated genes in mucosa that were restored to control levels by inosine. Inosine-sensitive genes in the detrusor and the mucosa were compared via Venn diagrams in the central panels to identify those that were modulated by inosine in both tissue compartments.

Next, we explored the effect of inosine treatment on the bladder transcriptome. Previous studies from our group have shown that chronic inosine treatment can mitigate NDO in spinal cord–injured rats independently of direct effects on muscle contractility (13, 18) (Supplemental Figure 4, A–C). Whereas SCI was associated with decreased levels of synaptophysin and the Ad fiber marker NF200 in the bladder wall, compared with noninjured controls, inosine treatment partially preserved synaptophysin and NF200 levels, consistent with its known neuroprotective activity (14) (Supplemental Figure 4, B and C). To investigate the effect of inosine in the SCI bladder at a molecular level, we performed transcriptomics analysis on bladder tissue from rats subjected to spinal cord transection and treated with inosine or vehicle for 8 weeks after injury (Figure 2A). We chose the time point of 8 weeks since the DEGs identified in the time-course analysis were comparable between 8 and 16 weeks, suggesting transcriptional changes following injury had stabilized. Consistent with previous findings from us and others (16, 19, 20), the bladder/body weight ratios in SCI rats were significantly higher than in control animals, primarily as a result of increased bladder weights following injury (*P* < 0.05 in each case; Figure 2B). For transcriptomic analysis, RNA was isolated from representative detrusor and mucosa samples microdissected from bladders at the time of harvest (Figure 2A) and subjected to RNA-Seq. The detrusor comprises the smooth muscle responsible for bladder contractility, whereas the mucosa comprises the urothelium and underlying lamina propria. Our analysis pipeline is outlined in Supplemental Figure 4, D and E.

We first sought to test the effect of SCI on the transcriptome of detrusor and mucosa and the extent to which DEGs separate different samples and treatment groups from each other. The heatmap and hierarchical clustering based on the top most variable genes in the whole dataset (18 samples) indicated 2 main clusters comprising detrusor and mucosa samples. All subgroups of SCI with or without inosine and controls were also clustered together indicating distinct effects of both injury and inosine treatment on the bladder transcriptome (Figure 2C). The separation of detrusor from mucosa based on DEGs was also confirmed by PCA (Figure 2D). In the PCA, detrusor samples from vehicle-treated SCI rats clustered further from detrusor samples from inosine-treated SCI rats, which clustered close to controls. Using the top 200 genes did not separate mucosa samples in different treatment groups when compared with detrusor. However, when detrusor and mucosa samples were analyzed separately, we observed a complete separation of control mucosa, vehicle-treated SCI mucosa, and inosine-treated SCI mucosa (Figure 2D, upper right panel) using the same 200-gene set.

In detrusor, SCI was associated with 1,582 DEGs whereas, in mucosa, 564 DEGs were detected when compared with their respective controls (Figure 2, E and F). Of these DEGs, 1,051 of 1,582 were upregulated and 531 of 1,582 were downregulated in detrusor of SCI-vehicle animals (Figure 2E and Supplemental Figure 5, A and B), whereas 196 of 564 were upregulated and 368 of 564 were downregulated in mucosa of SCI-vehicle animals (Figure 2F and Supplemental Figure 6, A and B). In SCI-inosine animals, we identified 1,740 DEGs in detrusor (1,213 upregulated, 527 downregulated) and 287 DEGs in mucosa (181 upregulated, 106 downregulated) compared with controls (Figure 2, E and F; Supplemental Figure 5, C and D; and Supplemental Figure 6, C and D). Our analysis revealed that the expression level of 510 genes in detrusor and 172 genes in mucosa that were upregulated with SCI was preserved after inosine treatment, showing expression comparable with that observed in control samples (Figure 2, E and F, upper middle panel). Similarly, the expression level of 304 genes in detrusor and 322 genes in mucosa that were downregulated with SCI was preserved with inosine treatment, with a level similar to that in control samples (Figure 2, E and F, lower middle panel). Collectively, these genes are considered to be inosine responsive. Among the inosine-responsive genes, 29 were present in both detrusor and mucosa. These included 18 that were upregulated with injury but maintained at control levels with inosine treatment such as *Rbm47*, *Slc35d3*, and *Coq7* (Figure 2, E and F, and Supplemental Figure 7) and 11 that were downregulated with injury but were maintained at control levels with inosine (*Cenpf*, *Adamts12*, *Rassf4*) (Figure 2, E and F, and Supplemental Figure 7). Notably, some of these genes, including *Cenpf*, *Coq7*, *Rbm47*, *Rpl*, and solute carrier family (*Slc*) genes, were previously identified as inosine-responsive in neurons (21, 22), consistent with a conserved mechanism of action of inosine across different tissues.

### Inosine modulates growth- and innervation-associated pathways following injury.

**Figure 3:**
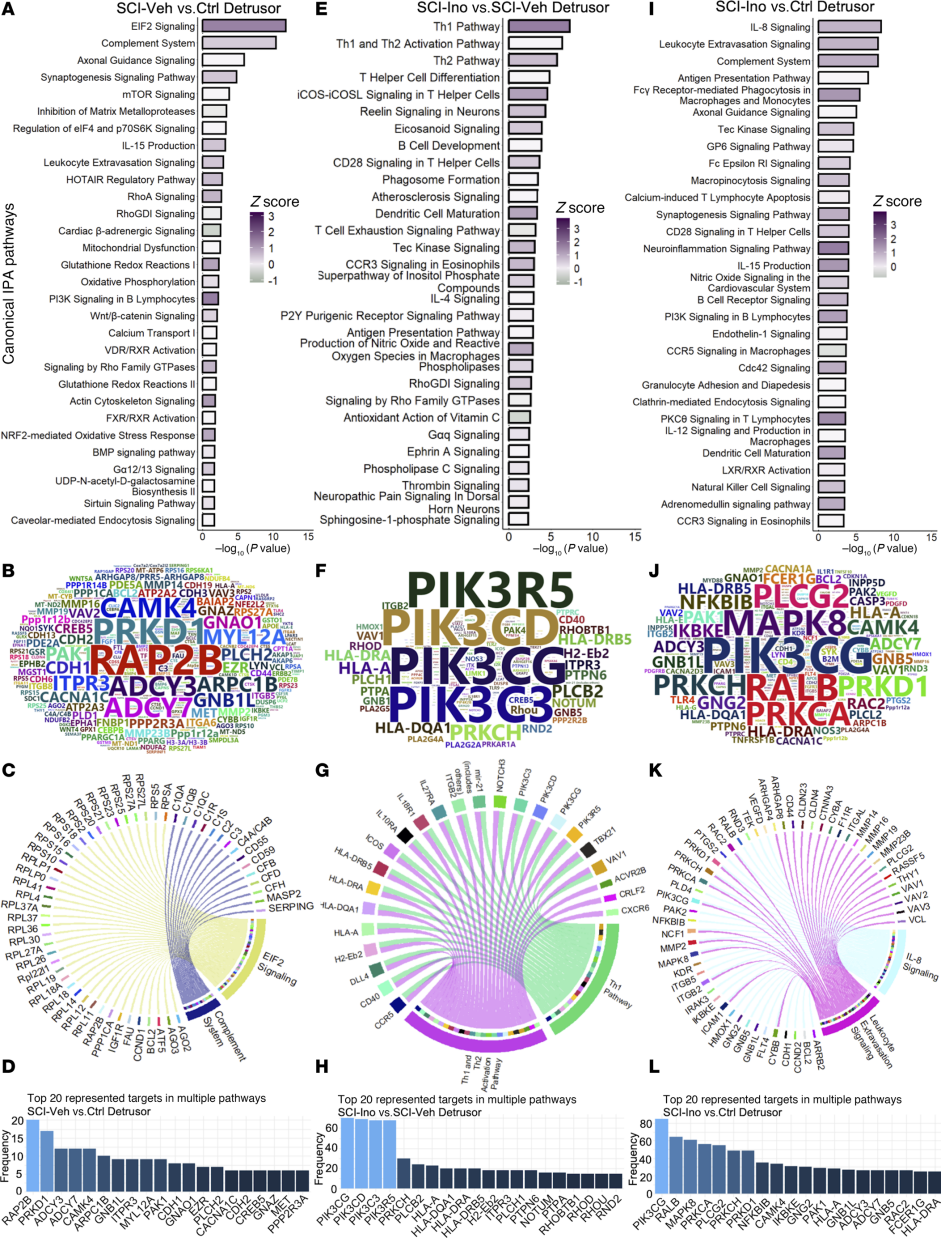
Regulated canonical pathways inferred by IPA in detrusor. (**A**) Bar chart of top 30 regulated pathways in detrusor of SCI-vehicle versus Control. (**B**) Word cloud of most frequent genes regulated and enriched in the regulated pathways. (**C**) Circos plot of top 2 pathways (based on *P* value) and enriched genes. (**D**) Bar chart visualizing the frequency of enrichment of top 20 enriched genes in pathways. (**E**) Bar chart of top 30 regulated pathways in detrusor of SCI-inosine versus SCI-vehicle. (**F**) Word cloud of most frequent genes regulated and enriched in the regulated pathways. (**G**) Circos plot of top 2 pathways (based on *P* value) and enriched genes. (**H**) Bar chart visualizing the frequency of enrichment of top 20 enriched genes in pathways. (**I**) Bar chart of top 30 regulated pathways in detrusor of SCI-inosine versus Control. (**J**) Word cloud of most frequent genes regulated and enriched in the regulated pathways. (**K**) Circos plot of top 2 pathways (based on *P* value) and enriched genes. (**L**) Bar chart visualizing the frequency of enrichment of top 20 enriched genes in pathways.

Next, we performed canonical pathway analysis to identify regulatory pathways altered in detrusor and mucosa following injury and their sensitivity to inosine treatment. The top pathways in the detrusor of SCI-vehicle versus control included EIF2 signaling, complement system, axonal guidance signaling, synaptogenesis signaling, and mTOR signaling (Figure 3A). Given their roles in regulation of protein synthesis and translational control, the emergence of EIF2 and mTOR signaling is consistent with the hypertrophy of the bladder observed following SCI. Circos plots revealed that the EIF2 signaling pathway was enriched for multiple genes encoding ribosomal proteins in agreement with its role in regulation of protein synthesis and translational control, whereas the complement system pathway harbored genes encoding complement factors and regulators (Figure 3C). Similarly, identification of axonal guidance, synaptogenesis and complement system pathways is consistent with the neuroplasticity evident in the bladder following SCI (reviewed in refs. 23, 24). To further explore the regulatory landscape, we conducted an analysis to identify the genes most frequently represented within the top 20 regulated pathways in the detrusor of vehicle-treated SCI versus controls. Genes enriched in these pathways included *Rap2b*, *Prkd1*, *Adcy3*, *Adcy7*, *Camk4*, *Arpc1b*, and *Itpr3*, suggesting their involvement in the molecular mechanisms underlying the response of the bladder to SCI (Figure 3, B and D, Supplemental Figure 8A, and Supplemental Figure 9A).

Enriched pathways in the detrusor of SCI-inosine versus SCI-vehicle included several related to immune cells including type 1 and 2 Th cells (Figure 3, E and G). In agreement with these findings, inosine has been implicated previously in regulation of immune cell signaling (25–27). The genes most frequently represented in the top regulated pathways included *Pik3cg*, *Pik3cd*, *Pik3c3*, *Pik3r5*, and *Prkch* (Figure 3, F and H), several of which have been linked to immune cell regulation (28–31). Notably, pathways that were implicated in detrusor following injury such as EIF2 signaling, mTOR signaling, and complement system were no longer enriched in detrusor following inosine treatment (Supplemental Figure 8, A and B, and Supplemental Figure 9, A and B). Therefore, processes regulated by such pathways following SCI, including protein synthesis, and cytoskeletal regulation appear to be sensitive to treatment with inosine.

Comparison of top pathways enriched in the detrusor of SCI-inosine versus Control revealed several related to inflammation and immune cell biology such as IL-8 signaling, leukocyte extravasation signaling, complement system, antigen presentation pathway, and Fcg receptor–mediated phagocytosis in macrophages and monocytes (Figure 3, I and K). The genes most frequently represented in the top regulated pathways included *Pik3cg*, *Ralb*, *Mapk8*, *Plcg2*, *Prkca*, *Prkch*, *Prkd1*, *Camk4*, *Nfkbib*, and *Ikbke*, indicating their involvement in the regulation of those identified pathways (Figure 3, J and L, Supplemental Figure 8C, and Supplemental Figure 9C).

**Figure 4:**
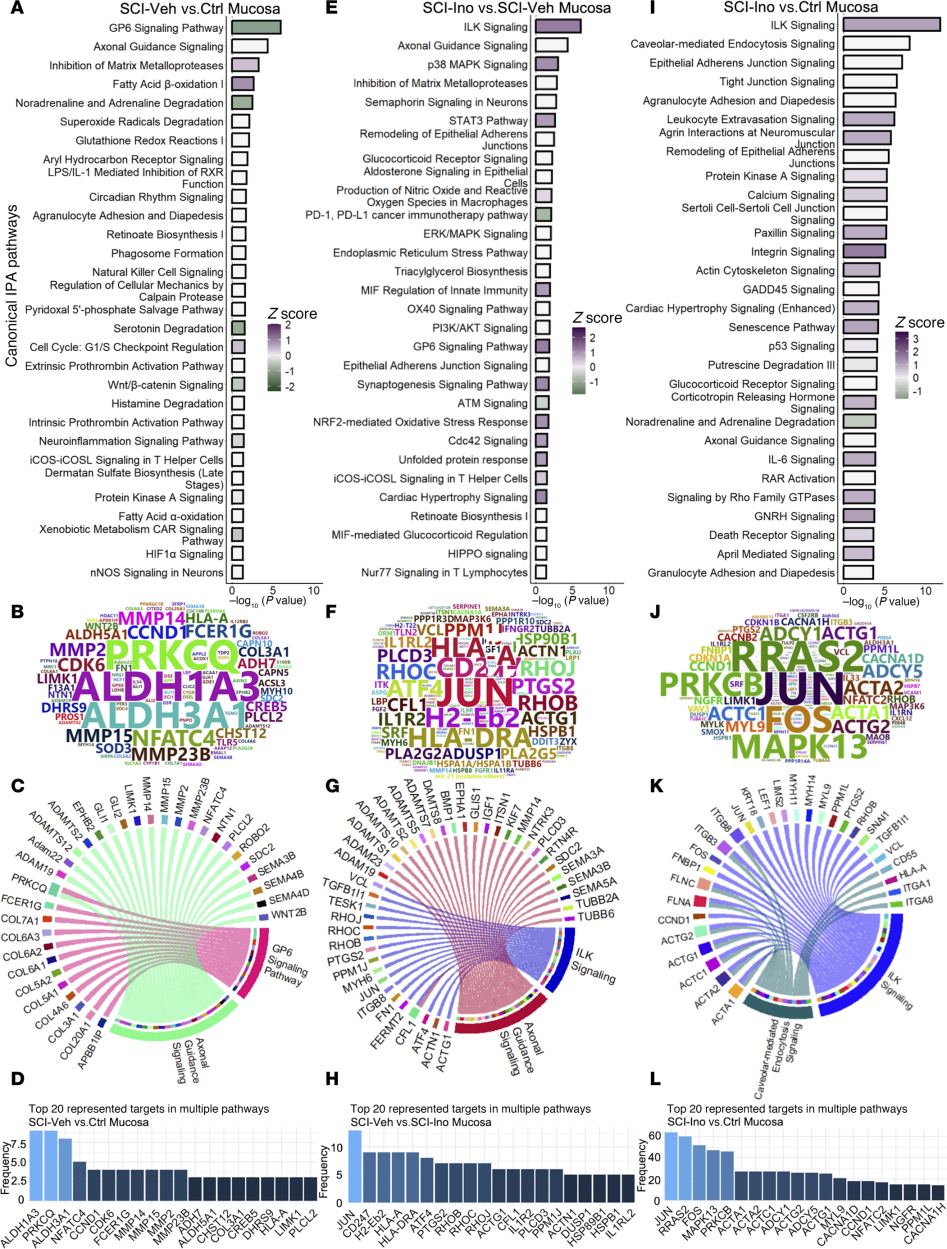
Regulated canonical pathways inferred by IPA in mucosa. (**A**) Bar chart of top 30 regulated pathways in mucosa of SCI-vehicle versus Control. (**B**) Word cloud of most frequent genes regulated and enriched in the regulated pathway. (**C**) Circos plot of top 2 pathways (based on *P* value) and enriched genes. (**D**) Bar chart visualizing the frequency of enrichment of top 20 enriched genes in pathways. (**E**) Bar chart of top 30 regulated pathways in mucosa of SCI-inosine versus SCI-vehicle. (**F**) Word cloud of most frequent genes regulated and enriched in the regulated pathways. (**G**) Circos plot of top 2 pathways (based on *P* value) and enriched genes. (**H**) Bar chart visualizing the frequency of enrichment of top 20 enriched genes in pathways. (**I**) Bar chart of top 30 regulated pathways in mucosa of SCI-inosine versus Control. (**J**) Word cloud of most frequent genes regulated and enriched in the regulated pathway. (**K**) Circos plot of top 2 pathways (based on *P* value) and enriched genes. (**L**) Bar chart visualizing the frequency of enrichment of top 20 enriched genes in pathways.

The top regulated canonical pathways identified in the mucosa of SCI-vehicle versus Control included GP6 signaling, axonal guidance signaling, inhibition of matrix metalloproteases, noradrenaline, and adrenaline degradation, and several related to oxidative stress (Figure 4A). GP6 signaling regulates wound healing, and the pathway was enriched in multiple collagen-related genes as well as those linked to inflammation. The Axonal Guidance signaling pathway was enriched in genes such as *Ephb2*, *Robo2*, and *Semas* as well as regulators of ECM turnover such as *Adam* and *Mmp* family members (Figure 4B), all of which have been implicated in regulation of axon guidance during development (reviewed in ref. 32). The genes most frequently enriched among the top 20 regulated pathways in mucosa of vehicle-treated SCI rats versus controls included *Aldh1a3*, *Prkcq*, *Aldh3a1*, *Nfatc4*, *Ccnd1*, *Cdk6*, *Fcer1g*, *Mmp14*, *Mmp15*, *Mmp2*, and *Mmp23b*. Enrichment of these genes signify their potential importance in the biological processes underlying the observed pathway alterations, highlighting their potential roles as key players in the mucosa in response to SCI (Figure 4, C and D, Supplemental Figure 10A, and Supplemental Figure 11A).

Analysis of data from mucosa of SCI-inosine versus SCI-vehicle identified ILK signaling, axonal guidance signaling, p38 MAPK signaling, inhibition of matrix metalloproteases, and semaphorin signaling in neurons (Figure 4E) as the most significantly enriched. Genes enriched in ILK signaling included those encoding regulators of the cytoskeleton and contractility (*Actg1*, *Actn1*, *Cfl1*, *Fn1*, *Itgb8*, *Myh6*, *Rhob*, *Rhoc*, *Rhoj*, *Vcl*) as well as transcription factors (*Atf4*, *Jun*) (Figure 4, F and G). The genes most frequently represented within the top 20 regulated pathways in the mucosa of inosine-treated SCI rats compared with vehicle-treated animals included *Jun*, *Cd247*, *H2-Eb2*, *Hla-A*, *Hla-Dra*, *Atf4*, *Ptgs2*, *Rhob*, *Rhoc*, and *Rhoj* (Figure 4H, Supplemental Figure 10B, and Supplemental Figure 11B). Pathways no longer enriched in mucosa following inosine treatment included those related to oxidative stress and p38 MAPK signaling in agreement with the known antioxidant activity of inosine (reviewed in ref. 33). While GP6 Signaling still showed some level of enrichment, this was substantially reduced in mucosa following inosine treatment. These findings suggest that processes such as wound healing and ECM turnover and neurotransmitter regulation in the mucosa are sensitive to inosine treatment following SCI.

Pathway analysis of data from mucosa of SCI-inosine versus Control identified ILK signaling, caveolar-mediated endocytosis signaling, and several pathways related to cell-cell junctions (epithelial adherens junction, tight junction) as the most significantly enriched (Figure 4I). Genes enriched in ILK Signaling included those encoding regulators of the cytoskeleton and contractility (*Actg1*, *Actn1*, *Cfl1*, *Fn1*, *Itgb8*), whereas genes enriched in caveolar-mediated endocytosis signaling included *Flna*, *Flnc* and those encoding integrin subunits (*Itga1*, *Itga3*, *Itgb6*, *Itgb8*) (Figure 4, J and K). The genes most frequently represented within the top 20 regulated pathways in the mucosa of inosine-treated SCI rats compared with controls included those encoding cytoskeletal proteins and their regulators (*Acta1*, *Acta2*, *Actg2*, *Myl9*, *Limk1*), mediators of signal transduction (*Mapk13*, *Rras2*, *Adcy1*, *Adcy5*, *Prkcb*), and transcription factors implicated in bladder remodeling (*Jun*, *Fos*) (34, 35) (Figure 4, J and L, Supplemental Figure 10C, and Supplemental Figure 11C).

**Figure 5:**
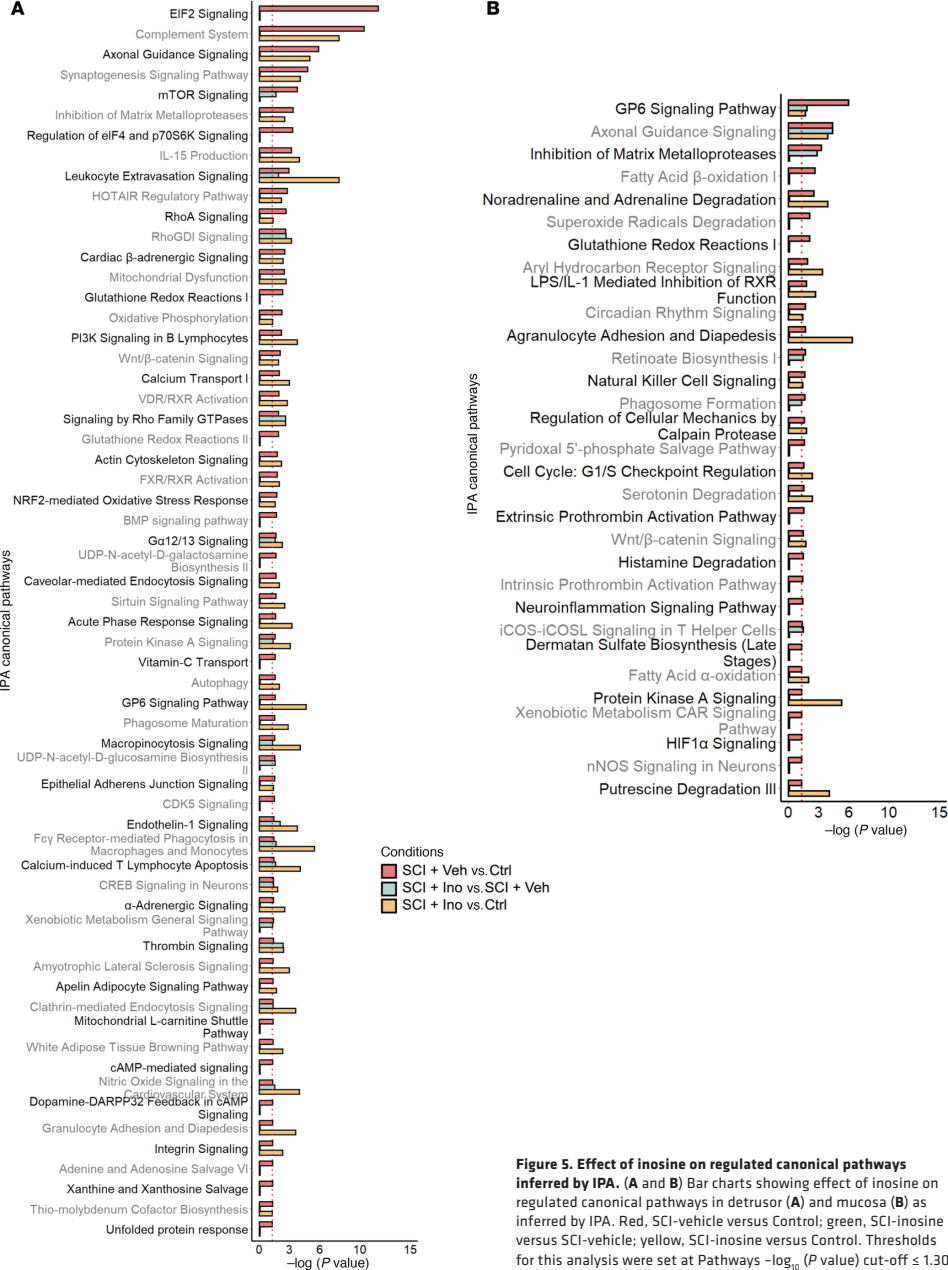
Effect of inosine on regulated canonical pathways inferred by IPA. (**A** and **B**) Bar charts showing effect of inosine on regulated canonical pathways in detrusor (**A**) and mucosa (**B**) as inferred by IPA. Red, SCI-vehicle versus Control; green, SCI-inosine versus SCI-vehicle; yellow, SCI-inosine versus Control. Thresholds for this analysis were set at Pathways –log_10_ (*P* value) cut-off ≤ 1.30 and pathway *z* score cut-off ≥ |1|.

To better visualize the changes in canonical pathways with injury and their sensitivity to inosine treatment, we combined the 3 comparisons (SCI-vehicle versus Control; SCI-inosine versus SCI-vehicle and SCI-inosine versus Control) on a single graph for each tissue (Figure 5). In detrusor, the pathways most significantly perturbed by injury and sensitive to inosine treatment included those related to complement system, EIF2 signaling, axonal guidance and synaptogenesis, and GP6 signaling (Figure 5A). In mucosa, the pathways most significantly perturbed by injury and sensitive to inosine treatment included GP6 signaling, noradrenaline and adrenaline degradation, and those related to oxidative stress (fatty acid oxidation, glutathione redox reactions, and others) (Figure 5B). Collectively, these pathways emphasize the impact of SCI on processes related to wound healing and tissue remodelling (EIF2 and GP6 signaling) as well as innervation and neurotransmission (axonal guidance, synaptogenesis, noradrenaline and adrenaline degradation and complement system) in the bladder wall. This analysis also highlights the distinct responses of detrusor and mucosa to SCI and their sensitivity to inosine treatment.

### Master regulator analysis implicates an inosine-sensitive DNA damage response following SCI.

Following the identification of differentially regulated genes and pathways associated with SCI, we next aimed to identify master regulators that were predicted to drive the observed changes in gene expression with injury and to predict their potential modulators. To accomplish this, we used upstream regulator (UR) and causal network (CN) approaches in IPA (36) to predict the most significant URs and expose causal relationships associated with genes including regulators that are directly and indirectly connected to targets in our dataset.

UR analysis of data from vehicle-treated SCI detrusor versus control predicted TP53 and MYC as the transcriptional regulators with the most significant *P* values of overlap and activation *z* scores > 2. CN analysis, which provided additional insights into putative master regulators, predicted TRRAP as the most significant transcriptional regulator (*z* score, 2.838; *P* value of overlap, 1.39 × 10^–34^) from DEGs from vehicle-treated SCI detrusor versus control (data not shown). TRRAP encodes a member of the PIKK family of PI3K-related kinases — including DNA-PKcs, ATM, and ATR — that senses and responds to DNA damage (37). TRRAP is known to interact with TIP60, which was predicted as significantly activated in our data (*z* score, 3.619; *P* value of overlap, 6.3 × 10^–30^) and has been implicated in DNA repair (38). The identification of regulators of DNA damage is consistent with findings from the time course analysis.

**Figure 6:**
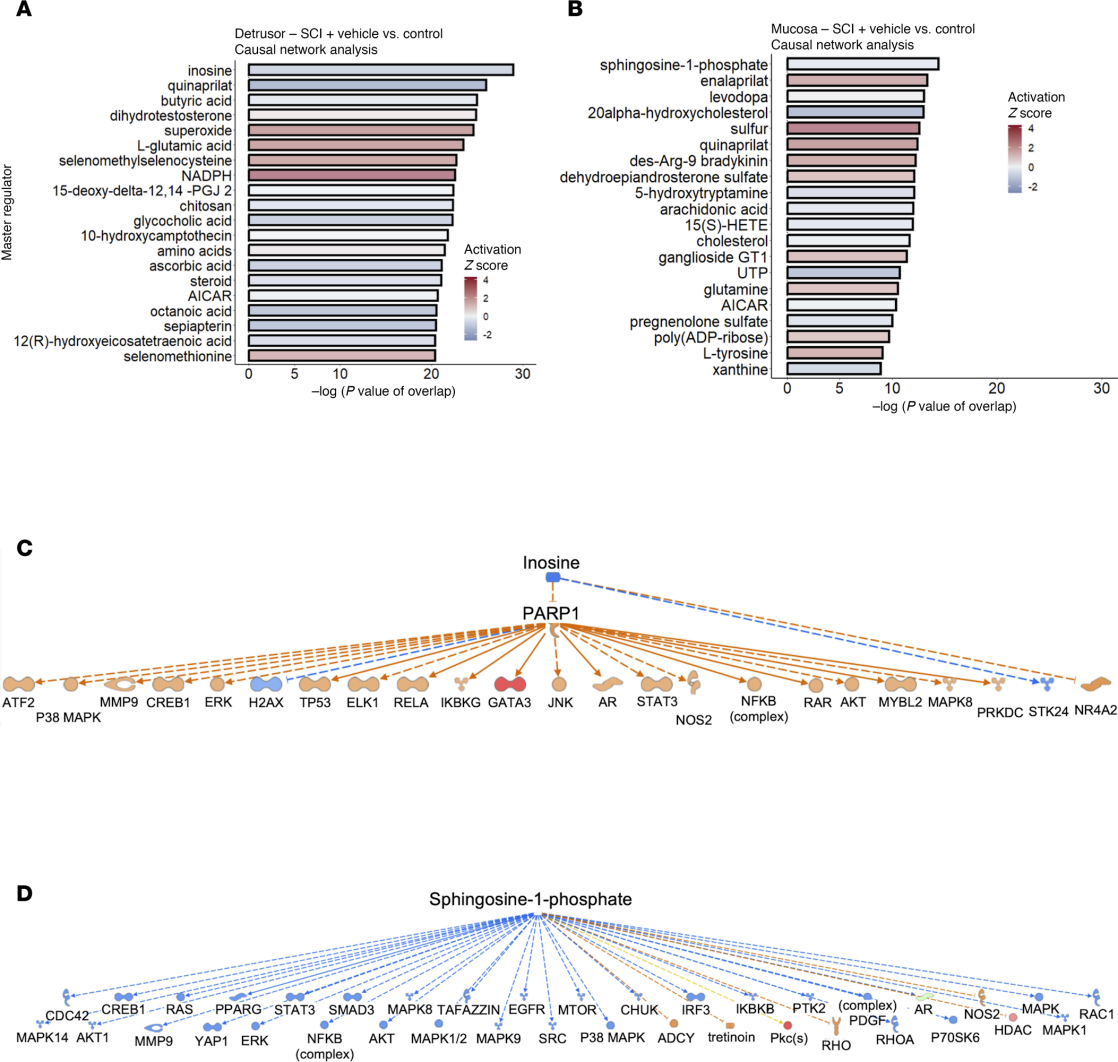
Causal network analysis of transcriptomic data from detrusor and mucosa. (**A**) Bar chart showing causal network analysis in detrusor of SCI rats treated with vehicle compared with uninjured controls (SCI-vehicle versus Control [8 weeks]) highlighting inosine as the most significantly downregulated endogenous chemical. (**B**) Bar chart showing causal network analysis in mucosa of SCI rats treated with vehicle compared with uninjured controls (SCI-vehicle versus Control [8 weeks]) highlighting sphingosine-1-phosphate as the most significantly downregulated endogenous chemical. Red bars indicate endogenous chemicals with a positive *z* score that are predicted active. Blue bars indicate endogenous chemicals with a negative *z* score that are predicted to be inactive. (**C**) Predicted network of top endogenous chemicals and downstream targets (23) in the detrusor of SCI-vehicle rats versus Control. (**D**) Predicted network of top endogenous chemical and the downstream targets (39) in the mucosa of SCI-vehicle rats versus Control.

We also used CN analysis to identify potential modulators of the observed gene expression changes. Since our goal was to identify agents that could form the basis of treatments, we focused on endogenous chemicals, with the rationale that these would tend to be well tolerated in vivo (Figure 6). Analysis of vehicle-treated SCI detrusor versus control predicted inosine as the most significant endogenous chemical (*z* score = –1.53, *P* value of overlap = 1.14 × 10^–29^) (Figure 6A). The negative *z* score indicated that inosine was predicted to inhibit the corresponding networks. By interrogating the putative targets, we inferred that inosine lies upstream of poly-ADP ribose phosphorylase-1 (PARP1) within the detrusor following SCI. The molecules downstream of PARP1 were found to be activated, leading us to conclude that PARP1 is activated in the bladder following SCI (Figure 6C). Predicted targets of PARP1 included the stress-activated kinases *Jnk* and *p38 Mapk*, the DNA damage–associated kinase *Prkdc* (DNA-PK), NF-κB family members (*Ikbkg*, *Nfkb* and *Rela*), and *Tp53*, among others (Figure 6C), further emphasizing the link to DNA damage. Interestingly, CN analysis also predicted inosine to activate a distinct downstream network (*z* score = 4.264; *P* value of overlap = 5.37 × 10^–16^), that was nonoverlapping with the PARP1-regulated network (data not shown). The predicted targets in this inosine-activated network include complement factors, the annexins *Anxa1* and *Anxa3*, and genes encoding actin-binding proteins such as *Arpc1b* and *Lcp1*. The prediction of both inosine-inhibited and inosine-activated networks by CN analysis suggests that SCI itself modulates inosine metabolism in the bladder.

UR analysis of vehicle-treated SCI mucosa versus control predicted *Myc*, *Spdef*, *Smad7*, and *Ppargc1a* as transcriptional regulators with the most significant *P* values of overlap and activation *z* scores > 2 (data not shown). CN analysis of DEGs from vehicle-treated SCI mucosa versus control predicted one of the most significant regulators in mucosa to be the *Ddb1/Cul4/Rbx1* complex, which is known to regulate DNA damage response signaling (39). Among the endogenous chemicals, sphingosine-1-phosphate (S1P) emerged as the most significantly altered (*z* score = –0.659; *P* value of overlap = 3.97 × 10^–15^) (Figure 6B). Sphingosine kinase 1, which generates S1P from sphingosine, was also predicted to be inhibited (*z* score = –2.157; *P* value of overlap = 1.9 × 10^–15^) in agreement with the predicted decrease in S1P. Consistent with findings in detrusor, S1P has been linked in prior studies to modulate the response to DNA damage in neurons (40). Bioinformatics analysis was used to predict functional relationships between S1P and downstream genes, suggesting that S1P inhibits *Adcy*, *Hdac*, and *Mtor*. In addition, S1P was predicted to be involved in the regulation of multiple molecules, including *Akt1*, *Chuk*, *Creb1*, *Egfr*, and *Erk* (Figure 6D).

The UR analysis of detrusor and mucosa tissues from SCI rats without and with inosine treatment revealed overlapping themes with the earlier time-course analysis (2, 8, and 16 weeks after SCI). Both studies highlighted the central role of DNA damage response signaling, ECM remodeling, and inflammation in SCI-associated alterations within the bladder wall. Notably, *Tp53* emerged as a key regulator in both datasets, emphasizing its role in the response to DNA damage. Similarly, *Myc* was predicted as a significant transcriptional regulator across mucosa and detrusor, underscoring its contribution to cell proliferation and stress responses. DNA damage response pathways were further implicated through the activation of *Trrap* and *Tip60* in the detrusor. Moreover, common molecular mediators, such as ERK, Akt, and MAPKs, were found to play overlapping roles in cellular signaling, inflammation, and tissue remodeling. Additionally, contributors to ECM remodeling (e.g., Col1a1 and Cxcl12) were prominent in both datasets, reinforcing the central theme of dysregulated wound healing, fibrosis, and inflammation following SCI.

### Inosine attenuates oxidative stress and DNA damage in the bladder following SCI.

**Figure 7:**
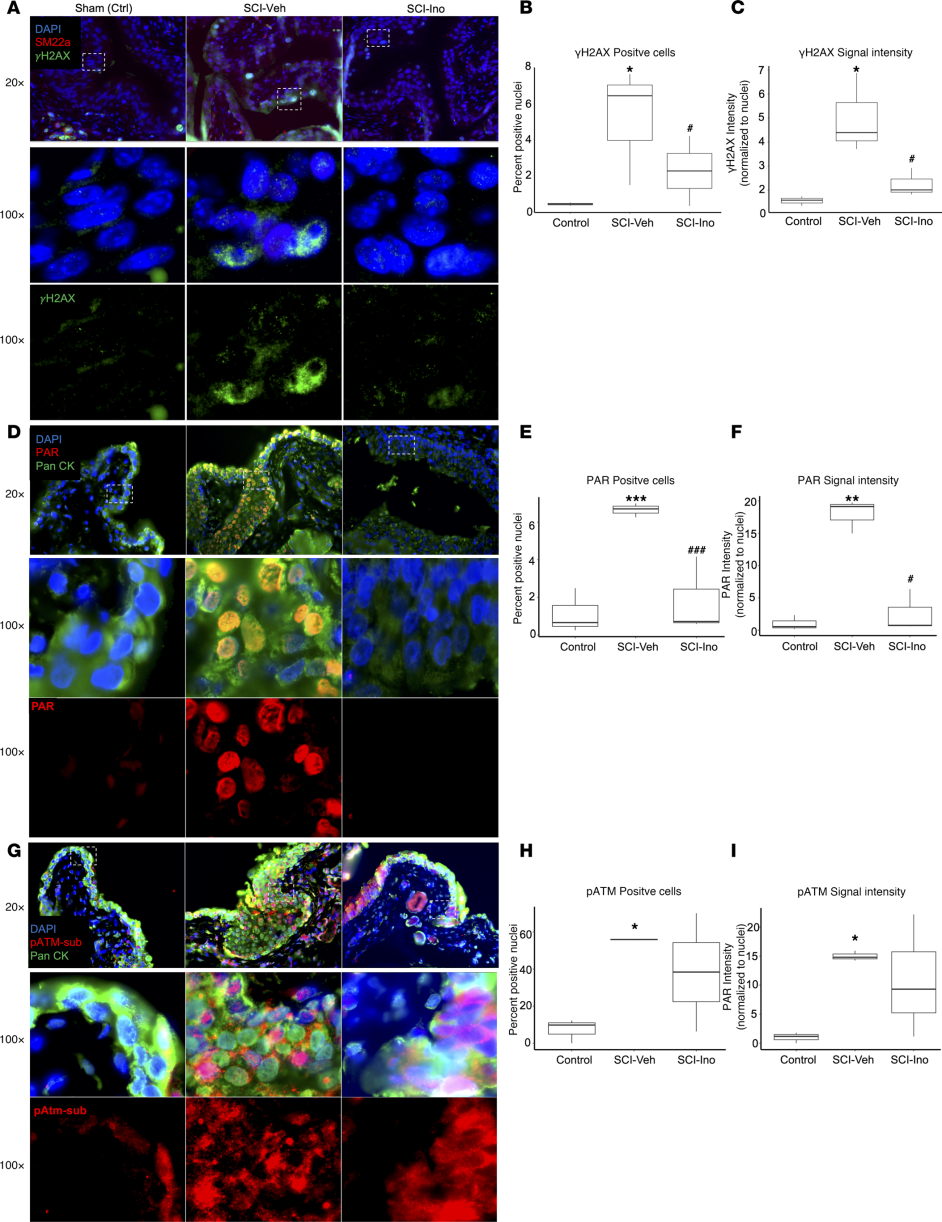
Immunostaining for causal network and pathway validation. (**A**, **D**, and **G**) Bladder sections from SCI and Control rats were stained for phosphorylated histone H2AX (γH2AX) (green) and SM22a (red) (**A**), poly/mono ADP Ribosylation (PAR) (red) and pan cytokeratin (Pan-Ck) (green) (**D**), or pATM-substrates (pATM-sub) (red) and pan-cytokeratin (Pan-Ck) (green) (**G**). Nuclei were visualized with DAPI (magnification, 20×). White dashed rectangles indicate regions of tissue visualized at 100×. Images were analyzed with ImageJ-based macro outlined in Supplemental Figure 13 that quantified nuclear staining for the markers of interest. (**B**, **E**, and **H**) Quantification of percentage of positive nuclei for each DNA damage–associated marker. (**C**, **F**, and **I**) Quantification of the nuclear signal intensity for each DNA damage–associated marker. A total of 5–15 fields of view were captured at 20× with > 5,000 nuclei represented for each of 3 biological replicates per condition. Significance was determined by 1-way ANOVA followed by Tukey’s multiple comparisons test. Adjusted *P* values were used to report the significance of the differences. *P < 0.05, **P < 0.01, versus control. ^#^*P* < 0.05, #^#^*P* < 0.01, versus SCI.

**Figure 8:**
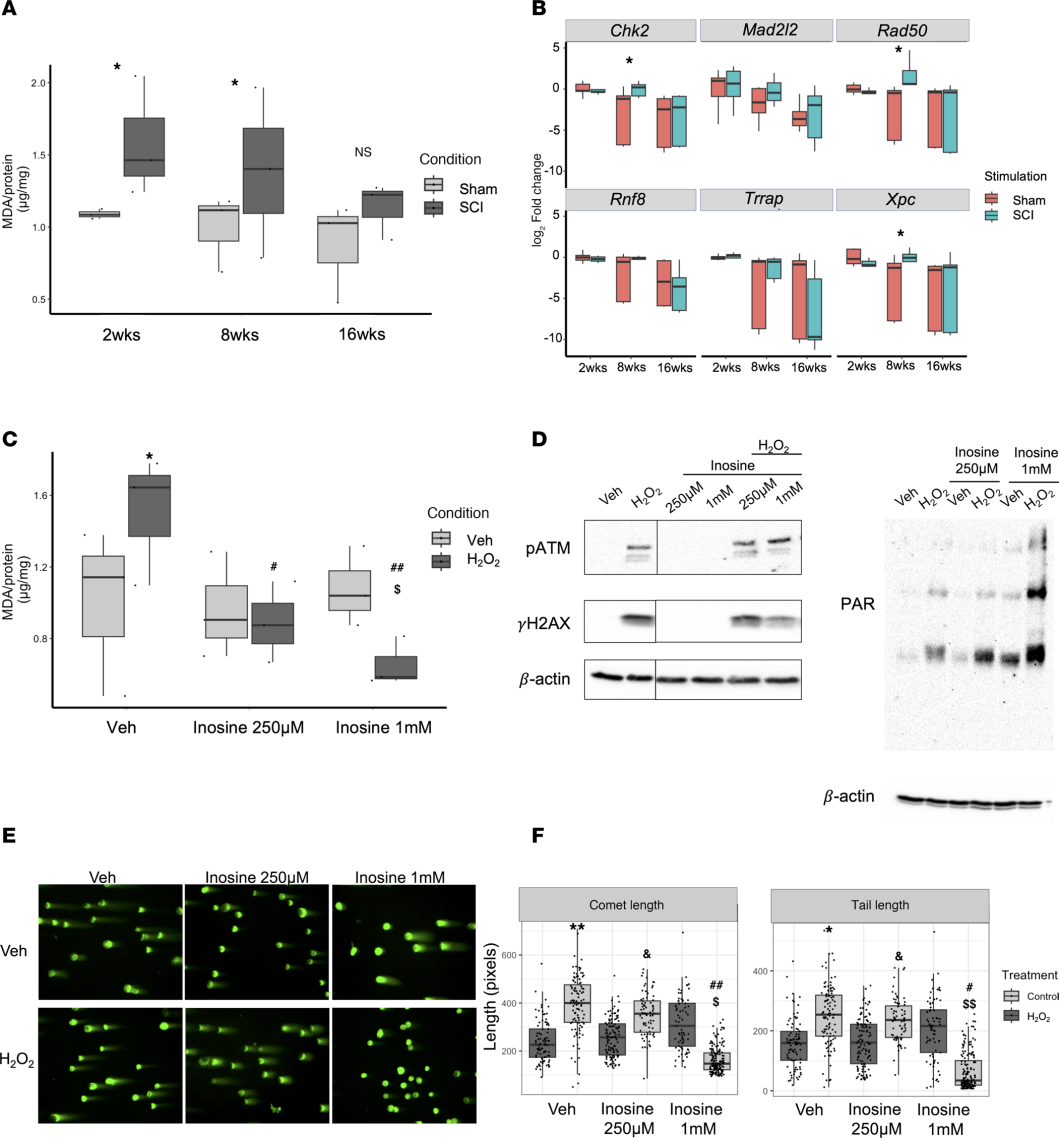
In vitro validation of oxidative DNA damage and its sensitivity to inosine. (**A**) Protein lysates of bladders harvested from rats at 2, 8, or 16 weeks following SCI or age-matched controls were assessed for malondialdehyde (MDA), a measure of lipid peroxidation (*n* = 3 biological replicates). (**B**) Expression of DNA damage–related genes in bladder tissue collected at the indicated times was assessed via qPCR (*n* = 4–6 biological replicates). (**C**) MDA was assessed in rat bladder fibroblasts treated ± H_2_O_2_ or PBS ± indicated doses of inosine (*n* = 3 biological replicates). (**D**) Immunoblot analysis of DNA damage–associated markers including phospho-ATM serine/threonine kinase (pATM), γH2AX, and Poly/Mono ADP Ribosylation (PAR) in rat bladder fibroblasts treated ± H_2_O_2_ or PBS ± indicated doses of inosine. (**E**) DNA damage was assessed by comet assay using rat bladder fibroblasts stimulated with 50 μM H_2_O_2_ ± indicated doses of inosine. Magnification, 20×. (**F**) Comet assay quantification, with 100–200 nuclei assessed for each condition. Data are representative of 3 independent trials. Significance was determined by 1-way ANOVA followed by Tukey’s multiple comparisons test. Adjusted *P* values were used to report the significance of the differences. *P < 0.05, **P < 0.01, versus control (Veh). ^#^*P* < 0.05, ^##^*P* < 0.01, versus H_2_O_2_ 50μM. ^$^*P* < 0.05, ^$$^*P* < 0.01, versus 1 mM inosine. ^&^*P* < 0.05, versus 250 μM inosine.

To test the prediction that the DNA damage response is enriched in the bladder wall following SCI, rat bladder tissues were stained for phospho-ATM substrates, indicative of ATM activation; γH2AX, the target of activated ATM; and poly-ADP-ribose (PAR), a marker of PARP activity. The extent and intensity of signal for γH2AX (Figure 7, A–C), PAR (Figure 7, D–F), and pATM substrate (Figure 7, G–I) were induced significantly by SCI compared with control (*P* < 0.05). Notably, a similar induction of PAR and γH2AX signal was observed in bladder sections of patients with neurogenic bladder compared with controls (Supplemental Figure 12, A–F). In contrast, signals for γH2AX and PAR were significantly attenuated in tissues from SCI rats treated with inosine (*P* < 0.05). To validate the prediction from canonical pathway analysis that oxidative stress–associated pathways were enriched following injury, we assessed the level of malonyldialdehyde (MDA), a marker of lipid peroxidation, in the bladder. We verified a time-dependent increase in oxidative stress in the bladder wall (Figure 8A), as shown by increased MDA levels at 2 and 8 weeks after SCI versus controls. Expression of DNA damage–related genes including *Chk2*, *Rad50*, and *Xpc* was also increased at 8 weeks after SCI (*P* < 0.05) (Figure 8B). We further explored the relationship between oxidative stress and DNA damage in vitro by measurement of lipid peroxidation, DNA damage–related genes and proteins, and DNA damage directly via comet assay in rat bladder cells exposed to H_2_O_2_ to evoke oxidative stress (Supplemental Figure 14, A–E). Increased signal for MDA and DNA damage, as shown by significant increases in comet length and tail length, were evident in H_2_O_2_-treated cells, both of which were attenuated with inosine (*P* < 0.05) (Figure 8, C, E, and F). Moreover, changes in the comet assay were paralleled by changes in biochemical markers of DNA damage including pATM, γH2AX, and PAR (Figure 8D). Taken together, these observations demonstrate that SCI is associated with an oxidative stress–induced DNA damage response that is attenuated with inosine treatment.

### Proteomics analysis reveals enrichment of EIF2, oxidative stress, and neuronal signaling after SCI.

**Figure 9:**
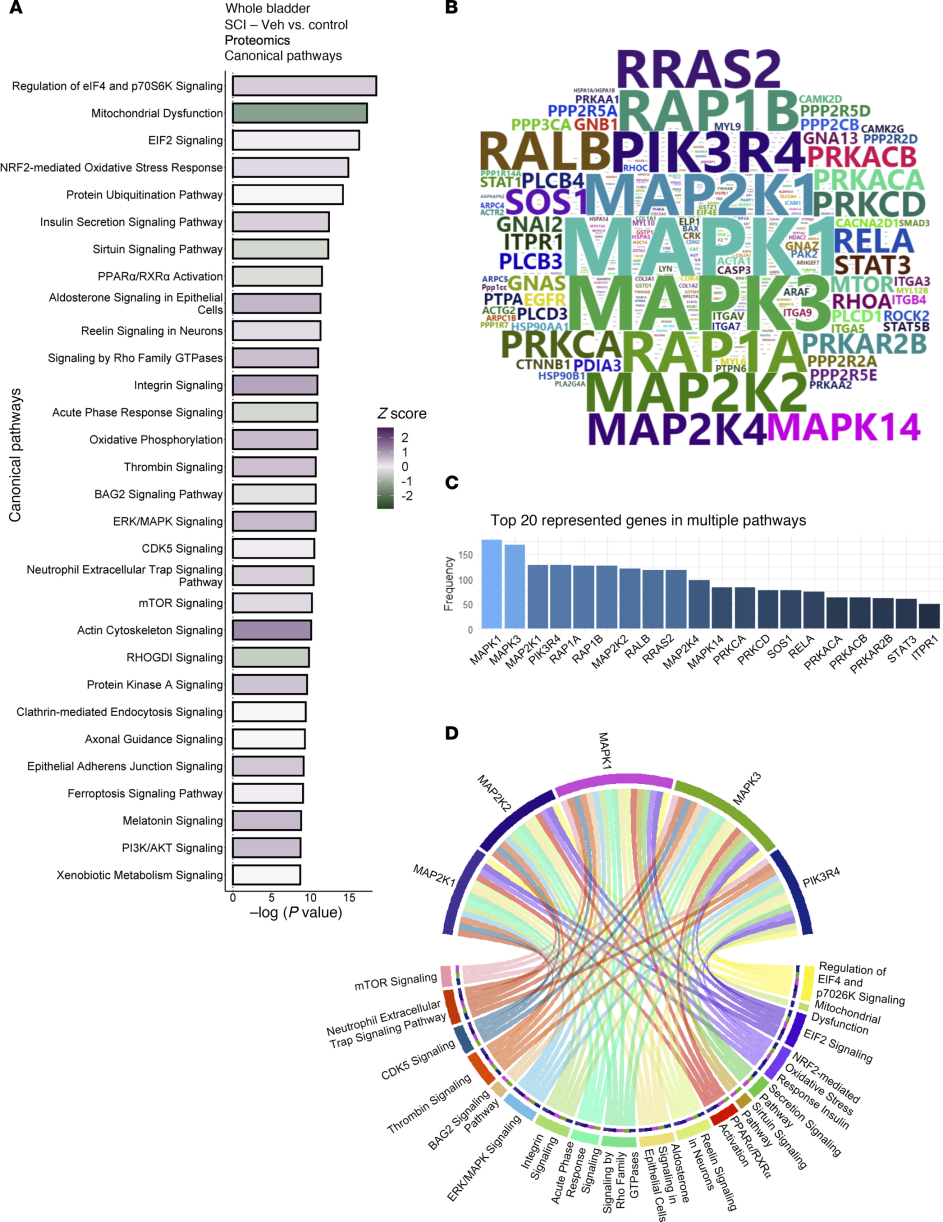
Regulated canonical pathways inferred by IPA of proteomics data. (**A**) Bar chart of top 30 regulated pathways in full-thickness bladder tissue at 8 weeks after SCI compared with age-matched controls. (**B**) Word cloud of most frequent proteins regulated and enriched in the regulated pathways. (**C**) Bar chart showing the frequency of top 20 most recurrent protein enriched in pathways. (**D**) Circos plot of top 5 most recurrent enriched proteins in the regulated pathways and the corresponding regulated pathways.

To augment our findings from the transcriptomics analysis, we also performed quantitative proteomics analysis on full-thickness bladder samples obtained from the time-course experiment. Analysis of differentially expressed proteins using IPA revealed 296 canonical pathways that were significantly regulated at 8 weeks following SCI compared with control. The top enriched pathways included those linked to protein synthesis such as regulation of eIF4 and p70S6K signaling, EIF2 and mTOR, as well as those related to oxidative stress, including NRF2-mediated oxidative stress response, oxidative phosphorylation and mitochondrial dysfunction (Figure 9A). Enrichment for pathways driving protein synthesis is consistent with the profound increase in bladder/body weight ratio observed following SCI, whereas those associated with oxidative stress reflect our demonstration of increased MDA levels in the bladder wall after injury. Consistent with the neurogenic origin of injury, pathways related to neuronal function were also enriched in the proteomics data, including reelin signaling in neurons, which is closely tied to the regulation of neurogenesis, neuronal migration, and synaptic plasticity (41); Axonal Guidance Signaling implicated in regulating neuronal guidance during development; and CDK5 Signaling, which plays a pivotal role in orchestrating neuronal cell cycle progression, synaptic plasticity, and neuronal development (42). Interestingly, CDK5 has been implicated in the response to DNA damage (43) consistent with the increased ATM activation, γH2AX, and PAR staining in the bladder following SCI. Some of these pathways including EIF2 signaling, mitochondrial dysfunction, NRF2-mediated oxidative stress response and sirtuin signaling were also enriched at 2 and 16 weeks after injury (data not shown), highlighting their relevance to both acute and chronic neurogenic bladder pathology. Additional pathways enriched across the time-course included those related to integrin, actin cytoskeleton and RhoGDI signaling, reflecting ongoing cytoskeletal remodeling and altered contractility following injury, as well as sirtuin signaling pathway and PPARα/RXRα activation, suggesting alterations in inflammation and metabolism following SCI.

Assessment of the top proteins regulated in multiple pathways revealed significant enrichment of multiple MAPK family members at all time points within the regulated pathways, including MAPK1, MAPK3, MAP2K1, MAP2K2, MAP2K4, and MAPK14 (Figure 9, B and C), implicating activation of MAPK signaling in response to SCI. In addition to identification of the ERK/MAPK signaling pathway among the top regulated canonical pathways, MAPK family members were also highly represented in a majority of the top 20 regulated pathways including regulation of eIF4 and p70S6K signaling, NRF2-mediated oxidative stress response, reelin signaling in neurons, signaling by Rho family GTPases, and mTOR signaling (Figure 9D), among others.

To gain a greater understanding of pathway regulation in the context of SCI, we compared the significantly regulated pathways identified from proteomics analysis of full-thickness bladder samples with those identified from transcriptomics analysis of separated detrusor and mucosa from bladders at 8 weeks following SCI, and we identified considerable overlap between them (Supplemental Figure 15A). Ten pathways were common among all 3 datasets, including axonal guidance signaling, GP6 signaling, and glutathione redox reactions. Furthermore, 15 pathways were matched exclusively between those identified by proteomics of whole bladder and those enriched in the mucosa, as determined by RNA sequencing. These included HIF1α signaling, superoxide radicals degradation, fatty acid β-oxidation and pathways related to inflammation and immune cell function. In the detrusor, 49 pathways exhibited exclusive intersection with those identified by proteomics of the whole bladder and those identified by RNA-Seq. Taken together, this integrated analysis identified important regulators of the bladder tissue response to SCI as well as potential therapeutic targets but also uncovered distinct responses within the detrusor and mucosa. These latter differences, together with the differing sensitivity of detrusor and mucosa to inosine, warrant further investigation, as they are likely to reveal cell type–selective functions in the context of injury.

## Discussion

In this study, we described temporal changes as well as the effect of inosine treatment on the molecular landscape of the bladder following SCI in rats, and applied a multi-omics approach to obtain insights into both neurogenic bladder pathobiology and mechanisms of inosine action. In addition to comprehensive profiling at both the transcriptomic and proteomic level over time, this study provides the following key findings. We demonstrate DNA damage response signaling in neurogenic bladder and its attenuation with inosine treatment. We also observed similar increases in γH2AX and PAR in human neurogenic bladder specimens, highlighting the potential translational relevance of this study. Integrated transcriptomic and proteomic analyses highlighted the enrichment of canonical pathways for cell stress and wound healing following SCI, including EIF2 signaling, GP6 signaling, mitochondrial dysfunction, and oxidative stress. Together, these findings highlight key molecular features of the tissue landscape following injury and identify potential targets for therapeutic intervention.

Oxidative stress is known to arise early in the bladder after SCI. Loss of normal innervation to both the bladder and external urethral sphincter results in detrusor-sphincter dyssynergia, with the ensuing functional obstruction leading to increased pressure within the bladder during filling, thereby altering blood flow and resulting in transient ischemia/hypoxia and oxidative stress (44, 45). Published reports have described increases in reactive oxygen species (ROS) in urothelial cells as early as 3 days after injury (46). In support of a causative relationship between oxidative stress and DNA damage following SCI, we now show an increase in lipid peroxidation in the bladder with injury that coincides with the presence of DNA damage markers. Moreover, DNA damage is evoked in rat bladder cells exposed to H_2_O_2_ and paralleled by increases in DNA damage markers. These observations are consistent with reports showing that persistent DNA damage can in turn evoke oxidative stress (47), suggesting the existence of a feed-forward loop that drives pathologic changes in the bladder in the setting of ongoing obstruction. Of particular interest to us was the prevention of DNA damage in tissues of inosine-treated animals and in cells exposed to H_2_O_2_ that were cotreated with inosine. Inosine is a known antioxidant, an activity related in part to its metabolism to urate, a scavenger of reactive oxygen and reactive nitrogen species (reviewed in ref. 14). Consistent with this, one of the genes identified as inosine-sensitive in both detrusor and mucosa was *Coq7* (coenzyme Q7), which encodes the mitochondrial enzyme CLK-1, implicated in cellular respiration. *Coq7* was induced in the bladder in response to SCI but was maintained at control levels in tissues from inosine-treated animals. In previous studies, decreased CLK-1 levels were associated with attenuation of mitochondrial oxidative stress in models of accelerated aging (48). Moreover, the neurodegeneration-inhibiting agent clioquinol was found to act through inhibition of CLK-1 in cell and animal models (49). CLK-1–mediated signaling has also been shown to regulate stress responses in mitochondria, including the metabolism of ROS and the unfolded protein response (50), both of which have been implicated in bladder dysfunction of both neurogenic and nonneurogenic origin (46, 51, 52). Our data, showing enrichment of pathways related to EIF2 signaling, oxidative stress, and mitochondrial dysfunction, extends these prior observations and provides additional insights into mechanisms of inosine action.

Consistent with the enrichment of oxidative stress–associated pathways and DNA damage, SCI was also associated with a net increase in PAR signal in the bladder, suggesting activation of PARP, itself attenuated with inosine treatment. Purines, including inosine, have been shown previously to inhibit PARP1 activity under cell-free conditions and in cultured macrophages exposed to peroxynitrite (53). In that study, Virag and Szabo showed that the inosine metabolite, hypoxanthine, was more potent at inhibiting PARP than inosine and conferred greater cytoprotection of macrophages exposed to oxidative stress compared with inosine (53). These observations are in contrast to findings in the lower urinary tract where hypoxanthine has been implicated in damage to the bladder with age (54, 55). Inhibition of purine nucleoside phosphorylase (PNPase), the enzyme that converts inosine to hypoxanthine, in aged rats, was associated with improvement in bladder morphology and function (54). These included mitochondrial damage, oxidative stress, and voiding dysfunction, all of which are also evident in neurogenic bladder following traumatic SCI in spite of the distinct etiology. Analysis of our proteomics data revealed a significant increase in levels of PNPase in bladder tissue from rats with SCI (Supplemental Figure 15B), consistent with the possibility of increased inosine metabolism to hypoxanthine and its associated deleterious effect on tissue following injury. Taken together, these findings support the concept that bladder homeostasis is influenced by the balance between protective and toxic purines as exemplified by inosine and hypoxanthine, respectively (54). Furthermore, they reinforce the idea that increasing inosine levels, whether by exogenous administration as described by us (13) or by inhibition of inosine metabolism to hypoxanthine (54), represents an effective therapeutic approach to prevent the adverse consequences of injury to the bladder.

In addition to functional modulation of downstream effectors by ADP-ribosylation, PARP uses NAD^+^ as the substrate for generation of ADP-ribosyl moieties. As a result, PARP activation leads to NAD^+^ depletion, thereby exacerbating cellular stress (56), whereas PARP inhibition prevents NAD^+^ depletion and its deleterious consequences. Restoration of NAD^+^ levels either through direct supplementation with NAD^+^ or by administration of precursors such as nicotinamide riboside (NR) has shown benefit in aging and neurodegenerative models (reviewed in ref. 57). Metcalfe and colleagues showed that administration of NR led to improved recovery of motor control in rats with midthoracic SCI, by preserving neurons within the spinal cord itself (58). Canonical pathways identified in our analysis include several that are sensitive to NAD^+^ modulation. These include EIF2 signaling, indicative of ER stress, activation of which has been linked to NAD^+^ depletion, oxidative stress–related pathways including mitochondrial dysfunction known to be exacerbated by NAD^+^ depletion, and immune cell signaling (59, 60). Notably, inosine administration has been shown to preserve NAD^+^ levels (61) and to mediate antiinflammatory effects of elevated NAD^+^ (62) in settings of systemic inflammation. The marked effect of inosine administration on DNA damage response endpoints and cell stress pathways observed in our model suggests that inosine may act, at least in part, through the preservation of NAD^+^ levels.

A major finding in this study is the attenuation of DNA damage response signaling, as well as pathways related to cell stress (EIF2 signaling, oxidative stress, GP6 signaling, mitochondrial dysfunction) in tissues from inosine-treated versus vehicle-treated animals. Although we demonstrate direct effects of inosine on oxidative DNA damage in bladder cells in vitro, suggesting that inosine can act locally, we cannot exclude the possibility that the transcriptomic and proteomic changes captured in bladder tissues in vivo reflect inosine activity at sites other than the bladder, such as the spinal cord, that in turn improve bladder health. In support of this possibility, PARP inhibition was shown to reduce neuronal apoptosis within the spinal cord itself and promote recovery of hindlimb function, albeit acutely, in rodent models of SCI (63–66). Distinguishing between local effects of inosine in the bladder versus distant sites is a focus of ongoing studies.

A major strength of this study is the integrated analysis of transcriptomics and proteomics data, which identified several pathways that were regulated in a concordant manner following SCI. These included EIF2 signaling, NRF2-mediated oxidative stress response, RhoA signaling, actin cytoskeleton signaling, GP6 signaling, and protein kinase AsSignaling, a number of which were attenuated in tissues from inosine-treated animals. Alterations in EIF2 signaling are central to the integrated stress response that mediates compensatory changes in protein synthesis in cells and tissues exposed to injury thereby enabling recovery and repair. However, although beneficial early after injury, sustained activation of the ISR has been associated with damaging consequences in a variety of disease settings, leading to a search for strategies to inhibit EIF2 signaling to prevent the ISR from becoming maladaptive (reviewed in ref. 67). Notably, ribosomal proteins associated with EIF2 signaling that were identified as significantly altered in SCI-vehicle versus control samples, were attenuated in samples from inosine-treated animals, further supporting the beneficial ability of inosine to reduce the integrated stress response (Supplemental Figure 16, A and B). EIF2 signaling has been implicated in pathological changes in the brain and spinal cord neurons following injury, with eIF2α phosphorylation, central to the ISR, increased early after injury in parallel with emergence of morphological and functional deficits (68, 69). In contrast, inhibition of EIF2 signaling using the small molecule ISRIB was associated with both histological and functional improvements. Thus, inhibition of EIF2 signaling with inosine is predicted to achieve similar neuroprotective benefits in the bladder, consistent with our prior findings (13).

There are several limitations in our study. All analyses were performed in male rats to enable comparison with previous studies conducted by our group (13, 16, 18), potentially limiting applicability of the findings to both sexes. We also lack proteomics data from separated detrusor and mucosa and the response of the proteome to inosine, such that we cannot assess the concordance between transcriptome and proteome in these tissues. Nonetheless, we were encouraged by the substantial overlap in commonly regulated pathways predicted from transcriptomics and proteomics analysis, which suggested strong agreement between them.

In conclusion, we present a comprehensive analysis of the bladder following traumatic SCI in a preclinical rat model. Our integrated transcriptomic and proteomic analysis highlights key signaling pathways perturbed with SCI, including EIF2 signaling, NRF2-mediated oxidative stress response, and GP6 signaling, that reflect cellular stress responses. We also unveil the systemic effect of inosine following neurogenic injury that extends beyond attenuation of the DNA damage response to the preservation of key biological networks and pathways. This rebalancing of molecular pathways by inosine underscores the potential of systems biology approaches to investigate complex neurogenic disorders and suggests potentially new therapeutic possibilities, such as the repurposing of FDA-approved PARP inhibitors, the use of NAD^+^ supplementation, or administration of inosine for managing neurogenic bladder dysfunction. This study not only contributes to our understanding of the molecular landscape following traumatic injury, but it also opens avenues for targeted, effective treatments grounded in a systems approach.

## Methods

### Sex as a biological variable.

The animal model described in this study was performed in males to enable comparison with prior studies in our laboratory. For human tissue specimens, a mix of male and female neurogenic and control specimens was assessed. Sex as a biological variable was not considered in this study.

### Creation of SCI model in rats.

Complete spinal cord transection at T8 was performed in male Sprague Dawley rats (6–7 weeks of age, ~250 grams, Charles River Laboratories) under isoflurane anesthesia essentially as described (16). Using spinal bony and vascular landmarks, the spinal cord was exposed at the T8-T10 segment and transected at the T8 level, with post-operative care as described previously (16). Two experiments were performed: (a) Time-course: animals were maintained for 2, 8, or 16 weeks after SCI, after which animals were euthanized via CO_2_ inhalation. Full-thickness bladder tissues were harvested in ice-cold, oxygenated Kreb’s buffer (120 mM NaCl; 5.9 mM KCl; 25 mM NaHCO_3_; 1.2 mM Na_2_H_2_PO_4_; 1.2 mM MgCl • 6H_2_O; 2.5 mM CaCl_2_; 11.5 mM dextrose), weighed and either flash frozen in liquid nitrogen and stored at –80°C, or fixed in neutral-buffered formalin and processed for embedding in paraffin. (b) Inosine treatment: rats with SCI received inosine (225 mg/kg/d) or equivalent volume of vehicle (12 mM sodium bicarbonate buffer, pH 9.2) administered by i.p. injection daily for 8 weeks. At the end of the treatment period, bladders were weighed, microdissected into detrusor and mucosa and flash frozen, or formalin-fixed and processed for embedding in paraffin.

### RNA isolation, sequencing, and quantitative PCR.

Tissues were lysed in TRIzol (Thermo Fisher Scientific) using FastPrep Lysing matrix D beads (MP Biomedical). RNA was purified using miRNeasy columns (Qiagen) according to the manufacturer’s instructions. Multimodal RNA quantification and quality control was performed using NanoDrop (Thermo Fisher Scientific), Qubit RNA Broad-Range Assay kit (Thermo Fisher Scientific), and Agilent 2100 Bioanalyzer (Agilent). The ribodepletion method of library preparation for RNA-Seq was performed using the Total-RNASeq RiboErase Kit (KAPA Biosystems). All RNA samples were loaded on 2 separate 2-lane flow cells and sequenced using an Illumina HiSeq 2500 system generating 51 bp paired-end reads. Read counts were combined from the 2 flow cells to obtain total read counts for each sample. Read count data were converted from BCL to FASTQ file type using Bcl2fastq conversion software (Illumina). Reverse transcription and semiqualitative PCR (semi-qPCR) were performed as described previously (70), with gene-specific primers listed in Supplemental Table 1A.

### Differential gene expression analysis.

Differentially regulated genes in the time course experiment were calculated using edgeR R package (4.4.1). Differential gene expression analysis of count data for inosine treatment (detrusor/mucosa) was performed using the Bioconductor R package DESeq2 (1.46.0). Variance of the data was stabilized with logarithmic transformation (log_2_) of the normalized counts. Genes were considered differentially expressed if adjusted *P* <0.05. GO term identification was performed using gprofiler (http://biit.cs.ut.ee/gprofiler/) and clusterprofiler (https://bioconductor.org/packages/release/bioc/html/clusterProfiler.html). Gene pattern clustering was performed using the DEGreport tool.

### Histological and immunofluorescence staining.

Sections (5 μm) of formalin-fixed, paraffin-embedded full-thickness rat or human bladder tissue were cut and subjected to either Masson’s trichome staining or immunofluorescence staining as described previously (13, 71). Antibodies and dilutions are listed in Supplemental Table 1B and included: γH2AX (Abcam), PAR (Cell Signaling Technology), a-SMA (MilliporeSigma), SM22a (Cell Signaling Technology [CST]), phospho-ATM substrate (CST), neuropilin 2 (MilliporeSigma),and pan-cytokeratin (MilliporeSigma) antibodies. Sections were incubated with species-specific secondary antibodies conjugated to Alexa Fluor 488 or Alexa Fluor 594, with nuclei counterstained with DAPI. Signals were visualized on an Axioplan-2 microscope (Zeiss) and representative images captured using Axiovision software. Quantitative image analysis was performed using a macro developed in house, as described in Supplemental Methods and Supplemental Figure 13. A 1-way ANOVA was performed followed by a multiple pairwise comparison between the means of groups using the Tukey Honest Significant Differences. Adjusted *P* values were used to report the significance of the differences.

### Quantitative proteomics analysis.

Tandem mass tag–based (TMT-based) quantitative proteomics analysis was conducted essentially as we previously described (72) on full-thickness bladder tissues from the time course experiment. Detailed descriptions of protein extraction, TMT labeling and fractionation, and mass spectrometry methods are presented in Supplemental Methods. Briefly, the filter-aided sample preparation method (73) was applied, and tryptic peptides were labeled with 11-plex TMT reagents in parallel, merged, desalted, and dried. Each set of TMT11plex-labeled peptides was fractionated into 48 fractions by high-pH liquid chromatography, concatenated into 16 fractions, and dried. Each fraction of TMT11plex-labeled peptides was resuspended in 0.2% formic acid, and about 1 μg peptide was loaded onto a 2 cm trap column and separated by a 50 cm EASY-Spray column (Thermo Fisher Scientific) heated to 55°C, using a 3-hour gradient at a flow rate of 250 nL/min. Liquid chromatography-synchronous precursor selection-multiple stage mass spectrometry (LC-SPS-MS3) analysis was performed on an EASY-nLC 1200 connected to an Orbitrap Fusion Lumos mass spectrometer (Thermo Fisher Scientific) using minor changes to previously reported LC and MS settings, which are reported in Supplemental Methods.

The acquired RAW files were searched against the Uniprot_Rat database, released on 07/18/2018 (https://www.uniprot.org/proteomes/UP000002494) comprising canonical and isoform protein sequences (47,943 entries) with MaxQuant (v1.6.0.16) (74). Carbamidomethyl (C) was set as fixed modification, while oxidation (M), acetyl (protein N-term), and deamidation (NQ) were set as variable modifications. The mass tolerance was 20 ppm for first search peptide tolerance, 4.5 ppm for main search peptide tolerance, and 0.5 Da for MS/MS match tolerance. The quantification type was set as Reporter ion MS3, the isobaric labels were TMT11plex, and the reporter mass tolerance was 0.003 Da. A standard FDR of 1% was used to filter peptide spectrum matches as well as peptide and protein identifications (72–74). Additional details on TMT labeling and normalization are in the Supplemental Methods.

### Measurement of oxidative stress in tissues and cells.

Oxidative stress in tissues was measured using the Lipid Peroxidation (MDA) Assay Kit (RayBiotech) according to the manufacturer’s instructions. Rat bladder mesenchymal cells were isolated and propagated essentially as described (70). Cells exposed to H_2_O_2_ (50 μM final concentration) for 24 hours were included as a positive control. MDA signal was normalized to total protein determined using the MicroBCA Protein Assay reagent (Thermo Fisher Scientific) according to the manufacturer’s instructions.

### Evaluation of DNA damage markers in vitro.

Rat bladder cells were exposed to H_2_O_2_ (up to 250 μM final concentration) for 1 hour and processed for immunoblot analysis or comet assay as outlined below. In some experiments, cells were coincubated with H_2_O_2_ and inosine, also for 1 hour. For immunoblotting, lysates were prepared and analyzed according to previously described protocols (70). Primary antibodies are listed in Supplemental Table 1A. Following chemiluminescence, signal was captured by ChemiDoc imaging or on HyBlot CL Autoradiography Film (Thomas Scientific). Films were digitized using an Epson scanner and quantified with FIJI.

### Evaluation of DNA damage by comet assay.

DNA strand breaks in rat cells were measured directly using the alkaline comet assay, essentially as described (75) but with modifications. Briefly, cells were exposed to H_2_O_2_ (50 μM final concentration) with or without 250 μM or 1 mM inosine for 1 hour at 37°C. Cells were trypsinized, centrifuged for 5 minutes at 500*g* at 4°C, washed once with PBS, and resuspended in PBS. Cell suspensions were mixed with low-melt agarose (0.7%), spread on glass slides precoated with 0.8% agarose 24 hours prior, and covered with coverslips to solidify. Following lysis (2.5M NaCl, 0.1 M EDTA, 10 mM Trizma base [pH 10], 1% N laurylsarcosine, 0.5% Triton X-100, 10% DMSO final) at 4°C for 20 minutes, cells were washed with dH_2_O and equilibrated with electrophoresis buffer (300 mM sodium acetate 100 mM Tris-HCl [pH 12] at 4°C) for 45 minutes at 4°C, before electrophoresis at 0.6V per cm. Slides were washed in PBS, 2 × 5 minutes, washed with dH_2_O, and fixed with 100% ethanol for 10 minutes. Slides were air dried for 1 hour before staining with SYBR gold + antifade and imaging. Comets were analyzed using OpenComet (76), an ImageJ (NIH) plug-in. Data generated from the analysis were plotted in R.

### Statistics.

Quantitative values from bladder/body weight, staining, comet, MDA, and qPCR assays were analyzed using 1-way ANOVA followed by multiple pairwise-comparison between the means of groups using the Tukey Honest Significant Differences. Adjusted *P* values were used to report the significance of the differences, with *P* < 0.05 considered statistically significant.

### Study approval.

The animal experiments conducted in this study were performed in strict accordance with the recommendations provided in the *Guide for the Care and Use of Laboratory Animals* (National Academies Press, 2011). The experiments were approved by the Animal Care and Use Committee at Boston Children’s Hospital (protocol no. 16-08-3256R).

### Data availability.

RNA-Seq data have been deposited in the European Nucleotide Archive (accession no. PRJEB67472) and Gene Expression Omnibus (GSE294927, GSE294930). The MS proteomics data have been deposited to the ProteomeXchange Consortium via the PRIDE partner repository with the dataset identifier PXD046096. Values for all data points in graphs are provided in the Supporting Data Values file that accompanies the article.

## Author contributions

AHG curated the data, performed bioinformatics analysis, generated figures, and wrote the original draft of the manuscript with RMA; BSS conceived the study with RMA and performed surgical procedures, tissue harvest, and endpoint analyses along with HT and HA, with contributions from GL, KC, CD, MGK, VC, and MPS. RNA-Seq was performed by SP under supervision of JAM, and transcriptomics analysis was performed by AHG, MP, and JFC. Immunofluorescence staining, immunoblotting, and comet assays were performed by ABA. Mass spectrometry was performed by WY, and proteomics analysis was performed by AHG, DV, HL, MKP, JF, and RSL. RMA conceived and supervised the study and wrote the manuscript with AHG. AHG drove the study upon the departure of BSS from the Adam laboratory, whereas ABA performed much of the experimental work required to address reviewer comments upon the departure of AHG from the Adam laboratory. For these reasons all 3 share co–first authorship, with the assigned author order. Order of the co-first authors reflects the timeline and complementarity of contributions.

## Supplementary Material

Unedited blot and gel images

Supplemental materials

Supporting data values

## Version 1 05/08/2025

In-Press Preview

## Version 2 06/23/2025

Electronic publication
