# Supplementary figures and images for: Multiomics analysis unveils an inosine-sensitive DNA damage response in neurogenic bladder after spinal cord injury
Source: JCI Insight. 2025 May 8;10(12):e180275. doi: 10.1172/jci.insight.180275 (PMC12220959; doi:10.1172/jci.insight.180275)

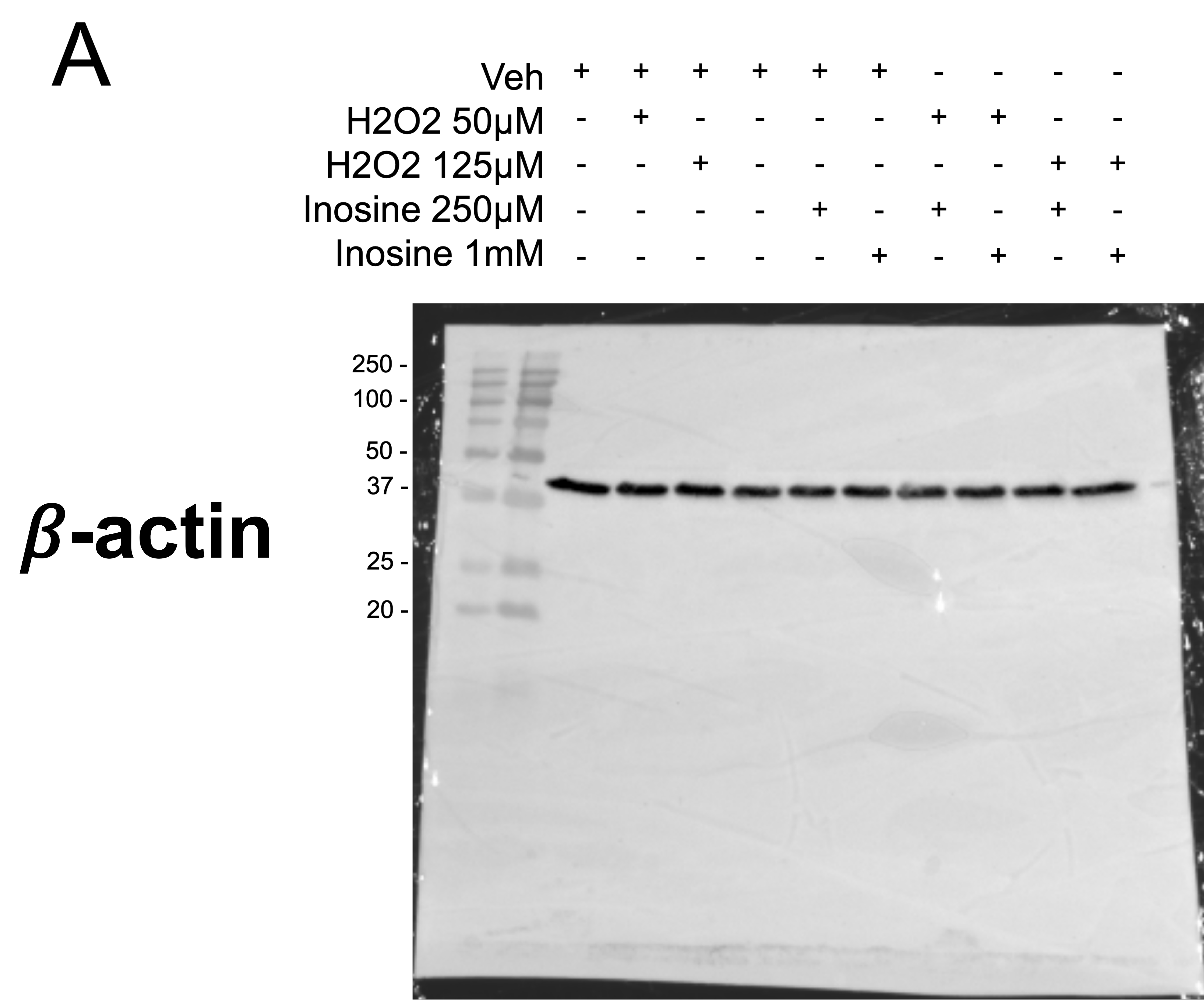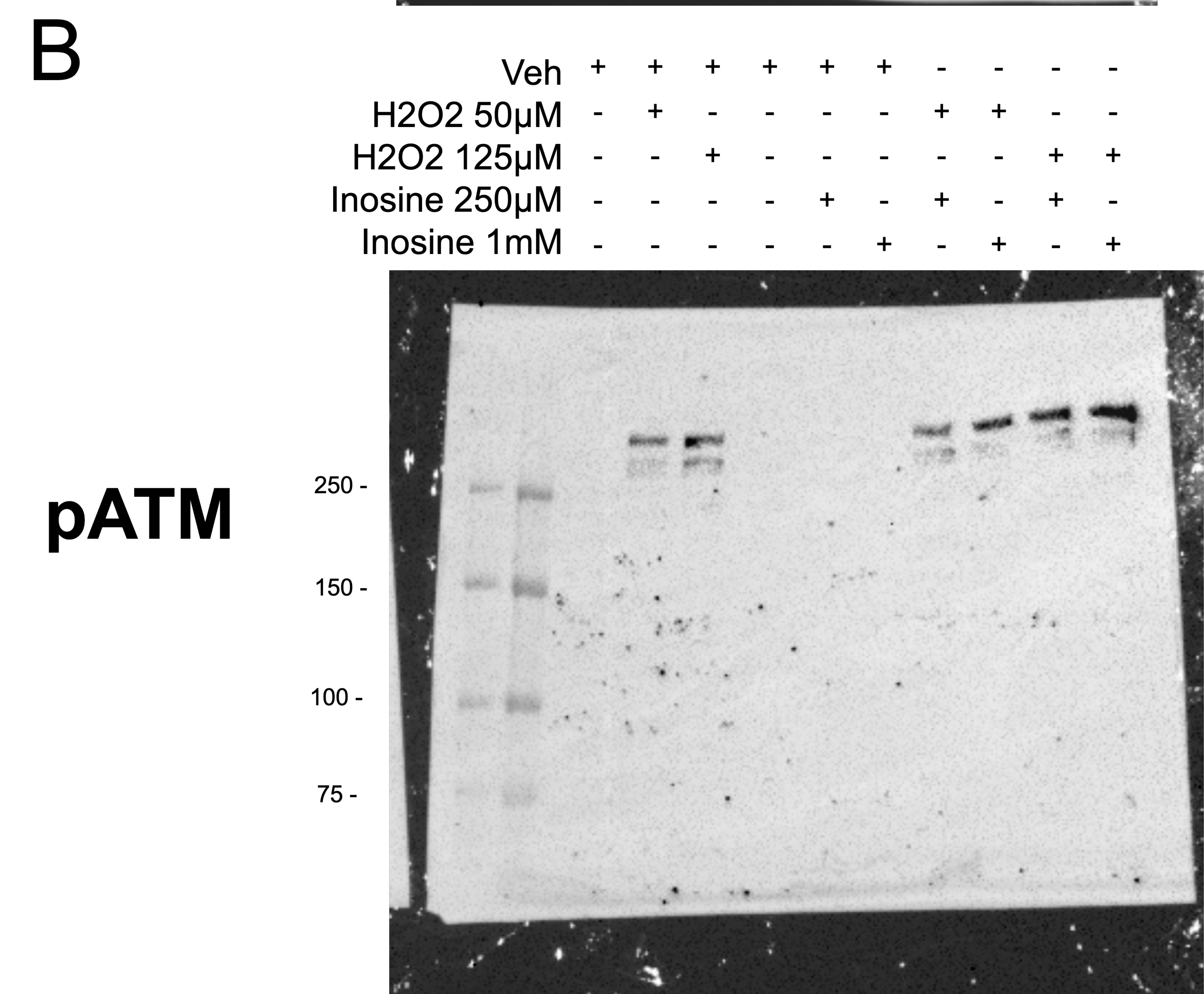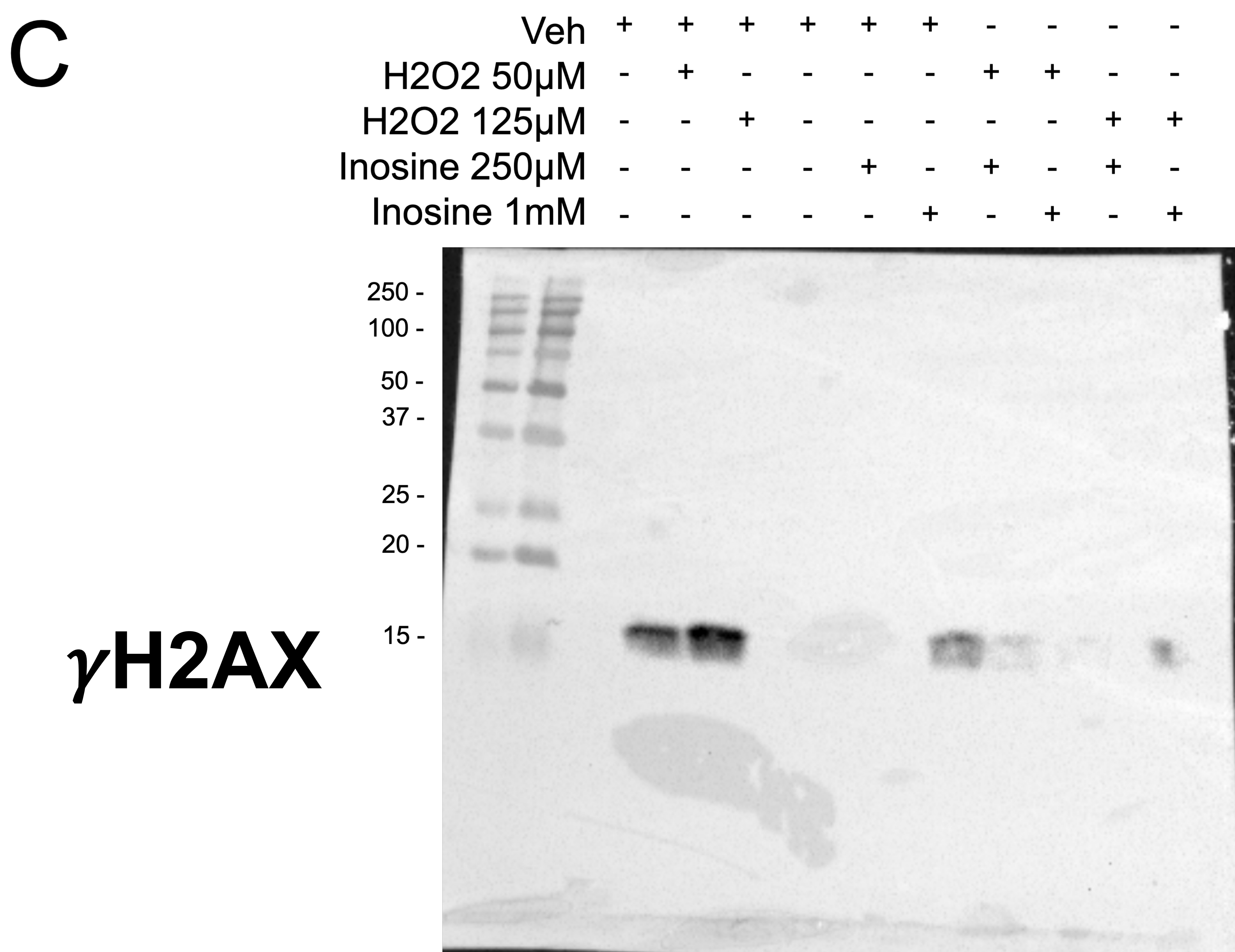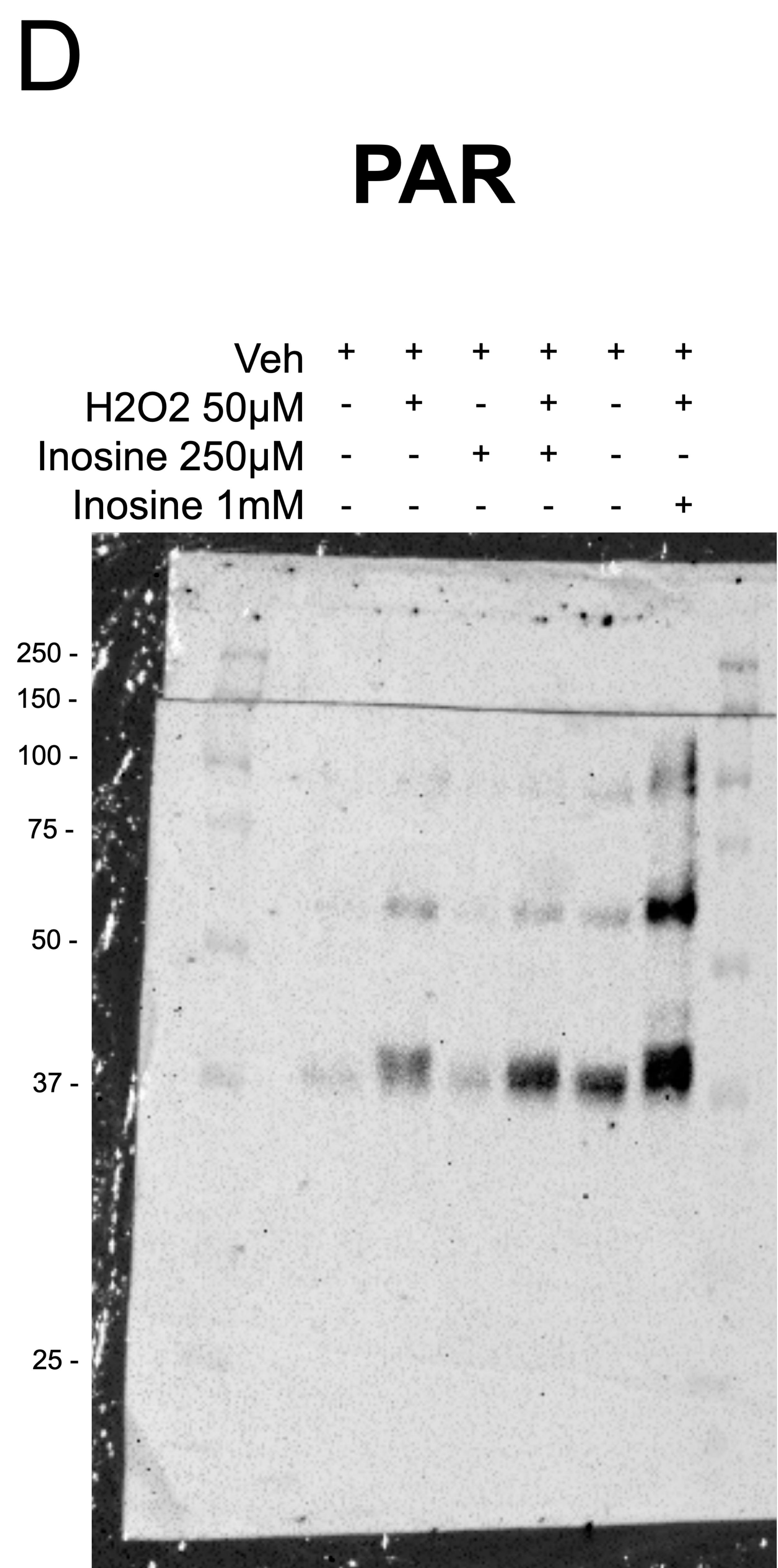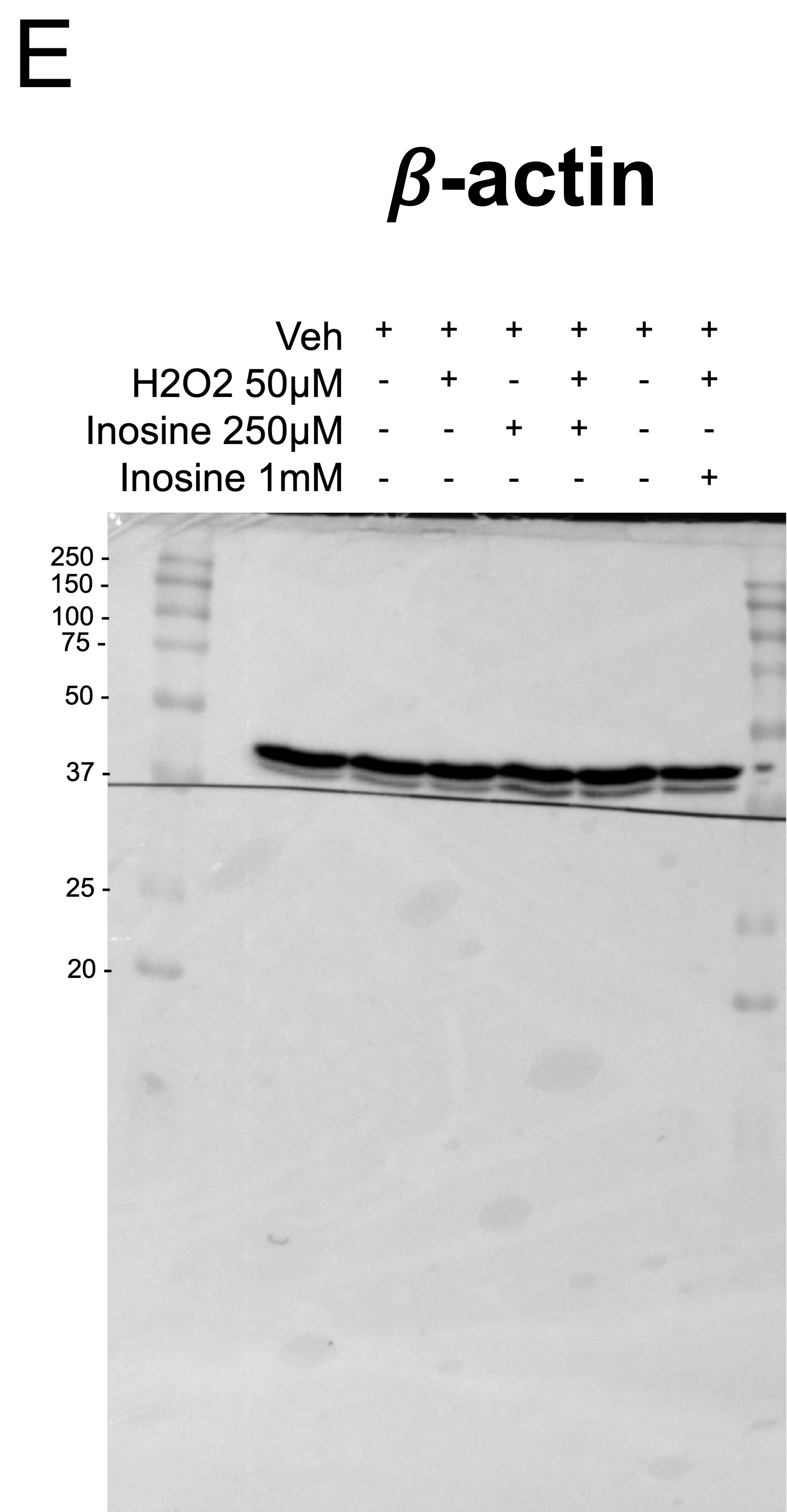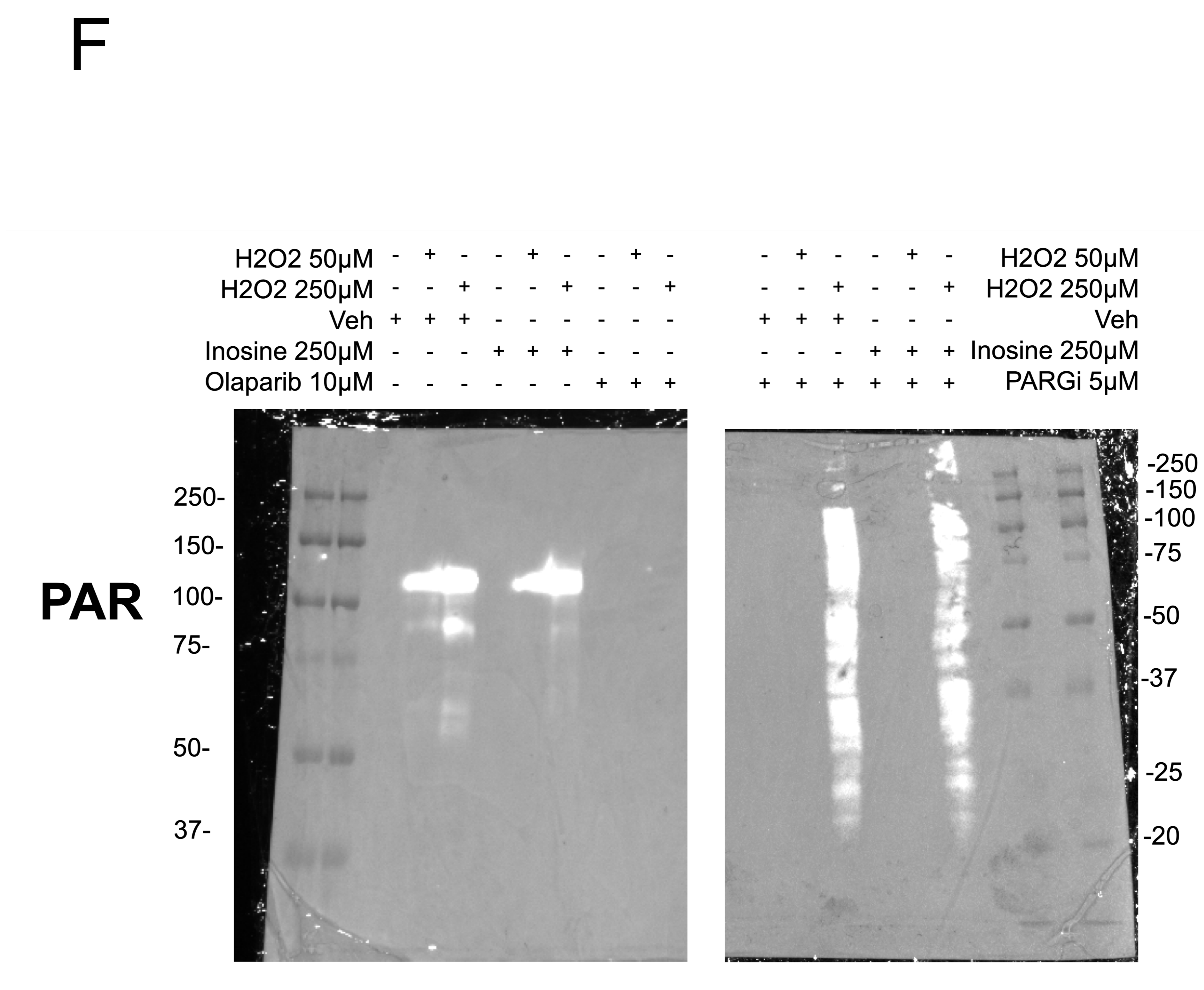

**Supplementary Figure 17 Uncropped blots for Figure 8 panel D**

Supplement: Unedited blot and gel images [file jciinsight-10-180275-s054.pdf]
