# Supplementary material for: Multiomics analysis unveils an inosine-sensitive DNA damage response in neurogenic bladder after spinal cord injury
Source: JCI Insight. 2025 May 8;10(12):e180275. doi: 10.1172/jci.insight.180275 (PMC12220959; doi:10.1172/jci.insight.180275)
Supplement: Supplemental materials [file jciinsight-10-180275-s055.pdf]

Supplementary Information for

Multiomics analysis unveils an inosine-sensitive DNA damage response in neurogenic bladder after spinal cord injury

Ali Hashemi Gheinani[1,2,3,4,5,*,#], Bryan S. Sack[1*,^], Alex Bigger-Allen[1,4,5,*], Hatim Thaker[1,4], Hussein Atta[1,4], George Lambrinos[1,4], Kyle Costa[1], Claire Doyle[1,4], Mehrnaz Gharaee-Kermani[6], Susan Patalano[6], Mary Piper[7], Justin F. Cotellessa[6], Dijana Vitko[1,4], Haiying Li[1,4], Manubhai Kadayil Prabhakaran[1,4], Vivian Cristofaro[4,8], John Froehlich[1,4], Richard S. Lee[1,4], Wei Yang[9], Maryrose P. Sullivan[4,8], Jill A. Macoska[6], Rosalyn M. Adam[1,4,5,#]

[#]Correspondence: ali.hashemi@unibe.ch; rosalyn.adam@childrens.harvard.edu

This document file includes:

SI Materials and Methods

SI References

Supplementary Figures 1-17 and Legends

Supplementary Table 1

Supplementary Methods

NanoString Panel Selection and Hybridization

Bladder tissues were harvested from rats subjected to spinal cord injury (SCI) and age-matched uninjured controls at designated timepoints (2, 8, and 16 weeks after injury). Tissues were snap-frozen in liquid nitrogen and stored at -80°C until processing. Total RNA was extracted using the RNeasy Mini Kit (QIAGEN, Hilden, Germany) following the manufacturer's protocol, including on-column DNase treatment to remove genomic DNA contamination. RNA quality and quantity were assessed using the Agilent Bioanalyzer 2100 (Agilent Technologies, Santa Clara, CA, USA) to ensure RNA integrity number (RIN) ≥7 for inclusion in downstream analysis. A custom NanoString nCounter mRNA panel was designed to target genes involved in key pathways implicated in SCI-induced bladder remodeling, including oxidative stress, inflammation, extracellular matrix (ECM) remodeling, and DNA damage response. This panel included 55 genes, selected based on prior RNA-seq data, literature review, and relevance to the study hypotheses as well as housekeeping genes. Each sample (100 ng of RNA) was hybridized with the NanoString probes according to the manufacturer's instructions using the nCounter XT CodeSet. Hybridized samples were processed on the NanoString nCounter Analysis System to quantify gene expression. Raw counts were exported, and quality control metrics were assessed to ensure sufficient probe binding and data integrity. Raw data were normalized using NanoString nSolver software (version 4.0) with background subtraction and normalization to housekeeping genes included in the panel. Differential expression analysis was performed to compare SCI and control samples at each timepoint.

Scatterplots of logFC vs logCPM

The ggplot R package was used to create the scatterplots of log fold change (logFC) vs versus log counts per million (logCPM) for the top 500 differentially expressed genes between two groups. The X-axis represents the logarithmic fold change and the Y-axis shows the logarithmic count per million (CPM), which is a measure of the expression level of the genes in each sample. Additionally, red vertical lines are drawn at logFC = -2 and logFC = 2, and a red horizontal line is drawn at logCPM = 5. In this plot, each point represents a gene, and its position is determined by its fold change and CPM values. The genes are colored based on their p-value.

Heatmaps and clustering

Hierarchical clustering and heatmaps were created using the "heatmap2" function available in the R package "Gene-E". To construct the hierarchical clustering, we first computed a pairwise correlation matrix between the items, employing the Pearson correlation method. The correlation matrix was then transformed into a

distance matrix, with subsequent clustering performed using the average linkage method, which utilizes the average distance for distance matrix calculation. Heatmaps were generated to visualize the hierarchical clustering results, specifically focusing on the differentially expressed genes (DEGs) within various comparison groups. DEGs were defined as those with a log2 fold change greater than or equal to ±0.5, a p-value less than 0.05, and read counts exceeding 1 count per million (cpm).

Circos plots

We utilized Circos plots for the visualization and analysis of top canonical pathways and their corresponding enriched genes or proteins, with the analysis conducted using the "circlize" package in R. The selection of top canonical pathways was based on the negative logarithm of p-values (Neg.log.p_value>1.3). To prepare the data for visualization, we transformed it by splitting the molecules within selected pathways and creating new rows for individual molecules. To establish the connections between pathways and molecules, we constructed a links data frame, serving as a representation of these relationships. Setting up the chord diagram visualization involved configuring parameters such as gap degree and sector labels. For improved clarity, we implemented filters to include only the most significant molecules, considering criteria such as appearance in multiple pathways or highest significance values. The final output was a chord diagram that portrayed the associations between the top canonical pathways and enriched genes.

Z score calculation for activity of canonical pathways.

Z score (IPA (application build 127006219, dated January 26, 2025, content version 127006219)) was used to mathematically compare the uploaded data set (differentially expressed mRNAs) with the canonical pathway patterns. The significance values for the canonical pathways were calculated by right-tailed Fisher's exact test.

Upstream regulator analysis.

The upstream pathway analysis module of IPA (application build 127006219, dated January 26, 2025, content version 127006219) was used. Overlapping P values were calculated by IPA using Fisher's exact test based on the significance of the overlap between the known targets and an experimentally identified set of regulated genes.

Quantitative Image Analysis

For quantitation of signal, a minimum of 5-15 images were taken at random from both detrusor and mucosa such that a minimum of 50% of the field of view was occupied by that compartment. The images varied in

number based on the size of the compartment represented in the tissue section. Images for each of the three conditions (control, SCI-Vehicle- and SCI-Inosine) were analyzed in each of 3 biological replicates with an in-house developed macro leveraging ImageJ macro language. Briefly, the macro creates a duplicate of the original .czi image file, and converts it to an 8-bit tiff image that is separated into 3 constituent channels representing DAPI, signal from one of three antibodies that detect gamma-H2AX, pATM-substrates, or PAR, and a secondary reference marker, namely alpha-smooth muscle actin, SM22 or pan-cytokeratin. A binary version of the DAPI signal was created and used to make regions of interest (ROI) that represent the nuclei. A binary image was also created from the channel containing the reference marker. The nuclear ROIs were then superimposed onto the binary reference-signal image to determine if each nuclear ROI was contained within a region that was positive or negative for the secondary reference marker. Finally, the nuclear ROIs were superimposed onto the unmodified channel representing the primary marker of interest and intensity measurements were made. For each nuclear ROI, a variety of measurements were saved including the integrated density, median intensity of both the primary and reference markers, roundness, and area. All images were processed through this macro to ensure that all images were quantified in the same manner. No thresholding parameters were utilized to remove nuclear ROIs from the dataset. All data filtering, quality control assessments, and final analyses were performed in R. The process and output of the macro is summarized in Supplementary Figure 13.

Quantitative Proteomics Analysis

Tryptic peptides were labeled with 11-plex TMT reagents in parallel, merged into one sample, desalted using C18 spin columns (Thermo Scientific), and dried down in a SpeedVac (Thermo Scientific). Each set of TMT11plex-labeled peptide mixture was fractionated into 48 fractions by high-pH liquid chromatography and concatenated into 16 fractions by combining fractions 1, 17, 33; 2, 18, 34; and so on. The concatenated fractions were concentrated in a SpeedVac and stored at -80°C. LC-SPS-MS3 analysis was performed on an EASY-nLC 1200 connected to an Orbitrap Fusion Lumos mass spectrometer (Thermo Scientific). Each fraction of TMT11plex-labeled peptides was resuspended in 0.2% formic acid, and about 1 μg peptide was loaded onto a 2-cm trap column and separated by a 50-cm EASY-Spray column (Thermo Scientific) heated to 55°C, using a 3-h gradient at a flow rate of 250 nL/min. The parameter settings for FTMS1 include orbitrap resolution of 120K, scan range of m/z 350-1400, maximum injection time of 100 ms, AGC target of 5E5, RF lens of 30%, data type of centroid, charge state of 2-5, dynamic exclusion for 60 s using a mass tolerance of 7 ppm, and internal calibration using 371.10123. The parameters for ITMS2 include mass range of 400-1400 m/z, 10 dependent scans, isolation window of 0.4 m/z, CID collision energy of 35%, maximum injection time of 120 ms, AGC target of 2E4, and data type of centroid. The

parameters for MS3 include scan range of 100-1000 m/z, maximum injection time of 150 ms, AGC target of 2.5E5, HCD collision energy of 55%, and data type of centroid.

Because the total number of samples (20) in our proteomics experiment exceeded the maximum number for one set of isobaric tag reagents (11), we ran two TMT 11-plex experiments. In this case, each TMT experiment contained a pool of all 20 samples. The channels pooled reference mixture were used to match the protein reporter ion intensities between TMT experiments. This was done by dividing impurity corrected TMT intensity of each ID by the intensity value of its TMT pool, resulting in a pool normalization factor and then this factor was multiplied to the impurity corrected TMT intensities for that ID (for all samples). Further, we reasoned that there might be many sources of variation in addition to the biological differences between groups due to capacity of TMT experiment for running all samples; therefore, we designed a normalization pipeline to minimize the errors imposed by running samples in two batches. For TMT labeling, since we used the same amount of digested protein labeled for each channel, the liberated reporter ion signals were the proxies for protein abundance, so the sum of the reporter ions in each channel was a proxy for the total amount of protein and the total signal per channel was checked for consistency. We have compared two normalization methods. First, we performed normalization to abundant proteins and compared it with the second method where we used internal reference scaling (IRS) methodology (1) since it has been reported to be capable of correcting the random MS2 sampling that occurs between TMT experiments.

References

1. Plubell DL, et al. Extended Multiplexing of Tandem Mass Tags (TMT) Labeling Reveals Age and High Fat Diet Specific Proteome Changes in Mouse Epididymal Adipose Tissue. *Mol Cell Proteomics* 2017 16: 873-90.

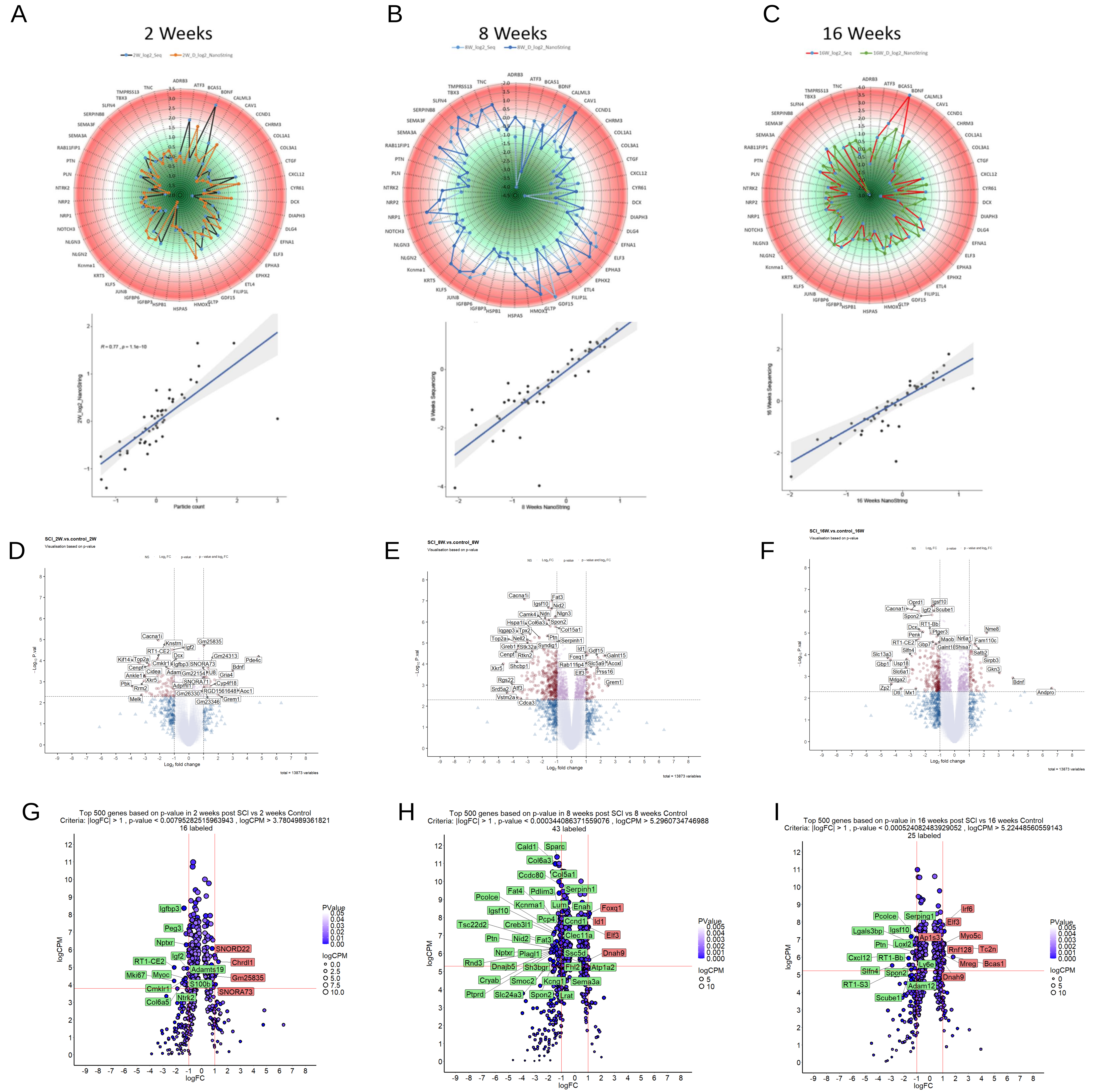

Supplementary Figure 1

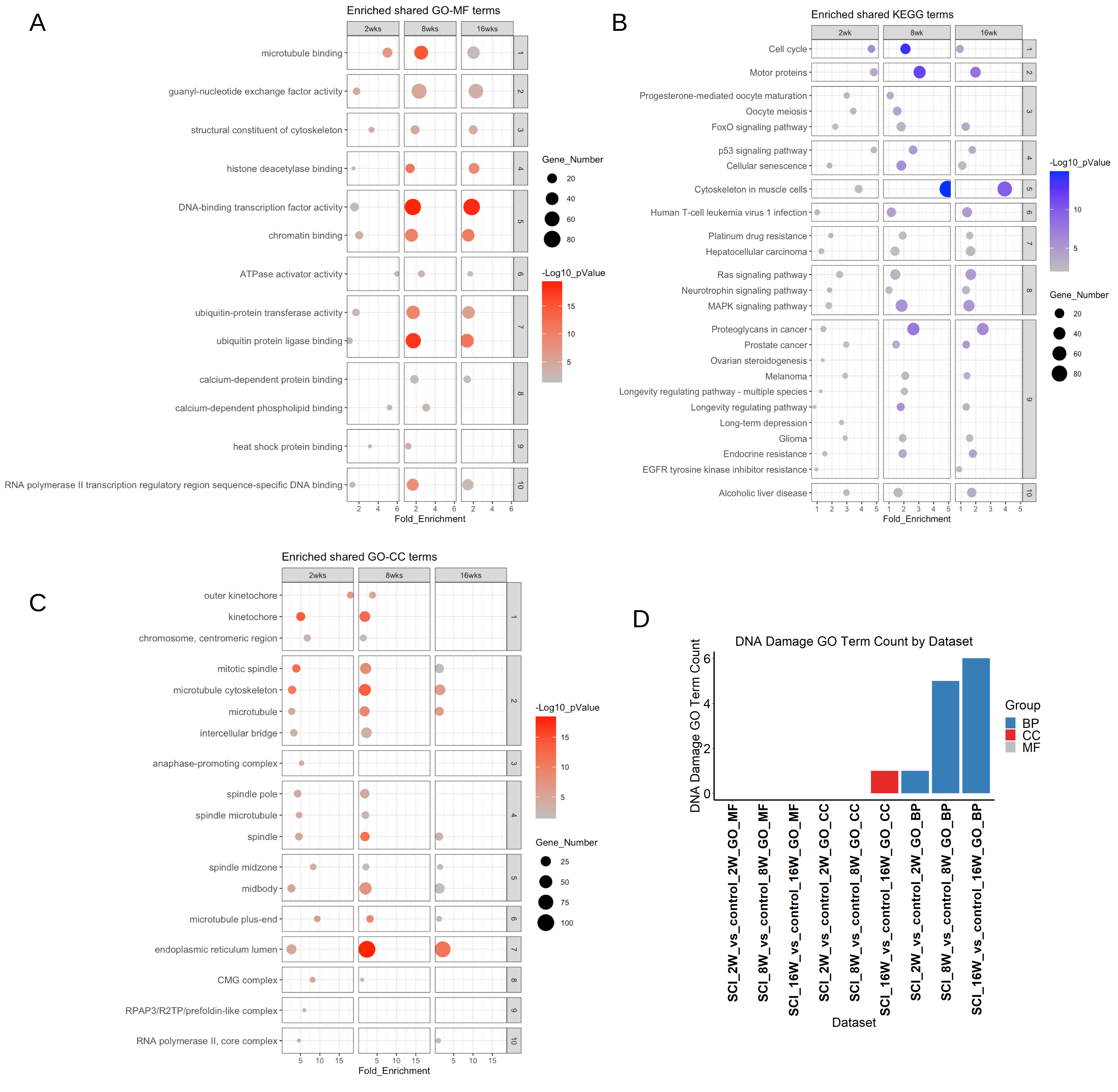

Supplementary Figure 2

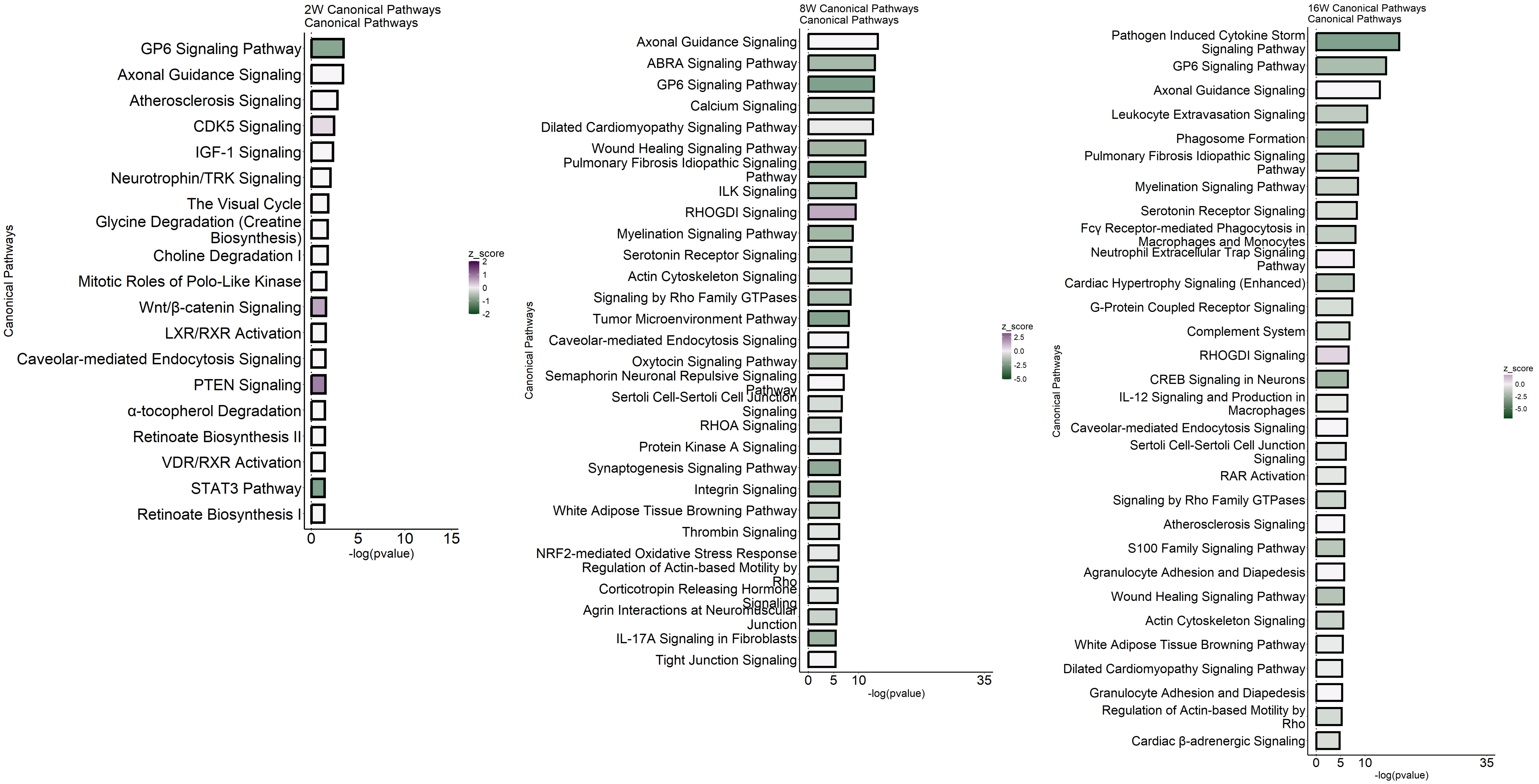

Supplementary Figure 3

Analysis Overview

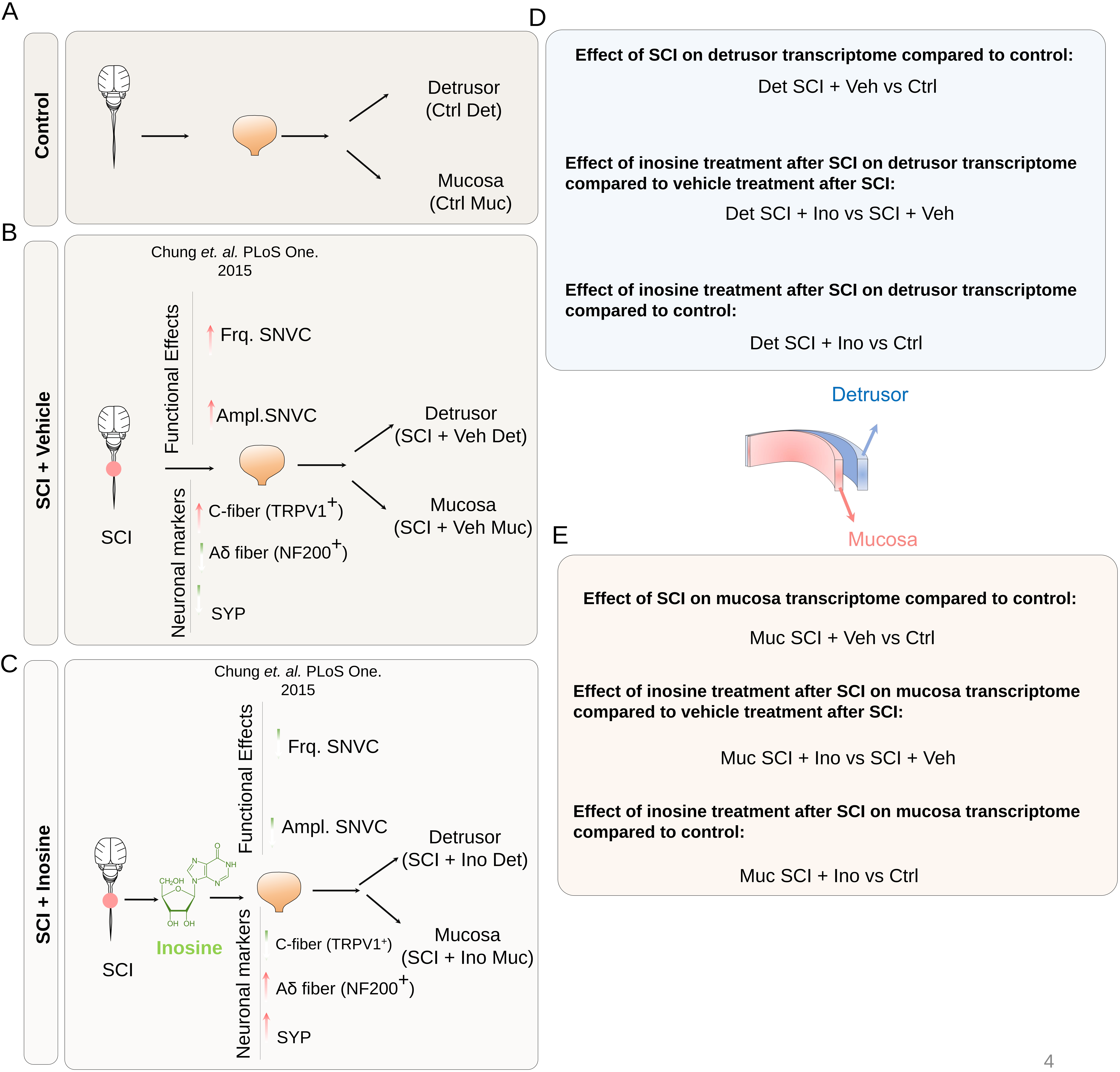

Supplementary Figure 4

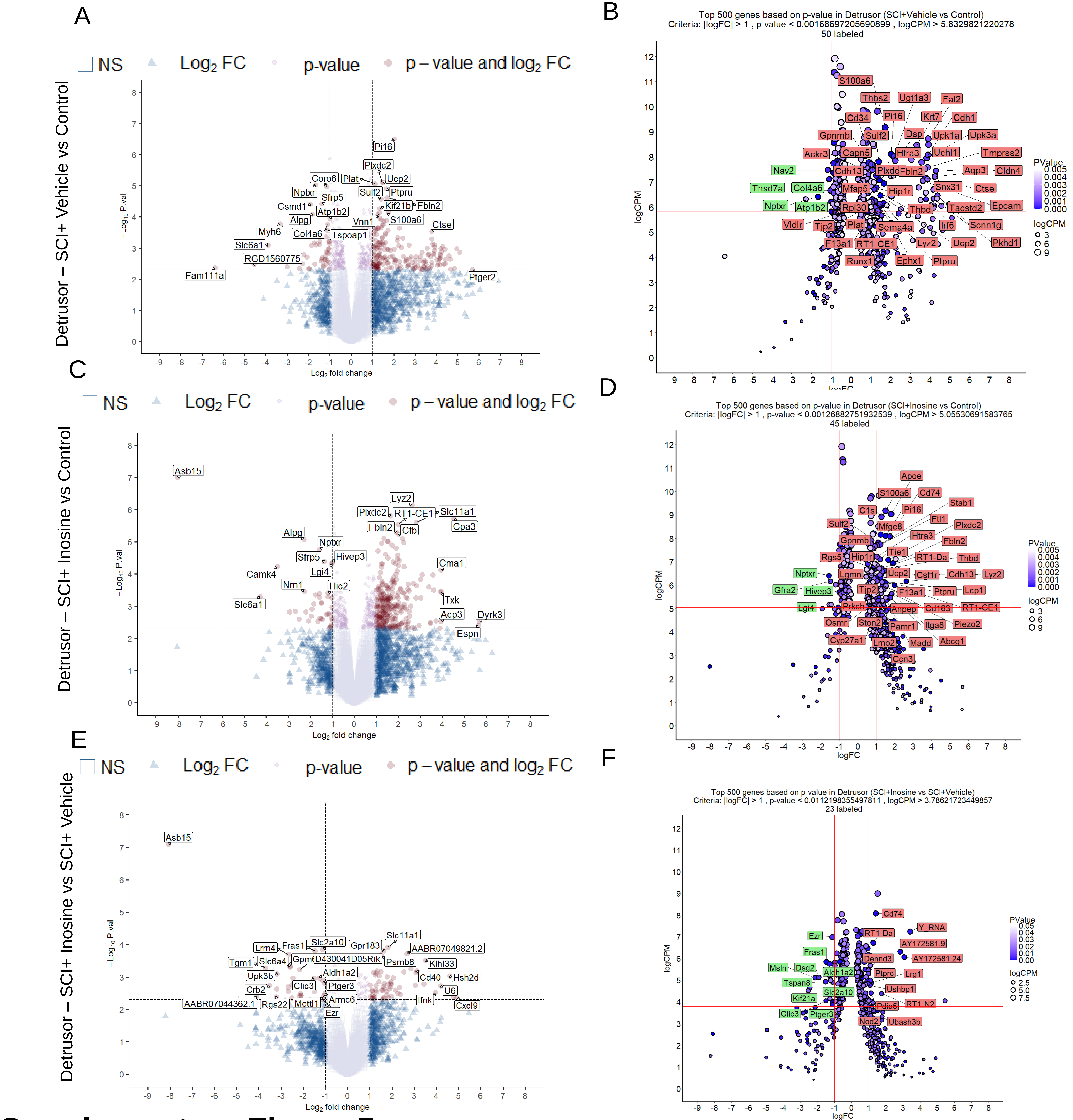

Supplementary Figure 5

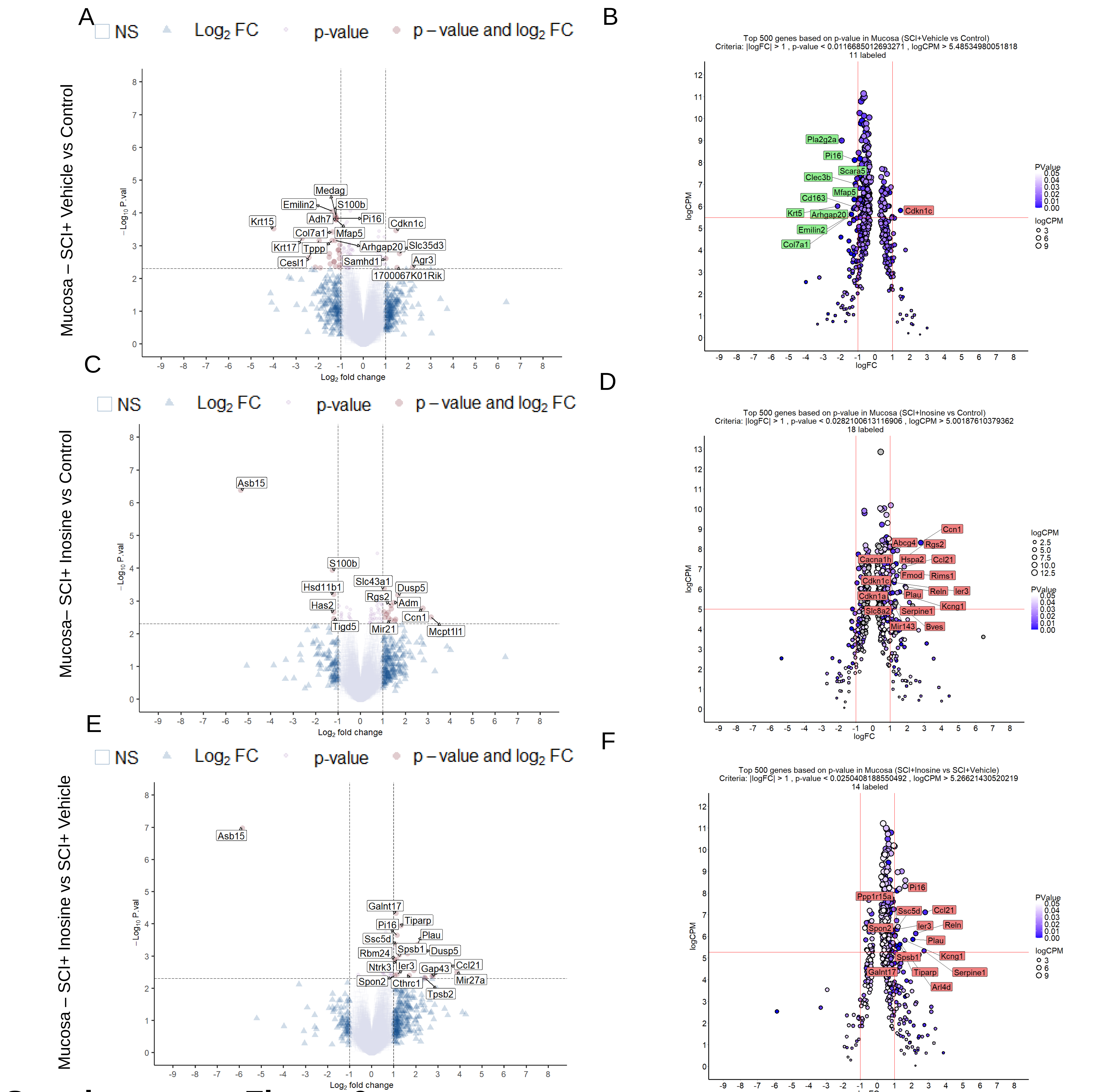

Supplementary Figure 6

A Inosine responsive genes in detrusor

B Inosine responsive genes in mucosa

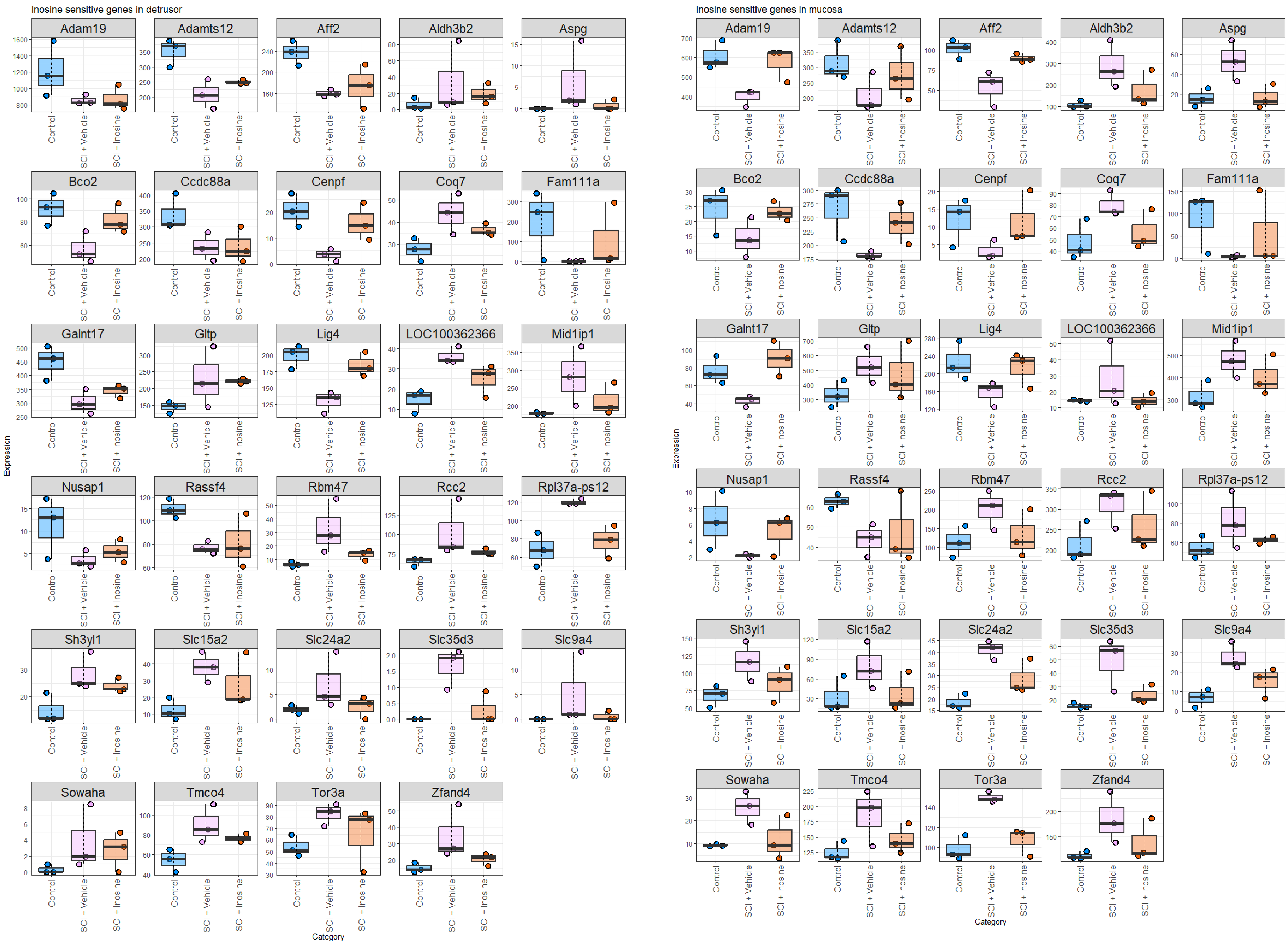

Figure Supplementary 7

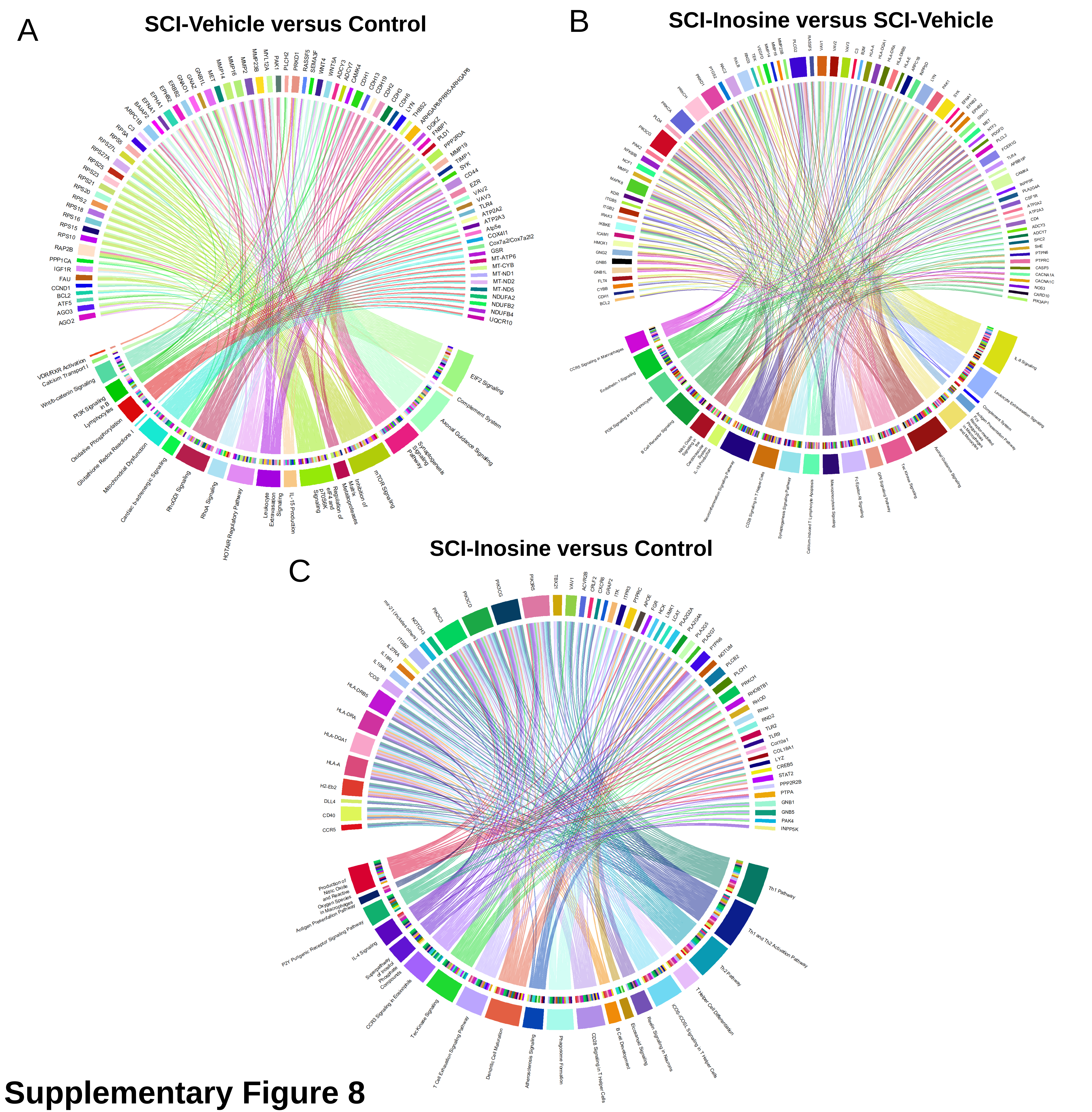

Supplementary Figure 8

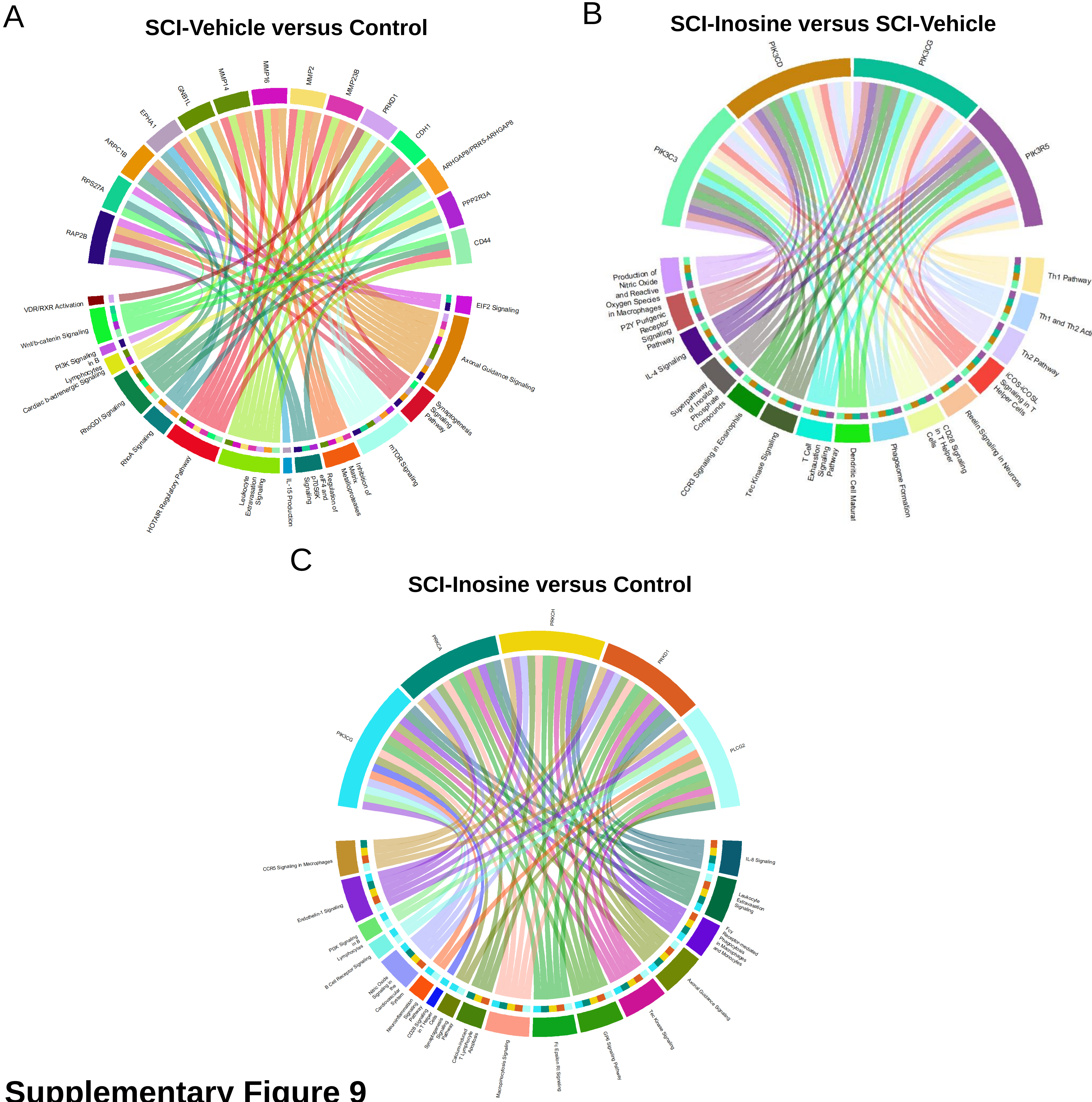

Supplementary Figure 9

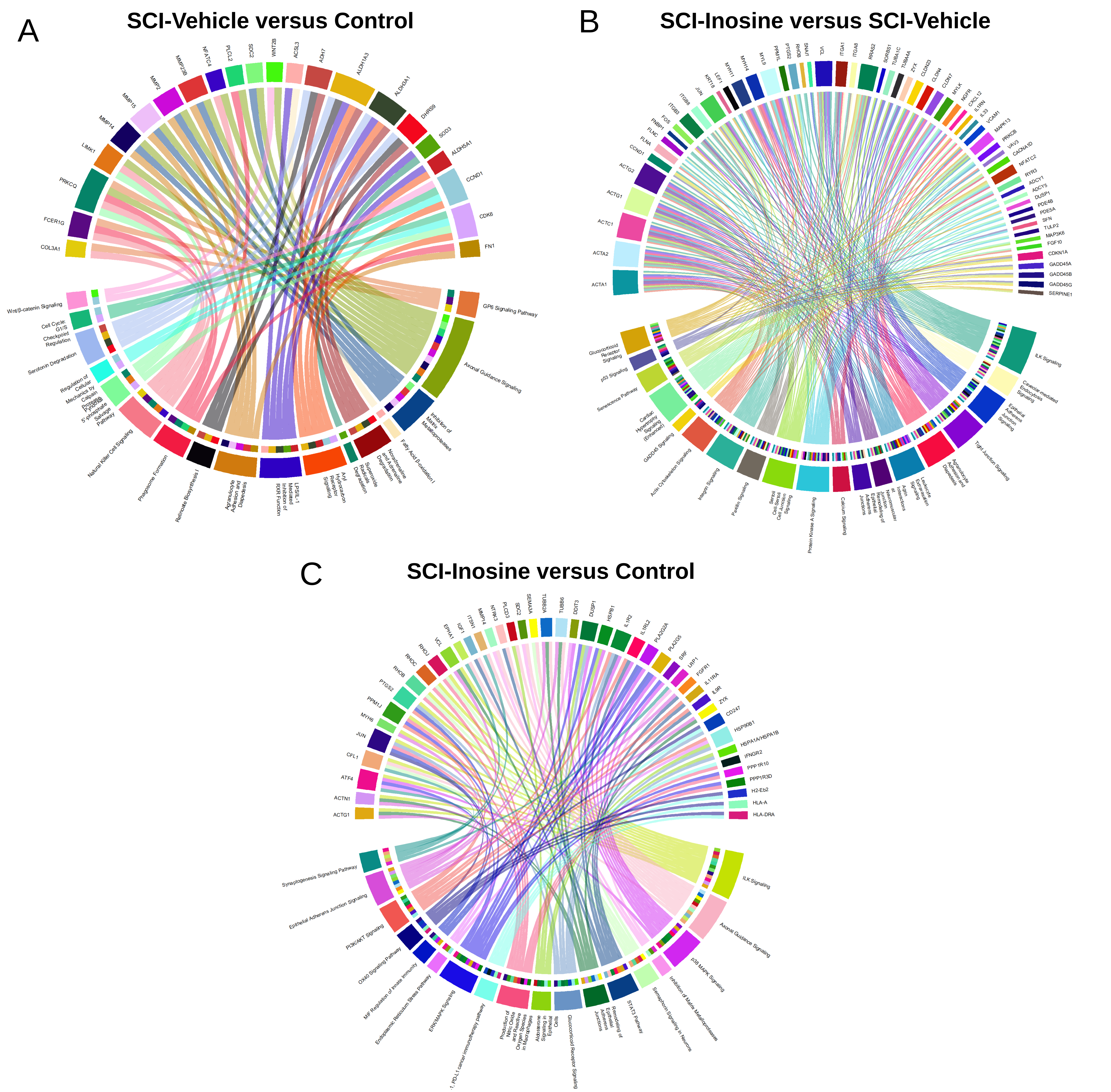

Supplementary Figure 10

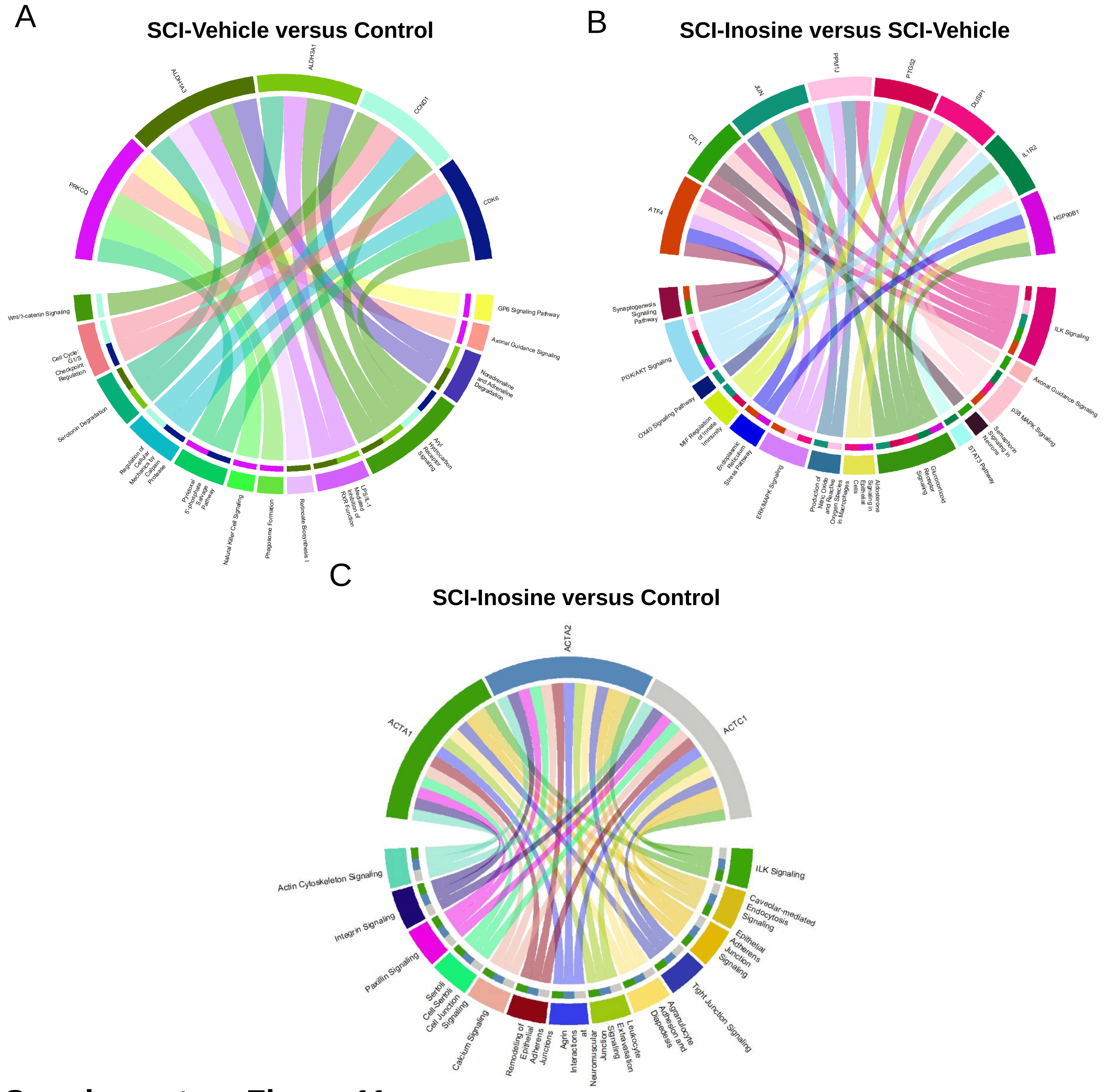

Supplementary Figure 11

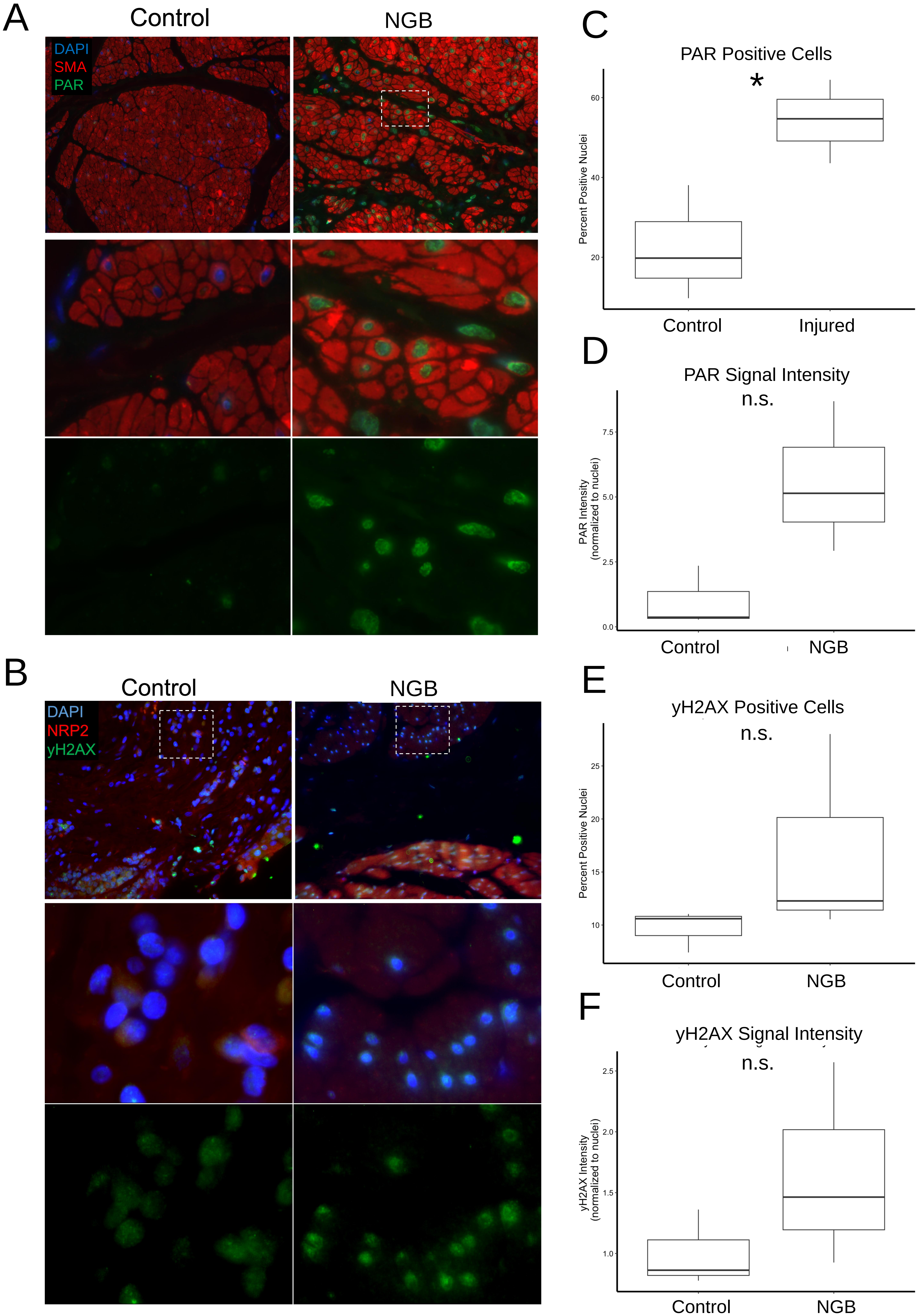

Supplementary Figure 12

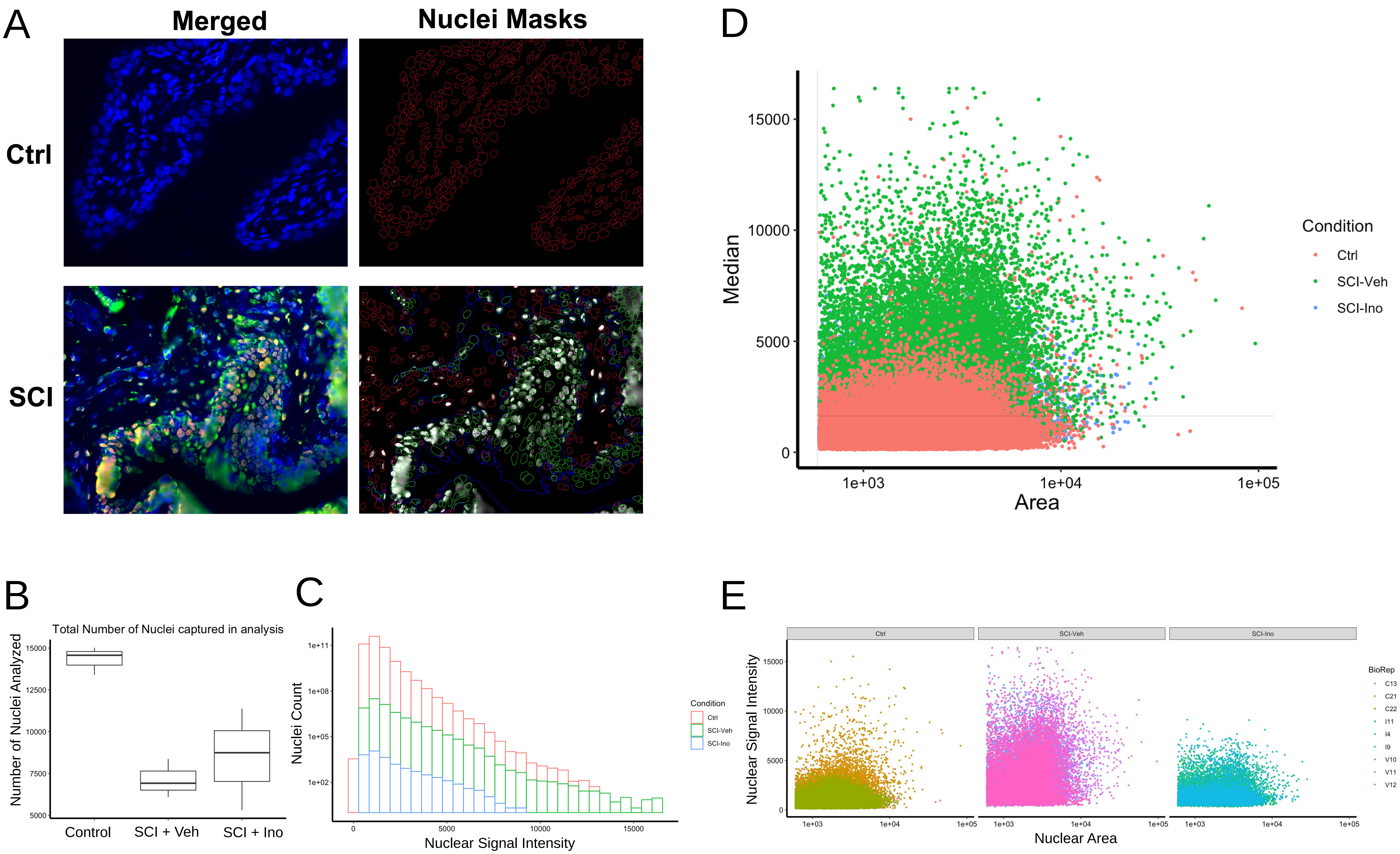

Supplementary Figure 13

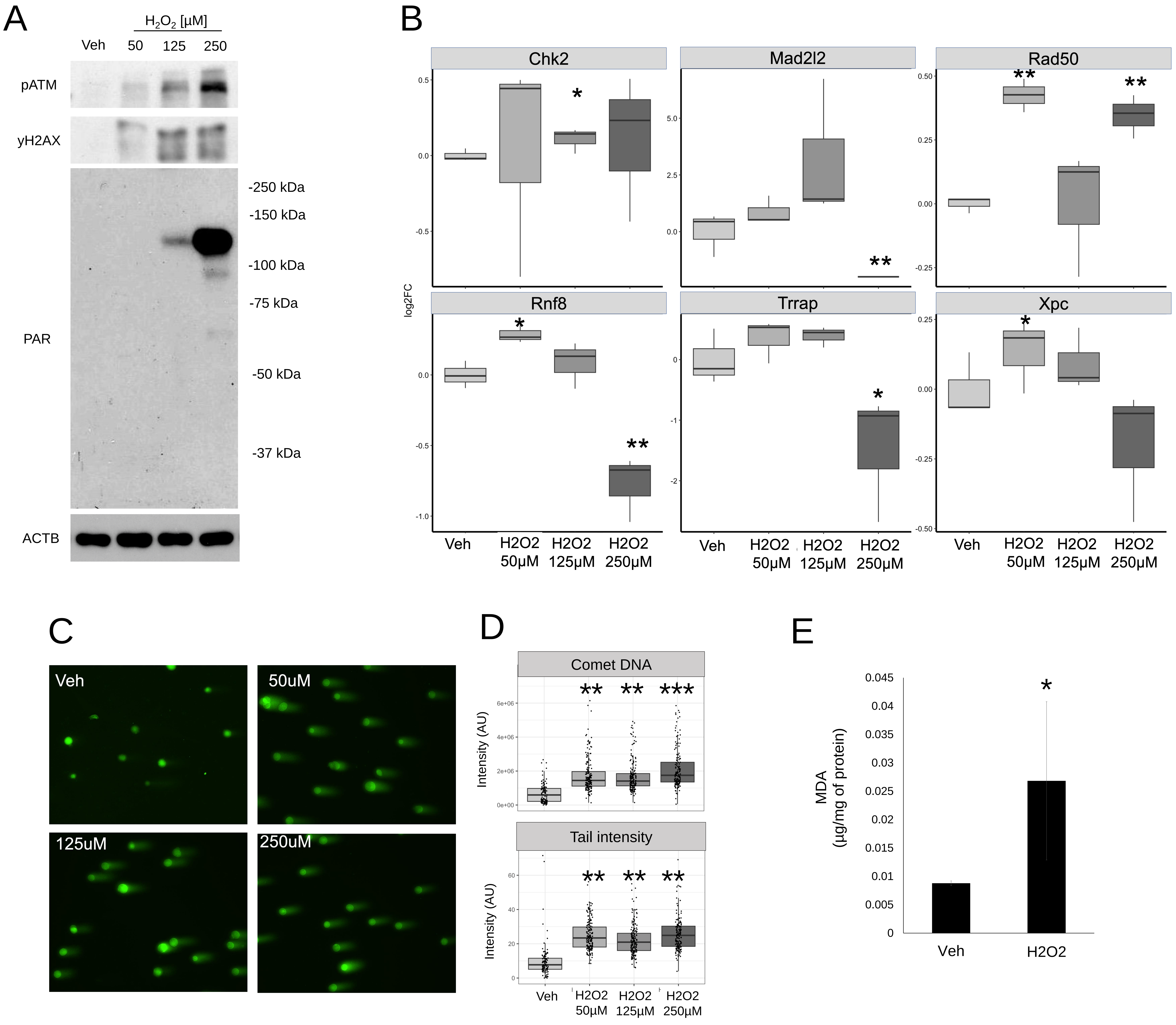

Supplementary Figure 14

1. Regulation of eIF4 and p70S6K Signaling
2. Mitochondrial Dysfunction
3. EIF2 Signaling
4. NRF2-mediated Oxidative Stress Response
5. Sirtuin Signaling Pathway
6. Signaling by Rho Family GTPases
7. Oxidative Phosphorylation
8. Acute Phase Response Signaling
9. Integrin Signaling
10. Thrombin Signaling
11. CDK5 Signaling
12. mTOR Signaling
13. Actin Cytoskeleton Signaling
14. RhoGDI Signaling
15. Clathrin-mediated Endocytosis Signaling
16. Epithelial Adherens Junction Signaling
17. Synaptogenesis Signaling Pathway
18. Caveolar-mediated Endocytosis Signaling
19. α-Adrenergic Signaling
20. Gα12/13 Signaling
21. RhoA Signaling
22. Leukocyte Extravasation Signaling
23. Hepatic Fibrosis Signaling Pathway
24. Unfolded protein response
25. Cardiac β-adrenergic Signaling
26. Xenobiotic Metabolism General Signaling Pathway
27. Dopamine-DARPP32 Feedback in cAMP Signaling
28. Macropinocytosis Signaling
29. Nitric Oxide Signaling in the Cardiovascular System
30. Cellular Effects of Sildenafil (Viagra)
31. BMP signaling pathway
32. Apelin Adipocyte Signaling Pathway
33. Phagosome Maturation
34. Fcγ Receptor-mediated Phagocytosis in Macrophages and Monocytes
35. Endothelin-1 Signaling
36. FXR/RXR Activation
37. Glycogen Degradation II
38. Glycogen Degradation III
39. Autophagy
40. Glutathione Redox Reactions II
41. Sperm Motility
42. Complement System
43. HOTAIR Regulatory Pathway
44. Arsenate Detoxification I (Glutaredoxin)
45. Acetate Conversion to Acetyl-CoA
46. Amyotrophic Lateral Sclerosis Signaling
47. Vitamin-C Transport
48. UDP-N-acetyl-D-glucosamine Biosynthesis II
49. White Adipose Tissue Browning Pathway

A

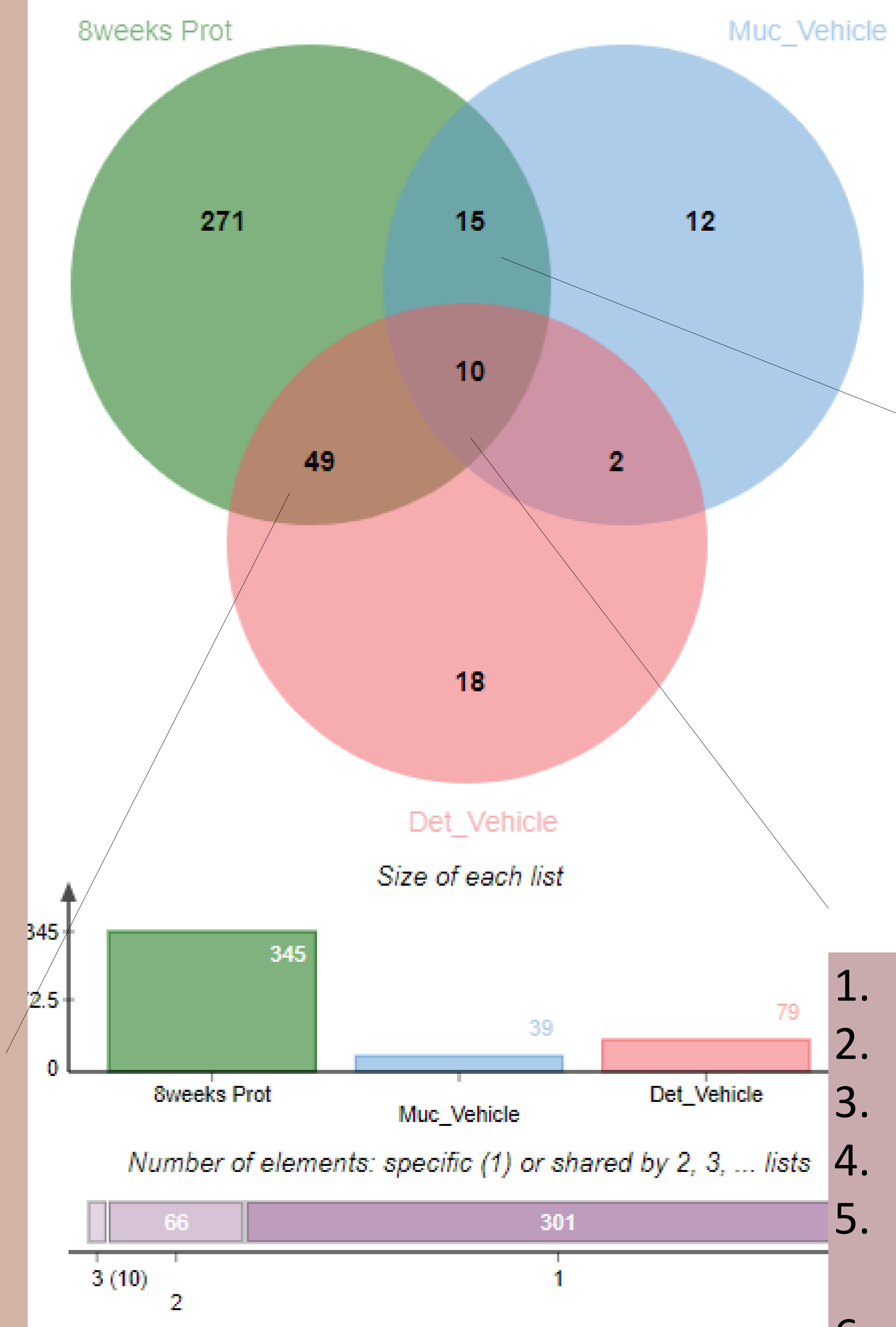

1. Xenobiotic Metabolism CAR Signaling Pathway
2. HIF1α Signaling
3. Intrinsic Prothrombin Activation Pathway
4. Pyridoxal 5'-phosphate Salvage Pathway
5. Aryl Hydrocarbon Receptor Signaling
6. HER-2 Signaling in Breast Cancer
7. Fatty Acid β-oxidation I
8. Circadian Rhythm Signaling
9. Superoxide Radicals Degradation
10. Regulation of Cellular Mechanics by Calpain Protease
11. Natural Killer Cell Signaling
12. Role of Macrophages, Fibroblasts and Endothelial Cells in Rheumatoid Arthritis
13. Extrinsic Prothrombin Activation Pathway
14. Ethanol Degradation II
15. LPS/IL-1 Mediated Inhibition of RXR Function

1. Estrogen Receptor Signaling
2. Protein Kinase A Signaling
3. Axonal Guidance Signaling
4. GP6 Signaling Pathway
5. Hepatic Fibrosis / Hepatic Stellate Cell Activation
6. Ethanol Degradation IV
7. Inhibition of Matrix Metalloproteases
8. Glutathione Redox Reactions I
9. Wnt/β-catenin Signaling
10. Osteoarthritis Pathway

B

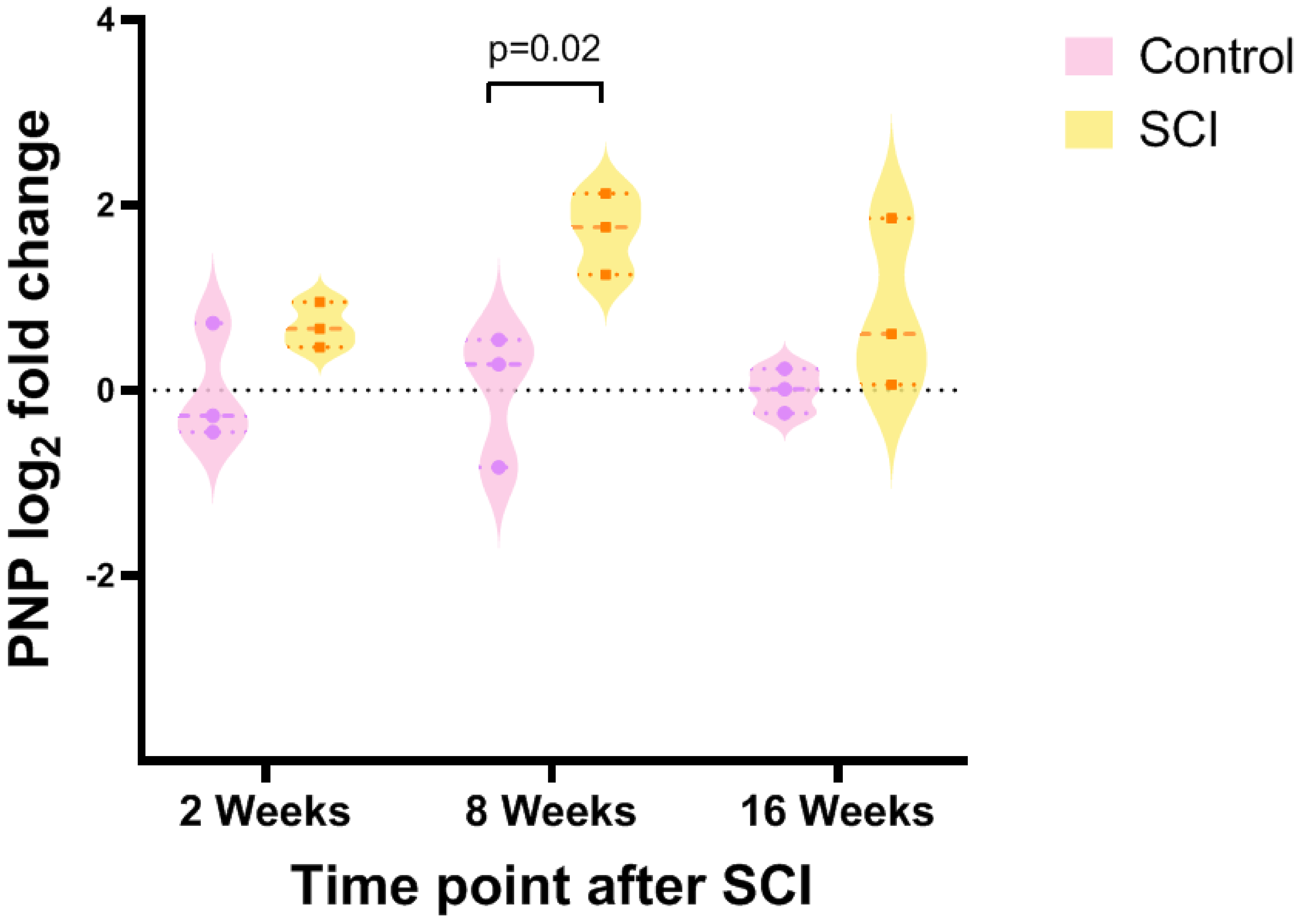

Supplementary Figure 15

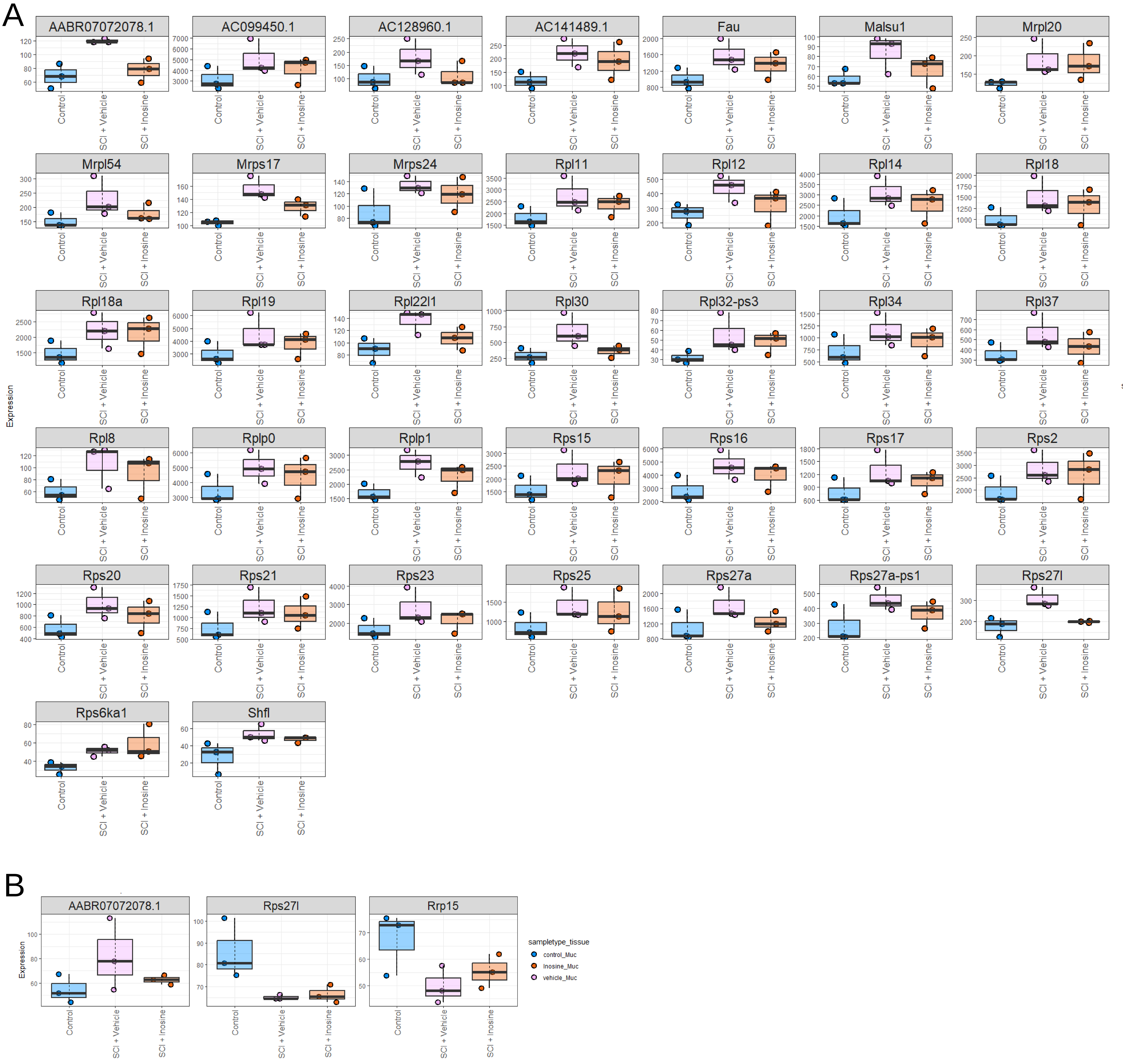

Supplementary Figure 16

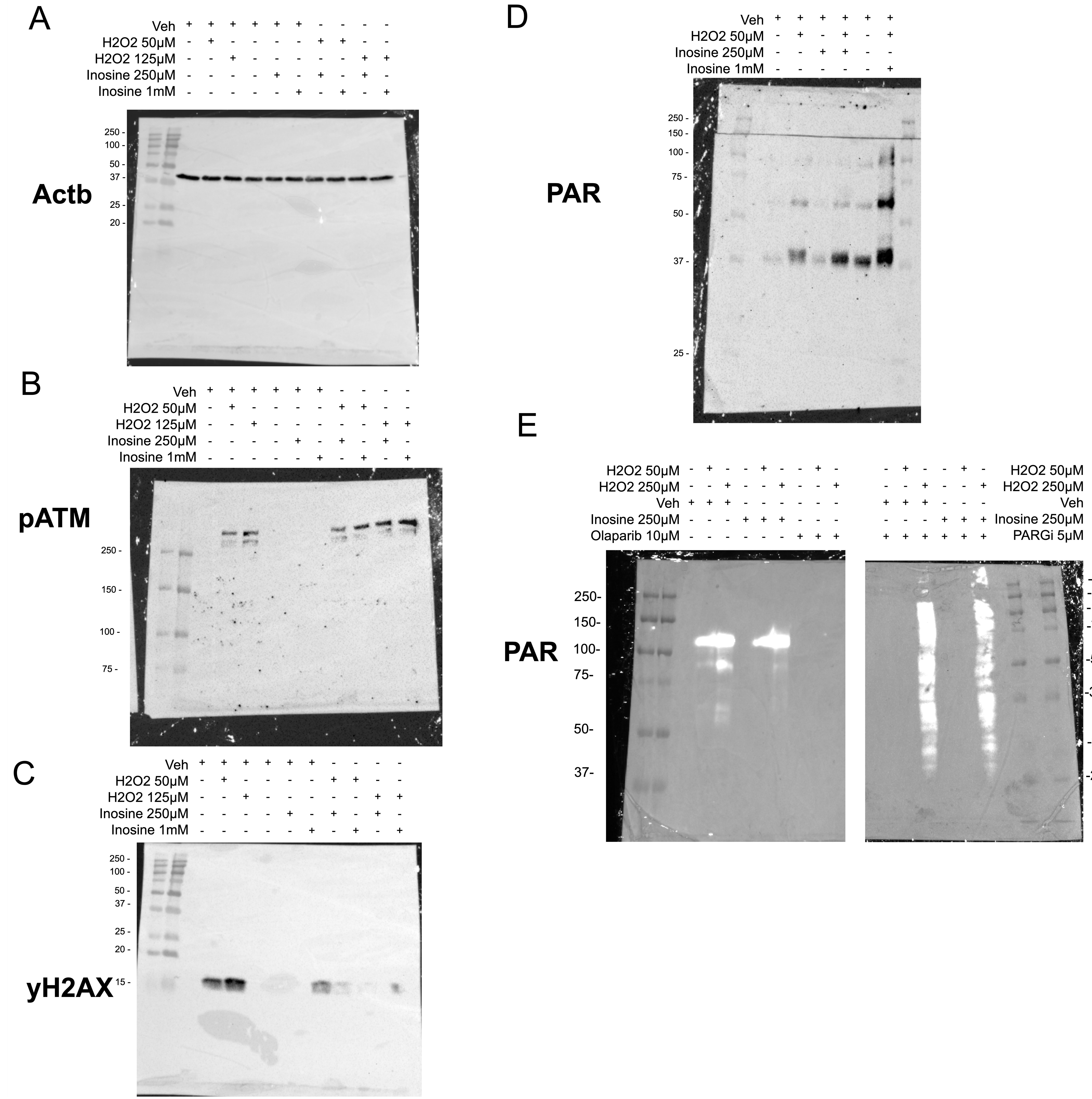

Supplementary Figure 17

Supplementary Figure Legends

Supplementary Figure 1. Validation of differentially expressed genes (DEGs) using NanoString analysis in control and SCI rat bladders across three time points.

Radial plots show log2 fold changes of selected DEGs comparing RNA sequencing and NanoString data at **(A)** 2 weeks (RNA-seq (blue line);NanoString (orange line), **(B)** 8 weeks (RNA-seq (light blue line); NanoString (dark blue line) and **(C)** 16 weeks (RNA-seq (red line); NanoString (green line) after SCI (n=6-7 biological replicates per time point). DEGs are arranged around the circle, with red shading indicating up- and green shading indicating down-regulated genes. For each time point, scatter plots below the radial plots display the correlation between RNA-seq (y-axis) and NanoString (x-axis) log2 fold changes. Linear regression analysis shows strong correlation at each time point, as indicated by Pearson correlation coefficients and p-values: 2 weeks, $R = 0.77$, $p = 1.1e-10$; 8 weeks, $R = 0.87$, $p < 0.001$; 16 weeks, $R = 0.89$, $p < 0.001$. Volcano plots of DEGs in bladder samples at 2 wks **(D)**, 8 wks **(E)** and 16 wks **(F)** after SCI versus age-matched controls. The horizontal dotted line represents the cutoff for statistical significance (p-value < 0.05); vertical dotted lines indicate a log fold change (FC) of -2 and 2, representing thresholds for down- and up-regulation, respectively. Scatterplots of DEGs in bladder samples at 2 wks **(G)**, 8 wks **(H)** and 16 wks **(I)** after SCI versus age-matched controls, showing the top 500 DEGs ranked by p-value, with log (FC) on the x-axis and log counts per million (CPM) on the y-axis. Genes meeting the significance criteria (|logFC|, p-value, logCPM) are labeled green if downregulated (logFC < -1), and red if upregulated (logFC > 1). The size of each point corresponds to logCPM values, and the color gradient represents the p-value (ranging from blue to white). Red vertical and horizontal lines indicate the logFC and logCPM thresholds used for gene selection.

Supplementary Figure 2: Enrichment analysis of differentially expressed genes in the bladder from the SCI time course.

Hierarchical clustering of enrichment analyses using **(A)** gene ontology terms for molecular function (GO-MF) **(B)** KEGG pathways and **(C)** gene ontology terms for cellular compartment (GO-CC). **(D)** Bar plot summarizing the count of DNA damage-associated GO terms identified in different datasets comparing SCI versus control rat bladders at 2, 8, and 16 weeks after SCI. The GO terms are categorized into the following groups: Biological Processes (BP): Indicated by **blue bars,** Cellular Components (CC*)*: Indicated by **red bars,** Molecular Functions (MF): Indicated by **gray bars**. The x-axis represents the dataset and GO category, while the y-axis represents the count of DNA damage-related GO terms.

Supplementary Figure 3. Canonical pathway analysis of differentially expressed genes (DEGs) in the bladder from the SCI time course.

Pathways enriched at 2 wks **(A)**, 8 wks **(B)**, and 16 wks **(C)** following SCI. Bar graph shows the -log10(p-value) of pathway enrichment for DEGs compared to control bladders. Pathways are categorized by predicted activity using z-scores: positive z-score (purple) indicates up-regulated pathways, negative z-score (green) indicates down-regulated pathways, and white bars indicate pathways with no predicted activity pattern available. The top pathways include those related to wound healing (GP6 signaling pathway, Wound healing) and innervation (Axonal Guidance, Neurotrophin/TRK signaling). This analysis provides insights into the temporal regulation of signaling pathways in response to SCI, showing the progression of molecular processes, including wound healing, cellular remodeling, fibrosis and inflammation in the bladder.

Supplementary Figure 4: Overview of Transcriptome Analysis

(A)-(C) Visualization of experimental conditions and samples subjected to RNA sequencing. Detrusor and mucosa were microdissected from age-matched controls **(A)**, spinal cord-injured (SCI) rats treated with vehicle **(B),** or SCI rats treated with inosine **(C)** at 8 weeks after injury. Panels B & C also summarize results from our previous studies, highlighting increased non-voiding contractions (SNVC) and reduced staining for synaptophysin (SYP) and the Aδ fiber marker NF200 in the bladder following SCI (B) and mitigation of these changes with inosine treatment (C). Three comparisons are made within detrusor **(D)** or mucosa **(E)** to address i) the effect of SCI on the transcriptome compared to control (SCI-Vehicle vs Control); ii) the effect of inosine treatment compared to vehicle treatment on the transcriptome post-SCI (SCI-Inosine vs Control); and (iii) the impact of inosine treatment post-SCI on the transcriptome compared to control (SCI-Inosine vs Control).

Supplementary Figure 5. Differentially expressed genes (DEGs) in detrusor and their regulation by inosine.

Volcano plot of DEGs in detrusor of SCI-Vehicle versus control **(A)**, SCI-Inosine versus control **(C),** or SCI-Inosine versus SCI-Vehicle **(E)**. Scatterplot of DEGs in detrusor of SCI-Vehicle versus control **(B)**, SCI-Inosine versus control **(D)**, or SCI-Inosine versus SCI-Vehicle **(F)**. In the volcano plots, the horizontal dotted line represents the cutoff for statistical significance (p-value < 0.05), while the vertical dotted lines indicate a logFC of -2 and 2, representing the thresholds for down- and upregulation, respectively. The

scatterplots show logFC (fold change) on the x-axis and logCPM (counts per million) on the y-axis, where each point represents a gene. The position of each point is determined by its logFC value and logCPM, with a gradient in color representing the p-value. Labeled genes in the scatterplot represent those that are statistically significant, biologically relevant, and strongly expressed as represented by p-value, logFC and logCPM, respectively.

Supplementary Figure 6. Differentially expressed genes (DEGs) in mucosa.
Volcano plot of DEGs in detrusor of SCI-Vehicle versus control **(A)**, SCI-Inosine versus control **(C),** or SCI-Inosine versus SCI-Vehicle **(E)**. Scatterplot of DEGs in detrusor of SCI-Vehicle versus control **(B)**, SCI-Inosine versus control **(D)**, or SCI-Inosine versus SCI-Vehicle **(F)**. Details of volcano plots and scatterplots are as described in the legend for Supplementary Figure 5.

Supplementary Figure 7. Expression levels of inosine-responsive genes in detrusor and mucosa
Box chart depicting the expression levels of inosine-responsive genes in the detrusor **(A)** or mucosa **(B)**. The groups include control bladder samples (blue), samples from SCI-Vehicle (pink), and samples from SCI-Inosine (orange). N=3 biological replicates for each condition.

Supplementary Figure 8. Circos plot of the top 20 pathways (based on p-value) and the enriched genes in detrusor from all three comparisons.
The plot is a circular diagram that consists of several components: Sectors: The outer circle is divided into sectors, each representing a pathway or a molecule. These sectors are labelled with the names of the pathways and molecules. Links (chords): The lines (chords) connecting the sectors represent relationships between molecules (proteins) and pathways. Top 20 pathways and enriched genes from analysing DEGs in the detrusors of SCI-Vehicle compared to Control **(A)**; SCI-Inosine compared to SCI-Vehicle **(B)**; SCI-Inosine versus Control **(C)**.

Supplementary Figure 9. Circos plot of the most recurrent genes in the top 20 pathways (based on pvalue) in detrusor from all comparisons.
Top 20 pathways and enriched genes from analysing DEGs in the detrusors of SCI-Vehicle compared to Control **(A)**; SCI-Inosine compared to SCI-Vehicle **(B)**; SCI-Inosine versus Control **(C)**. The plot was created as described in the legend to Supplementary Figure 8.

Supplementary Figure 10. Circos plot of the top 20 pathways (based on p-value) and the enriched genes in mucosa from all three comparisons.

Top 20 pathways and enriched genes from analysing DEGs in the mucosa of SCI-Vehicle compared to Control **(A)**; SCI-Inosine compared to SCI-Vehicle **(B)**; SCI-Inosine versus Control **(C)**. The plot was created as described in the legend to Supplementary Figure 8.

Supplementary Figure 11. Circos plot of the most recurrent genes in the top 20 pathways (based on pvalue) in mucosa from all comparisons.

Top 20 pathways and enriched genes from analysing DEGs in the mucosa of SCI-Vehicle compared to Control **(A)**; SCI-Inosine compared to SCI-Vehicle **(B)**; SCI-Inosine versus Control **(C)**. The plot was created as described in the legend to Supplementary Figure 8.

Supplementary Figure 12. Expression of PAR and γH2AX in human neurogenic bladder tissues.

Representative 20X and 100X images of bladders from pediatric patients with neurogenic bladder or control tissues were stained for **(A)** Poly/Mono ADP Ribosylation (PAR) (green) and Smooth Muscle Actin (SMA) (red) or **(B)** gammaHistone2AX (γH2AX) (green) and neuropilin 2 (NRP2), the latter serving as a marker for smooth muscle. Quantification of PAR and γH2AX **(C & E)** positive nuclei as a percent and **(D & F)** signal intensity. N = 3 biological replicates. A one-way ANOVA was performed followed by a multiple pairwise-comparison between the means of groups using the Tukey Honest Significant Differences. * p 0.05.

Supplementary Figure 13. Outline of the process and output of the in-house developed Image analysis macro.

A) The nuclear ROIs are outlined in green or red if they appear within regions positive for the secondary marker and are superimposed on to the channel containing signal from the primary marker. **B)** A box plot illustrates the average number of nuclei captured in all images from each of three biological replicates. **C)** A histogram is created to visualize the signal intensity in all nuclei captured in all images and used to set a threshold for positive signal. **D)** A scatter plot depicts the area threshold and positive signal threshold used to filter nuclear ROIs (vertical and horizontal dashed lines respectively). **E)** Scatter plots were generated for each condition to visualize the nuclear signal for each biological replicate.

Supplementary Figure 14. In vitro model of oxidative DNA damage recapitulates aspects of SCI model.

(A) Lysates of rat bladder fibroblasts stimulated with PBS (Veh) or increasing concentrations of H_2O_2 for 1 hr were immunoblotted with antibodies to DNA damage markers including phospho-ATM serine/threonine kinase (pATM), gamma histone 2 AX (γH2AX), and Poly/Mono ADP Ribosylation (PAR). **B)** Expression of DNA damage related genes was assessed at 24hr following exposure to the indicated doses of H_2O_2. N = 3 biological replicates. **C)** DNA damage was measured by comet assay in rat bladder fibroblasts exposed to the indicated doses of H_2O_2 for 1hr. Images are taken at 20X magnification. **D)** For quantification of the comet assay, 100-200 nuclei were assessed for each condition. Data are representative of 3 independent trials. **E)** Rat bladder fibroblasts stimulated with PBS (Veh) or 50μM H_2O_2 (the lowest dose that achieved a comet) for 1 hour were assessed for malondialdehyde (MDA), a measure of lipid peroxidation. N = 3 biological replicates. A one-way ANOVA was performed followed by a multiple pairwise-comparison between the means of groups using the Tukey Honest Significant Differences. Adjusted p-values were used to report the significance of the differences. * p 0.05, ** p 0.01, comparing to the PBS control (Veh).

Supplementary Figure 15. Integration of transcriptomics and proteomics.

(A) Illustration of the integration of transcriptomics and proteomics data as a venn diagram that showcases the intersection of pathways between whole bladder proteomics data obtained at 8 weeks following SCI and RNA sequencing data from both the detrusor and mucosa tissues. This visual representation highlights the commonalities and overlaps in biological pathways between these two types of molecular data. **(B)** Temporal expression of Purine Nucleoside Phosphorylase (PNP) was assessed with SCI: This violin plot depicts the log2 fold change in PNP expression in rat bladder tissue over a 16-week period after SCI. Data points for both control (pink) and SCI (yellow) groups are displayed at 2-, 8-, and 16-weeks post-injury, with the spread indicating variability within the groups. A statistically significant change (p=0.02) in PNP expression was observed between the control and SCI groups, reflecting the impact of injury on this specific protein involved in purine metabolism.

Supplementary Figure 16. Expression Profiles of ribosomal genes in detrusor and mucosa of rats with SCI.

In the data preprocessing workflow, ribosomal genes were selectively identified and extracted from RNA sequencing datasets. The dataset was further refined to include only these ribosomal genes, ensuring that

genes with statistical significance (p-value < 0.05) and a substantial change in expression (absolute log-fold change > 0.5) were retained. Expression values across different replicates and conditions (Control, SCI treated with vehicle or inosine in Detrusor (A) or Mucosa (B), were selected.

Supplementary Figure 17. Full, uncropped immunoblots of DNA damage-related proteins in rat bladder cell lysates. Rat bladder cell lysates generated following exposure to PBS-vehicle (Veh), H_2O_2 (50μM, 125μM), and/or Inosine (250μM, 1mM) for 24hrs were immunoblotted with antibodies to (**A)** Beta actin (Actb), (**B)** phosphorylated ATM kinase (pATM), (**C)** gamma histone 2AX (γH2AX) or (**D)** Poly/mono ADP Ribosylation (PAR).

Supplementary Table 1. Reagent information

(A) Antibody Information. **(B)** Sequences of PCR primers.

Table 1A: Primer Sequences

#	Target	Species	Forward Sequence	Reverse Sequence
1	Rnf8	Rat	TAGGGAACGAAGAGCAAAGAGA	GAATCCTCTAGAACGCGCCA
2	Mad2l2	Rat	ACTTCGGACATTTTAAAGATGCAA	CGGTTAACGACAATGCGGTC
3	Trrap	Rat	TAAAGCTCCTGGTGAAGCTCAG	AGCGAACAGGGCAGAAGTAG
4	Chk2	Rat	CAACTACTGGTTCGGGAGGG	AGGGCCCATTTCCCTGAAGA
6	Rad50	Rat	GACTGTCAGCGTGAACTGGA	GTTGTAGACGACCCTGCTCC
7	Xpc	Rat	GAACGCCTCTGGCTAGCAT	CTTCAAAGTCGTCCGCATCG
10	RPS18	Rat	ACAACGTGACTCCAGACATGA	GCCTTCTTTTGAGCAACCCG
11	BACT	Rat	ACAACCTTCTTGCAGCTCCTC	CCTTCTGACCCATACCCACC

Table 1B: Antibodies

**Antibody**	**Vendor**	**Catalog number**	**Host species**	**IF Dilution**	**IB Dilution**	**RRID**
γH2AX	Abcam	ab26350	Mouse	1:100	1:1000	RRID:AB_470861
PAR (E6F6A)	CST	83732	Rabbit	1:100	1:1000	RRID:AB_2749858
p-ATM/ATR substrate	CST	2851S	Rabbit	1:100	1:1000	RRID:AB_330318
SM22α	CST	40471S	Rabbit	1:100	1:1000	RRID:AB_3675872
α-SMA	Sigma	A2547	Mouse	1:100	1:1000	RRID:AB_476701
NRP2	Sigma	HPA054974	Rabbit	1:100	N/A	RRID:AB_3676762
Pan-cytokeratin	Sigma	C2562		1:100	1:1000	RRID:AB_476839
p-ATM	Invitrogen	MA1-46069		1:100	1:1000	RRID:AB_2062982
β-actin	Sigma	A5441		1:100	1:10000	RRID:AB_476744
Anti-Rabbit Alexa-488	ThermoFisher	A-21206	Donkey	1:1000	N/A	RRID:AB_2535792
Anti-Rabbit Alexa-594	ThermoFisher	A-21207	Donkey	1:1000	N/A	RRID:AB_141637
Anti-Mouse Alexa-488	ThermoFisher	A-21202	Donkey	1:1000	N/A	RRID:AB_141607
Anti-Mouse Alexa-594	ThermoFisher	A-21203	Donkey	1:1000	N/A	RRID:AB_2535789
